# Binding Small Molecules to a *cis*-Dicarbonyl ^99^Tc^I^-PNP Complex via Metal–Ligand Cooperativity

**DOI:** 10.1021/acs.inorgchem.3c01177

**Published:** 2023-06-23

**Authors:** Manuel Luca Besmer, Henrik Braband, Thomas Fox, Bernhard Spingler, Alfred P. Sattelberger, Roger Alberto

**Affiliations:** † Department of Chemistry, University of Zurich, Zurich CH-8057, Switzerland; ‡ Department of Chemistry, University of Central Florida, 4111 Libra Drive, Orlando, Florida 32816, United States

## Abstract

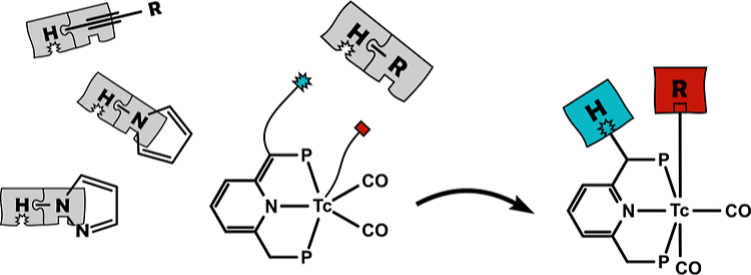

Metal–ligand cooperativity is a powerful tool for the activation of various bonds but has rarely, if ever, been studied with the radioactive transition metal ^99^Tc. In this work, we explore this bond activation pathway with the dearomatized PNP complex *cis*-[^99^Tc^I^(^Pyr^PNP^*t*Bu^*)(CO)_2_] (**4**), which was synthesized by deprotonation of *trans*-[^99^Tc^I^(^Pyr^PNP^*t*Bu^)(CO)_2_Cl] with KO^*t*^Bu. Analogous to its rhenium congener, the dearomatized compound reacts with CO_2_ to form the carboxy complex *cis*-[^99^Tc^I^(^Pyr^PNP^*t*Bu^–COO)(CO)_2_] and with H_2_ to form the mono-hydride complex *cis*-[^99^Tc^I^(^Pyr^PNP^*t*Bu^)(CO)_2_H] (**7**). Substrates with weakly acidic protons are deprotonated by the Brønsted basic pincer backbone of **4**, yielding a variety of intriguing complexes. Reactions with terminal alkynes enable the isolation of acetylide complexes. The deprotonation of an imidazolium salt results in the in situ formation and coordination of a carbene ligand. Furthermore, a study with heterocyclic substrates allowed for the isolation of pyrrolide and pyrazolide complexes, which is uncommon for Tc. The spectroscopic analyses and their solid-state structures are reported.

## Abstract 2

Deprotonation of *trans*-[^99^Tc^I^(^Pyr^PNP^*t*Bu^)(CO)_2_Cl] with KO^*t*^Bu in tetrahydrofuran yields the highly reactive, dearomatized *cis*-[^99^Tc^I^(^Pyr^PNP^*t*Bu^*)(CO)_2_], which readily reacts with dihydrogen (H_2_) and carbon dioxide (CO_2_) as its rhenium homologue. The observed reactivities with small molecules via metal−ligand cooperativity are extended to substrates comprising weakly acidic protons, which are deprotonated by the pincer backbone of the reactive complex.

## Introduction

**Scheme 1:**
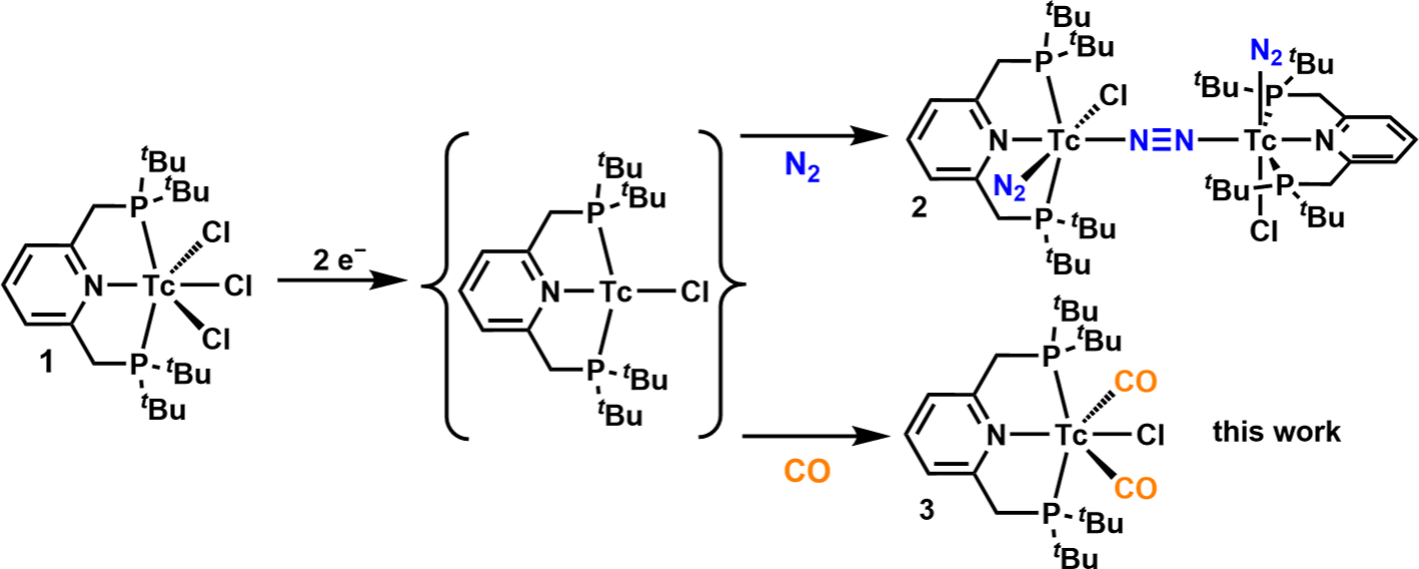
Two-Electron Reduction of **1** to the Electronically Unsaturated Intermediate [^99^Tc^I^(^Pyr^PNP^*t*Bu^)Cl], which Coordinates N_2_ to Yield **2** or CO for **3**(17)

Pincer-type ligands and their transition metal complexes have been established as highly versatile compounds in various catalytic processes.^1−6^ Especially pyridine- and acridine-based PNP ligands, in combination with a multitude of metal centers, find applications in diverse bond activation processes.^7−15^ Their chemistries in combination with ^99^Tc is though limited due to its radioactive nature (β^–^ decay with *E*_max_ = 293 keV, half-life: ∼2 × 10^5^*y*).^16^ Only recently we described ^99^Tc-dinitrogen complexes employing two structurally related PNP pincer ligands.^17^ The Tc^III^ complex (**1**), comprising the 2,6-bis((di-*tert*butylphosphino)methyl)pyridine ligand (^Pyr^PNP^*t*Bu^), was reduced to Tc^I^, resulting in the coordinatively unsaturated compound [^99^Tc^I^(^Pyr^PNP^*t*Bu^)Cl]. If this reduction is carried out under an N_2_ atmosphere, the dinuclear [^99^Tc^I^(^Pyr^PNP^*t*Bu^)(N_2_)Cl]_2_(μ-N_2_)] complex forms (Scheme 1). We highlighted distinct differences between PNP complexes of technetium and its higher homologue rhenium^18^ in presence of N_2_. Rhenium pincer chemistry has been extensively explored, and benchmark catalysts for photochemical, electrochemical, and thermal N_2_ fixation have been reported.^19^ The radioactive nature of ^99^Tc generates a knowledge gap in comparison to its nearest neighbors, especially for uncommon complexes such as those found in bond activation processes. The situation is different for the metastable nuclear isomer ^99m^Tc, which is an integral part of nuclear medicine in imaging.^20,21^

Among the diverse efforts in the field of small molecule activation (SMA), the findings on metal–ligand cooperativity (MLC) represent an opportunity for alternative activation pathways. The first examples of dearomatization/rearomatization cooperativity were reported by Gunanathan and Milstein in 2011 with pyridine-based pincer ligands coordinated to Ru, Ir, and Pt.^22^ This type of MLC offers potential for the design of catalysts for globally relevant chemical transformations in a greener fashion. In general, MLC allows for bond activation in substrates while the oxidation state of the metal center remains unchanged. Published results reveal activation of small substrate molecules such as H_2_,^23^ C_6_D_6_,^12^ alcohols^24^ and amines.^25^ The covalent binding of CO_2_ to transition metal complexes has been reported via a variety of pathways, such as bifunctional reactivity,^26−28^ ligand-centered reactivity,^29−31^ C–H bond insertion,^32^ MLC with a bidentate aminopyridine ligand,^33^ and MLC with dearomatized pyridine PNP ligands.^34,35^ The pyridine-based PNP ligand (^Pyr^PNP^*t*Bu^) is prevalent in MLC chemistry, where dearomatization–rearomatization of the pyridine moiety forms the basis of the cooperativity. The rhenium complex *cis*-[Re(^Pyr^PNP^*t*Bu^)(CO)_2_Cl] has been employed as a platform for dearomatization and subsequent substrate activations by Milstein and co-workers. Deprotonation of this compound leads to the formation of the complex *cis*-[Re(^Pyr^PNP^*t*Bu^*)(CO)_2_] (the asterisk indicates the dearomatized PNP ligand), which reacts with nitriles via an addition reaction to form ketimido or enamido complexes, and its catalytic properties for Michael addition were established.^34^ The loss of aromaticity (dearomatization in the following means conversion of the central pyridine ligand to N-deprotonated 1,2-dihydro-2-methylene pyridine) in the pyridine moiety upon deprotonation is evidenced by a large upfield shift of its protons, while the metal center stabilizes the dearomatized ligand.

**Scheme 2:**
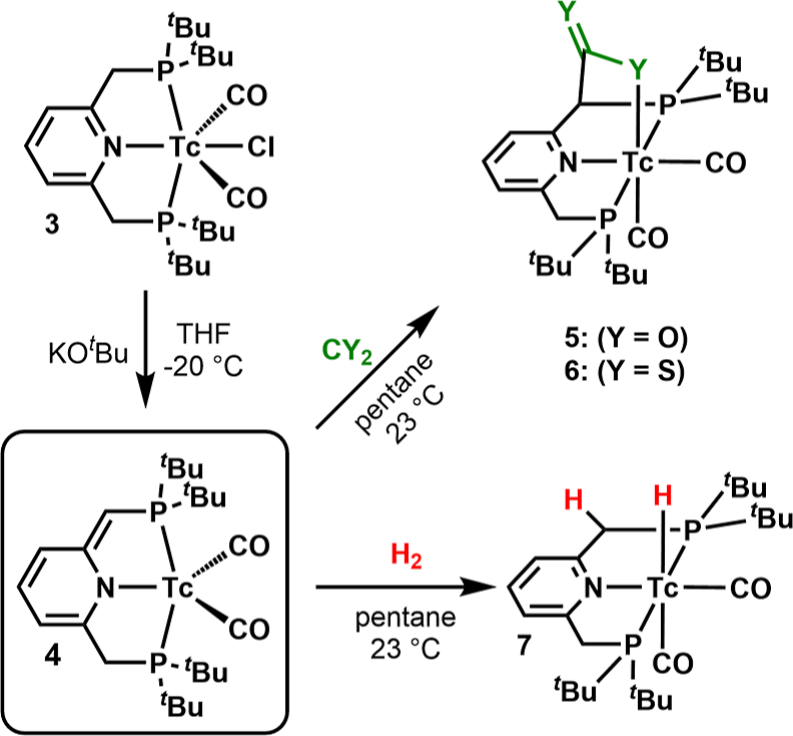
Synthesis of Dearomatized [^99^Tc(^Pyr^PNP^*t*Bu^*)(CO)_2_] (**4**) by Deprotonation of **3** with KO^*t*^Bu and Its Reaction with CO_2_, CS_2_, and H_2_ to Form the Tc–Y–CY–PNP Complexes (Y = O, **5**; Y = S, **6**) and Monohydride **7**

We describe herein the synthesis of a technetium dicarbonyl complex via two-electron reduction of [^99^Tc^III^(^Pyr^PNP^*t*Bu^)Cl_3_] (**1**) and subsequent coordination of two CO ligands to the electronically unsaturated, non-isolated [^99^Tc^I^(^Pyr^PNP^*t*Bu^)Cl] intermediate (Scheme 1). Deprotonation of **3** leads to the formation of the related dearomatized complex [^99^Tc^I^(^Pyr^PNP^*t*Bu^*)(CO)_2_] (**4**, Scheme 2). The transfer of the established MLC chemistry with Re to ^99^Tc with small substrate molecules, including H_2_ and CO_2_, is described. Furthermore, the reactivities with new substrate classes, such as terminal alkynes, imidazolium ions, and heterocycles, have been investigated.

## Results and Discussion

*Note*: ^99^Tc is a weak β^–^ emitter. All experiments were carried out in licensed and appropriately shielded laboratories designed for work with low-level radioactive materials.

### Synthesis of *trans*-[^99^Tc(^Pyr^PNP^*t*Bu^)(CO)_2_Cl] (**3**) and Dearomatized **4**

We reported the synthesis of [^99^Tc^III^(^Pyr^PNP^*t*Bu^)Cl_3_] (**1**) from the precursor *mer*,*trans*-[^99^Tc^III^Cl_3_(PPh_3_)_2_(MeCN)], which is the starting material for all subsequent reactions. The reduction of **1** with KC_8_ in THF under a CO atmosphere resulted in a deep red suspension. THF was evaporated under a stream of argon. The residue was extracted with benzene, and crystallization gave *trans*-[^99^Tc^I^(^Pyr^PNP^*t*Bu^)(CO)_2_Cl] (**3**) in ∼70% yield (Scheme 1).

**Figure 1:**
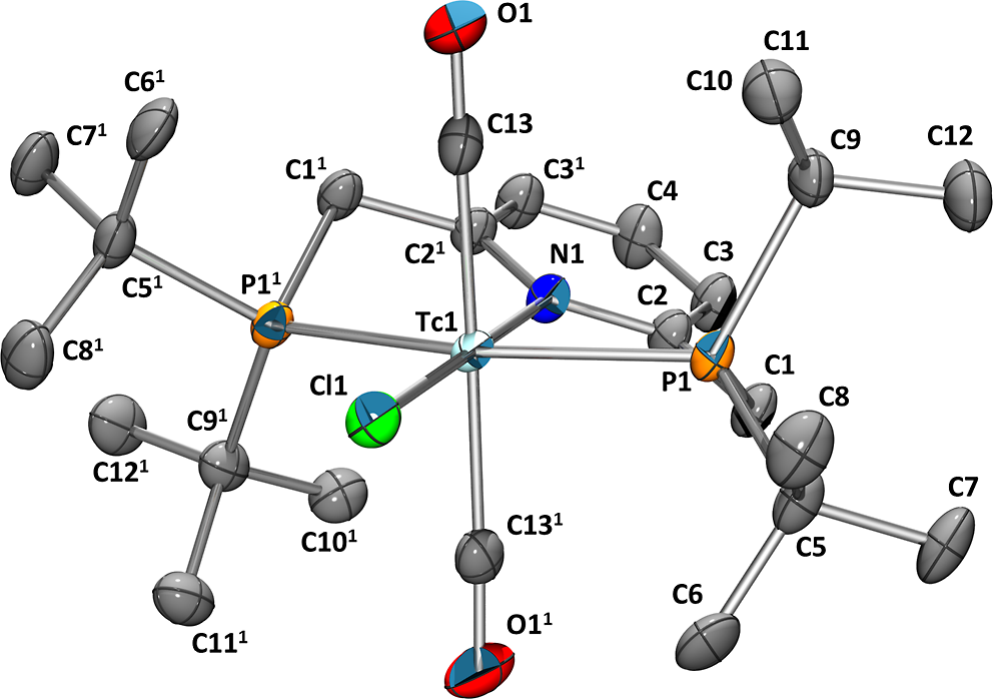
Ellipsoid displacement plot^37^ of *trans*-[^99^Tc(^Pyr^PNP^*t*Bu^)(CO)_2_Cl] (**3**). Ellipsoids represent a 35% probability. Hydrogen atoms are omitted for clarity. Selected bond lengths (Å) and angles (°): Tc1–P1 2.4092(7), Tc1–N1 2.136(3), Tc1–C13 1.983(3), Tc1–Cl1 2.5261(10), C13–O1 1.109(4), C1–C2 1.498(4), C13–Tc1–C13^1^ 176.76(19), P1–Tc1–P1^1^ 159.43(4), Cl1–Tc1–P1 100.287(18), and Cl1–Tc1–C13 88.38(10).

The deep red complex **3** is the second example of small molecules coordinating to the [^99^Tc^I^(^Pyr^PNP^*t*Bu^)Cl] framework upon reduction from +III to +I (**2**, Scheme 1). The IR spectrum of **3** showed two main bands, ν_CO_ at 1952 and 1867 cm^–1^ in a 1:2 ratio. The more intense band is assigned to the two *trans* CO ligands, while the presence of the second band suggests the *cis* isomer in the product. We did not attempt to separate the two complexes at this stage since the relative coordination of the two COs is not essential for the follow-up reactions; however, it gives an insight into the CO coordination mechanism after the reduction. The *trans* isomer **3** crystallized from benzene in the hexagonal space group *P*6_1_22. The X-ray crystal structure analysis shows a distorted octahedral geometry induced by the bite angle of 159.43(4)° of the meridionally coordinated ^Pyr^PNP^*t*Bu^ ligand (Figure 1). The bite angle of the axially symmetric structure **3** is almost the same as the one of the starting material **1** (159.84(3)°).^17^ Furthermore, the ^99^Tc NMR spectrum of **3** shows a broad singlet at δ −1266 ppm (Δ_1/2_ = 337 Hz), comparable to the chemical shift reported for the Tc^I^ complex [^99^TcBr(CO)_3_(PPh_3_)_2_] at δ −1460 ppm (Δ_1/2_ = 500 Hz).^36^

**Figure 2:**
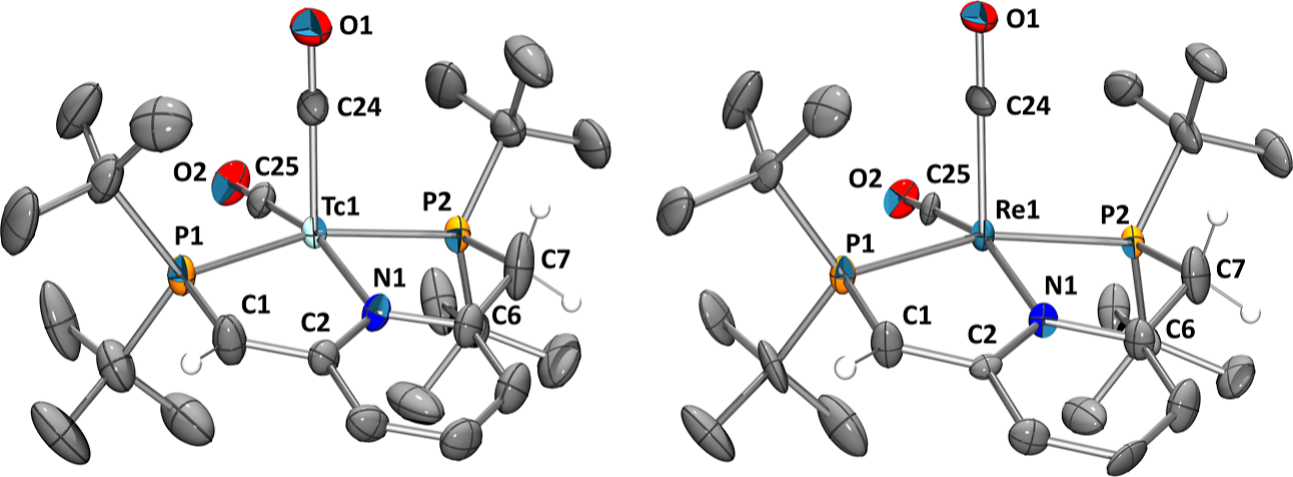
Ellipsoid displacement plots^37^ of [^99^Tc(^Pyr^PNP^*t*Bu^*)(CO)_2_] (**4**, left) and the homologous [Re(^Pyr^PNP^*t*Bu^*)(CO)_2_] (right). Ellipsoids represent a 35% probability. Hydrogen atoms, except for the methine and methylene groups, are omitted for clarity.

Treatment of a solution of **3** in THF with KO^*t*^Bu (or potassium bis(trimethylsilyl)amide) at −20 °C results in a color change from deep red to green over the course of 30 min. Removal of solvent, extraction with *n*-pentane, and crystallization yielded the dearomatized complex **4** as a dark green solid. The deprotonation at one of the methylene groups of the PNP ligand results in the disruption of aromaticity in the former pyridine moiety, accompanied by the loss of the chloride ligand and the formation of [^99^Tc^I^(^Pyr^PNP^*t*Bu^*)(CO)_2_] (**4**, Scheme 2). Complex **4** adopts a structure similar to its heavier rhenium homologue.^34^ The spectroscopic analysis of **4** is as expected. In the IR spectrum of solid **4**, two intense bands are observed at 1926 and 1845 cm^–1^ in a 1:1 ratio, assigned to the two *cis* carbonyl ligands. These values both correspond to an approximate blue shift of 20 cm^–1^ for **4** as compared to rhenium with identical, relative band intensities. The ^1^H NMR signal for the methine proton of the dearomatized PNP ligand shows up as a doublet at δ 3.76 ppm (^2^*J*_HP_ = 2.76 Hz, 1H). The unscathed methylene group of the second pincer arm appears as a doublet at δ 2.74 ppm (^2^*J*_HP_ = 9.03 Hz, 1H). The three proton signals of the dearomatized pyridine moiety give rise to a multiplet at δ 6.38 ppm (2H, C*H*_pyr(4,5)_) and a doublet at δ 5.41 ppm (1H, C*H*_pyr(3)_). These signals corroborate the dearomatization of the pyridine moiety. In the ^31^P{^1^H} NMR spectrum, the two chemically inequivalent phosphorus nuclei were observed as two very broad signals at δ 80.44 (Δ_1/2_ = 524 Hz) and 70.67 ppm (Δ_1/2_ = 513 Hz). The line broadening of the signals is a result of the coupling of the phosphorus nuclei to ^99^Tc with a nuclear spin of 9/2. The carbon nuclei of the methine and methylene groups were observed as doublets in the ^13^C{^1^H} NMR at δ 68.3 and 35.7 ppm, respectively. The resonances for the two *cis* carbonyl ligands were not observed due to the high quadrupolar moment and 9/2 spin of ^99^Tc. Together with the coupling to both phosphorus nuclei, this leads to an enormously broadened signal. The ^99^Tc resonance was observed as a very broad signal at δ −1103 ppm (Δ_1/2_ = 3.4 kHz), in the same region as the *cis*-dicarbonyl compound [^99^Tc(η^2^-O,S–Et_2_btu)(CO)_2_(PPh_3_)_2_] (δ −1119 ppm (Δ_1/2_ = 2.5 kHz)).^38^ Compound **4** was crystallized from a saturated pentane solution at −20 °C and analyzed by single-crystal X-ray diffraction. The structure is depicted in Figure 2 and compared to the homologous rhenium structure reported by Vogt et al.^34^ The single-crystal structure of **4** reveals the mutual *cis* orientation of the two CO ligands (C24–Tc1–C25: 87.2(2)°), as suggested by the 1:1 ratio of the carbonyl bands in the IR spectrum. The bite angles of the PNP ligands of both structures are nearly identical, with 160.36(5)° for Tc and 160.57(9) for Re. The bond lengths of 1.390(6) Å for the C1–C2 bond and 1.482(8) Å for the C6–C7 bond reveal double bond formation after dearomatization. This loss in symmetry is further evidenced by the P1–C1–C2–N1 and P2–C7–C6–N1 dihedral angles that were determined to be 9.0(6) and −29.0(7)°, respectively. The Tc1–N1 bond of the formally negatively charged PNP ligand of **4** has a bond length (2.163(3) Å), which is comparable to the one of the starting material **3** (2.136(3) Å).

### Reactions of *cis*-[^99^Tc(^Pyr^PNP^*t*Bu^*)(CO)_2_] (**4**) with CO_2_, CS_2_, and H_2_

Key compound **4** enables the exploration of its reactivity toward small molecules and its comparison with rhenium. Milstein and co-workers reported on the activation of CO_2_ via MLC with Ru and Re PNP complexes,^35,39^ which inspired us to explore the equivalent reaction with ^99^Tc. Dearomatized **4** was freshly prepared, dissolved in pentane, and exposed to CO_2_ gas via a syringe at 23 °C, which resulted in an immediate color change from green to pale yellow and precipitation of the product. Evaporation of the solvent afforded *cis*-[^99^Tc^I^(^Pyr^PNP^*t*Bu^–COO)(CO)_2_] (**5**, Scheme 2) as a pale yellow solid. The CO_2_ binding proceeds as a [1,3]-addition to the −Tc–P–CH– moiety. The reaction results in the formation of a C–C bond between the carbon dioxide molecule and the PNP backbone methine group, the formation of a Tc–O bond, and the rearomatization of the pyridine ring. The ^1^H NMR spectrum provides evidence for the rearomatization and for the asymmetry of the PNP ligand as the pyridine protons appear as three separate signals at δ 6.78 ppm (*t*, ^3^*J*_HH_ = 7.78 Hz, 1H, C*H*_pyr(4)_), 6.73 ppm (d, ^3^*J*_HH_ = 7.76 Hz, 1H), and 6.34 ppm (d, ^3^*J*_HH_ = 7.70 Hz, 1H). The asymmetry is further obvious in the ^31^P{^1^H} NMR where two strongly broadened signals are observed at δ 113.2 (Δ_1/2_ = 5.3 kHz) and 91.1 ppm (Δ_1/2_ = 1.8 kHz). These spectroscopic features coincide with the data reported for rhenium, and the ^13^C{^1^H} NMR resonance of the carboxylate carbon (Tc–O–*C*O–C) has a comparable shift of δ 170.4 ppm (dd, ^2^*J*_CP_ = 10.11 Hz, ^4^*J*_CP_ = 1.74 Hz, 1C) to the Re analogue (172.8 ppm, ^2^*J*_CP_ = 10.4 Hz, ^4^*J*_CP_ = 2.7 Hz). The ^99^Tc NMR revealed that the signal for **5** was slightly downfield shifted with respect to the starting material **4** at δ −1061 ppm (Δ_1/2_ = 2.3 kHz). The two *cis* CO ligands of **5** give rise to two bands in the IR spectrum at 1929 and 1848 cm^–1^ in a 1:1 ratio, consistent with their *cis* orientation. The intense band at 1648 cm^–1^ is indicative of the carboxylate moiety.

**Figure 3:**
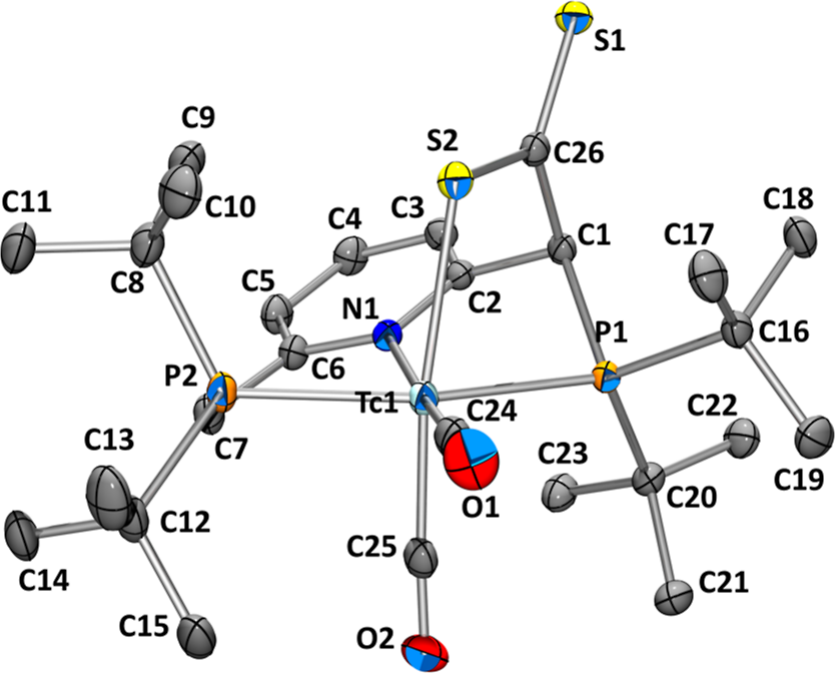
Ellipsoid displacement plot^37^ of [^99^Tc(^Pyr^PNP^*t*Bu^–CSS)(CO)_2_] × PPh_3_ (**6**). Ellipsoids represent a 35% probability. Hydrogen atoms and the co-crystallized PPh_3_ molecule are omitted for clarity. Selected bond lengths (Å) and angles (°): Tc1–P1 2.4296(4), Tc1–P2 2.4303(4), Tc1–N1 2.1743(12), Tc1–C24 1.9000(16), Tc1–C25 1.8918(17), C24–O1 1.158(2), C25–O2 1.161(2), Tc1–S2 2.4997(4), S2–C26 1.6982(14), S1–C26 1.6637(14), C24–Tc1–C25 91.39(7), S1–C26–S2 124.31(9), C1–C26–S2 116.26(10), Tc1–S2–C26 102.64(5), and P1–Tc1–S2 79.714(12).

The binding of CO_2_ via MLC in complex **5** illustrates basic similarities in the reactivities of homologous Re^I^ and Tc^I^ complexes and is the first example of CO_2_ directly binding to a Tc complex. Single crystals for X-ray diffraction could not be obtained. However, the reaction of **4** with CS_2_ instead of CO_2_ resulted in a rapid color change to dark yellow and the formation of a precipitate. Evaporation of the solvent, extraction with benzene, and subsequent crystallization gave dark yellow *cis*-[^99^Tc^I^(^Pyr^PNP^*t*Bu^–CSS)(CO)_2_] (**6**, Scheme 2). Spectroscopy of the dithiocarboxylate complex **6** evidenced a high similarity to **5** with CO_2_ implying an equivalent [1,3]-addition as with CO_2_. The pyridine protons appear as a set of two doublets and a triplet in the ^1^H NMR spectrum, and the methine proton of the pincer backbone appears as a doublet of doublets at δ 5.89 ppm (dd, ^2^*J*_HP_ = 6.63 Hz, ^4^*J*_HP_ = 1.48 Hz, 1H). The phosphorus nuclei were observed in a similar region as in **5**. The ^13^C{^1^H} NMR signal of the PNP–*C*S–S–Tc moiety shifted upfield as compared to **5**, overlaid with the signal of a quaternary pyridine carbon, consistent with the lower electronegativity of sulfur relative to oxygen. In the ^99^Tc NMR spectrum, the signal for **6** was found as an upfield-shifted resonance of δ −1282 ppm (Δ_1/2_ = 1.9 kHz). The stronger color of **6** over **5** is evidenced by the more intense molar absorption coefficient at λ_max_ 428 nm (ε = 4317) which is bathochromically shifted [**5**, λ_max_ 397 nm (ε = 400)]. The IR spectrum shows ν_CO_ in an approximate 1:1 ratio at 1934 and 1872 cm^–1^. Crystals grown from a saturated toluene solution at −20 °C allowed for a solid-state structure analysis of **6** (Figure 3).

The solid-state structure of the dithiocarboxylate **6** reveals bond lengths for S1–C26 and S2–C26 of 1.6637(14) and 1.6982(14) Å, respectively. This difference of 0.03 Å suggests delocalization of the π electrons in the CSS moiety and matches the values found for a Cu complex comprising a CS_2_ group in a similar binding motif.^40^ The coordination geometry of **6** is a highly distorted octahedron, which is corroborated by the bond angle P1–Tc1–S2 of 79.714(12)°. The two CO ligands are still oriented in an approximate *cis* fashion (C24–Tc1–C25 = 91.39(7)°). The co-crystallized triphenylphosphine originates from the synthesis of starting material **1** and was not removed in the work-up.

**Figure 4:**
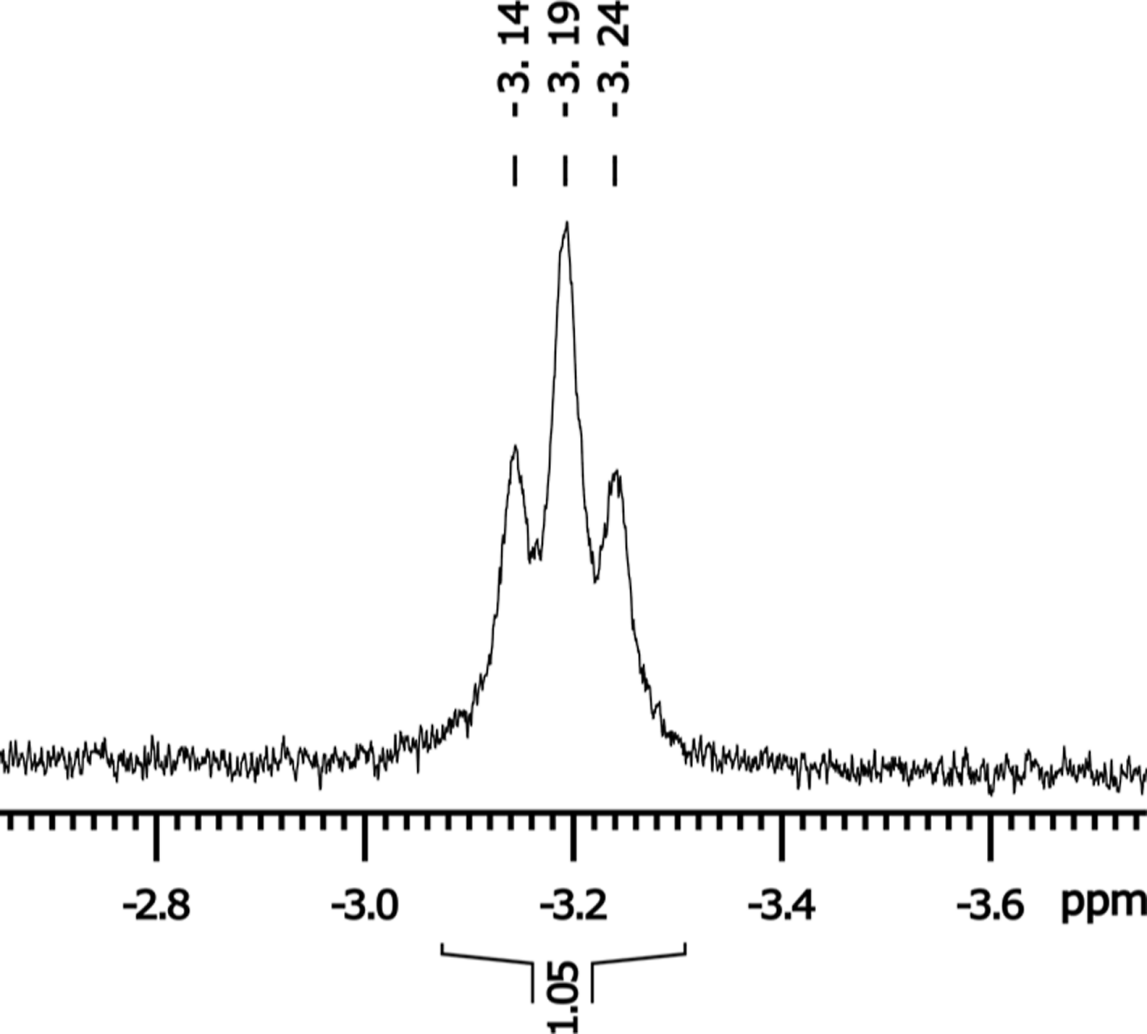
Excerpt of the ^1^H NMR spectrum of the mono-hydride complex **7**, displaying the triplet signal for the Tc–H proton at δ −3.19 ppm (Δ_1/2_ = 62 Hz, full spectra in Supporting Information). The multiplicity arises from the coupling to both chemically equivalent phosphorus nuclei, and the signal is broadened because of the direct coupling to Tc.

The reaction of [Re(^Pyr^PNP^*t*Bu^*)(CO)_2_] with H_2_ leads to a heterolytic cleavage. Following the binding of CO_2_ and CS_2_ to Tc, a pentane solution of **4** was treated with a gentle stream of dihydrogen via a syringe. The green solution immediately turned pale yellow. After evaporation of the solvent, the mono-hydride complex **7** (Scheme 2) was obtained in quantitative yield. The analysis of *cis*-[^99^Tc^I^(^Pyr^PNP^*t*Bu^)(CO)_2_H] by ^1^H NMR evidenced its homologous structure to the rhenium analogue. The heterolytic H_2_ splitting presumably proceeds via initial side-on coordination, polarization, and deprotonation by the methine group. The pincer backbone is protonated, and the four protons appear as a not fully resolved triplet-like singlet at δ 3.03 ppm. The protons of the pyridine moiety appear as a triplet and doublet signal at δ 6.68 ppm (*t*, ^3^*J*_HH_ = 7.68 Hz, 1H, C*H*_pyr(4)_) and 6.37 ppm (d, ^3^*J*_HH_ = 7.70 Hz, 2H, C*H*_pyr(3,5)_), indicating a symmetry plane through the aromatic ring. The hydride appears as a triplet at a characteristic chemical shift for transition metal hydrides of δ −3.20 ppm (*t*, ^2^*J*_HP_ = 24.09 Hz, 1H, Figure 4) due to the coupling to the chemically equivalent phosphorus nuclei of the PNP ligand. The fact that the hydride signal can be observed in the ^1^H NMR, in contrast to the lack of signals for CO ligands in the ^13^C{^1^H} spectra for all complexes, is attributed to a small scalar coupling constant *J* of the ^1^H–^99^Tc pair (applied formulas can be found in the Supporting Information). The identical chemical environment of both phosphorus nuclei is also apparent in the observation of a single peak in the ^31^P{^1^H} NMR at δ 103.2 ppm (Δ_1/2_ = 1.7 kHz). The ^99^Tc NMR resonance was observed at δ −1097 ppm (Δ_1/2_ = 2.7 kHz), in the same range as **4–6**. In the rhenium complex, four methylene protons appear as a pair of doublets of triplets, and a coupling to the Re–H moiety was observed. Interestingly, **7** shows a different multiplicity of the respective signals. The formation of complexes **4–7** underlines similar reactivities of Tc and Re for these small molecules binding via MLC. The often-made (but not always true)^41,42^ observation that Tc and Re complexes behave similarly in low oxidation states is confirmed in this case.

### Alkynes

**Scheme 3:**
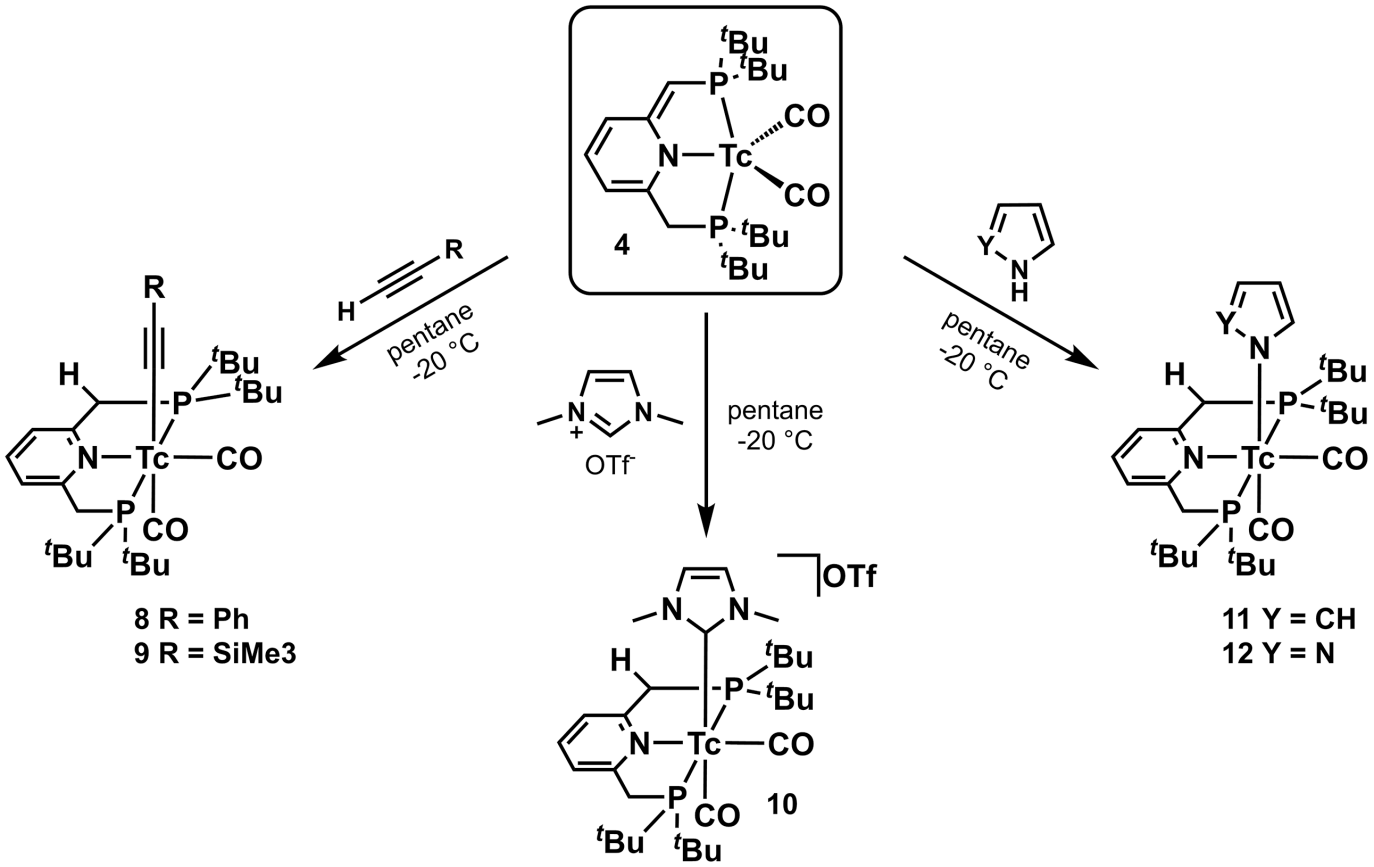
Syntheses of Acetylide Complexes **8** and **9**, Carbene Complex **10**, Pyrrolide Complex **11**, and Pyrazolide Complex **12** from Dearomatized [^99^Tc(^Pyr^PNP^*t*Bu^*)(CO)_2_] (**4**)

The MLC reactivities of dearomatized pincer complexes consist of a concerted electrophilic attack on the methine pincer backbone and the concomitant formation of a metal-substrate bond, e.g., in the case of CO_2_, a carbon–carbon and a Tc–O bond. This reactivity with polarized and unpolarized substrates was applied to the interaction of **4** with terminal alkynes. A solution of phenylacetylene in benzene was added dropwise to **4** in pentane at −20 °C and left to warm to room temperature. After 10 min, the color changed from green to orange. Removal of solvent, extraction with benzene, filtration, and evaporation of solvent yielded a yellow/orange solid. The spectroscopic analysis of the product evidenced the formation of the acetylide complex *cis*-[^99^Tc^I^(^Pyr^PNP^*t*Bu^)(CO)_2_(−C≡C–Ph)] (**8**, Scheme 3) in ∼60% yield.

**Figure 5:**
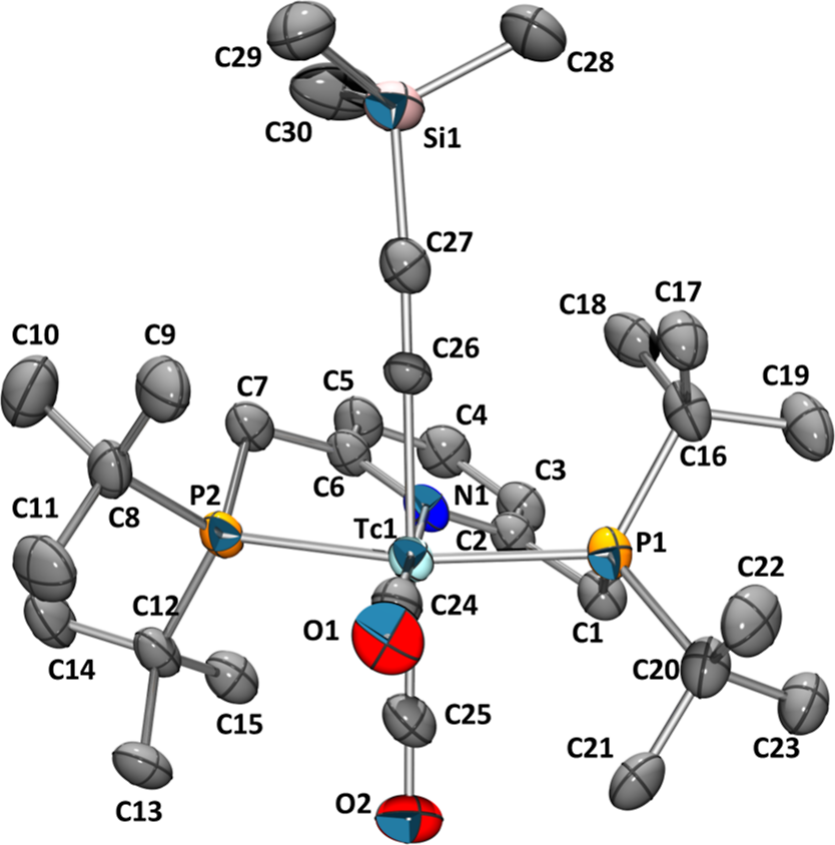
Ellipsoid replacement plot^37^ of *cis*-[^99^Tc(^Pyr^PNP^*t*Bu^)(CO)_2_(−C≡C–TMS)] (**9**). Ellipsoids represent a 35% probability. Hydrogen atoms are omitted for clarity. Selected bond lengths (Å) and angles (°): Tc1–P1 2.4299(15), Tc1–P2 2.4183(15), Tc1–N1 2.192(4), Tc1–C24 1.882(6), Tc1–C25 1.910(6), C24–O1 1.161(7), C25–O2 1.173(7), Tc1–C26 2.132(5), C26–C27 1.211(7), C24–Tc1–C25 88.9(2), Tc1–C26–C27 174.3(5), P1–Tc1–C26 90.63(15), and P2–Tc1–C26 85.22(15).

Following this procedure, the trimethylsilyl (TMS) acetylide complex *cis*-[^99^Tc^I^(^Pyr^PNP^*t*Bu^)(CO)_2_(−C≡C–TMS)] (**9**, Scheme 3) was prepared analogously in a yield of ∼75%. The reactions with both substrates presumably proceed via initial in situ deprotonation of the acetylene by the methine group of the pincer, rearomatization of the pyridine moiety, and subsequent coordination of the acetylide anion. The syntheses of **8** and **9** are only the second examples of technetium(I) acetylide complexes. The work published recently by Roca Jungfer and Abram demonstrated coordination of various R–C≡C– ligands to [^99^Tc^I^(SMe_2_)(CO)_3_(PPh_3_)_2_]^+^.^43^ There, the alkynes were deprotonated with organo-lithium reagents prior to coordination, while in our reaction, complex **4** acts as an internal base and anion acceptor in one. In the ^1^H NMR spectrum, the pyridine protons for **8** and **9** appear as triplets at roughly δ 6.7 ppm (1H, C*H*_pyr(4)_) and doublets at δ 6.4 ppm (1H, C*H*_pyr(3,5)_), indicating reestablished symmetry in the PNP ligand. Furthermore, the methylene and ^*t*^Bu protons are observed at nearly identical chemical shifts in both complexes. The nine TMS protons of **9** appear as a singlet at δ 0.22 ppm and the phenyl protons of **8** as an overlaid doublet at δ 7.40 ppm (2H), a triplet at δ 7.11 ppm (2H), and a triplet at δ 6.94 ppm (1H). These signals indicate free rotation of both the phenyl and the TMS moieties. The ^31^P{^1^H} spectra revealed broad singlets at δ ≈ 92 ppm, corroborating the chemical equivalence of both phosphorus nuclei, comparable to what was found in the mono-hydride complex **7**. The ^29^Si NMR signal for the TMS group was observed at δ −29.31 ppm. The ^99^Tc NMR resonance of the TMS acetylide complex was found as a strongly broadened signal at δ −1435 ppm (Δ_1/2_ = 15.7 kHz), downfield shifted as compared to the published [^99^Tc^I^(−C≡C–TMS)(CO)_3_(PPh_3_)_2_] (δ −1939 ppm (Δ_1/2_ = 8.2 kHz)).^43^ The ^99^Tc signal for the phenylacetylide complex **8** was not observed, likely due to a high field gradient induced by the ligands leading to a distinctly rapid quadrupolar relaxation of ^99^Tc. Thus, the signal is broadened to the point of becoming unrecognizable on the NMR time scale. The ν_C≡C_ is found at 2070 (Tc–C≡C–Ph) and 2010 cm^–1^ (Tc–C≡C–TMS), respectively. The two respective bands for the CO ligands appeared at 1915 and 1841 (Tc–C≡C–Ph) or 1844 (Tc–C≡C–TMS) cm^–1^ in 1:1 ratios. Further structural evidence for the nature of the acetylide complexes was found in a single-crystal structure analysis by XRD. Suitable crystals of **9** were grown from a saturated pentane solution at −20 °C (Figure 5).

The intercarbonyl angle (C24–Tc1–C25) is 88.9(2)° comparable to **4** and **6**. The carbon–carbon triple bond length is 1.211(7) Å, similar to C_2_H_2_ (1.20(2) Å^44^ and [^99^Tc^I^(−C≡C–TMS)(CO)_3_(PPh_3_)_2_] (1.213(6) Å).^43^ Bond lengths and angles for the PNP ligand remain nearly unchanged compared to **4**. We note that alkynes must be terminal, bearing an “acidic” proton (the p*K*_a_ of phenylacetylene is 28.7 in DMSO^45^). Diphenylacetylene did not react with **4**, confirming the observation. Terminal alkenes (such as 1-hexene) did not react with **4** either, presumably due to the far higher p*K*_a_ of alkenes (ca. 44 for, e.g., propene^45^).

### In Situ Formation of a Carbene Pincer Complex

The reactivity of **4** with acetylenes demonstrated its capability to act as an internal base in interaction with mildly acidic substrates. In line with the terminal alkynes, we aimed at the formation of a carbene complex upon in situ deprotonation of an imidazolium cation. Adding dimethyl imidazolium triflate [DMIM](OTf) to a solution of **4** in pentane at −20 °C, the color rapidly turned yellow, and a precipitate formed. The product was extracted with benzene and filtered, and the carbene complex *cis*-[^99^Tc^I^(^Pyr^PNP^*t*Bu^)(CO)_2_(DMIM)] was obtained after evaporation of solvent (**10**, Scheme 3).

The spectroscopic analysis of the carbene complex indicated a coordinated NHC carbene unit, hindered in its axial rotation due to the steric demands of the −P(^*t*^Bu)_2_ groups. The methine in **4** deprotonated the [DMIM] cation (p*K*_a_ of 23.0 in water),^46^ thereby forming the NHC-carbene in situ. The carbene unit is virtually “locked in” between the phosphine substituents in contrast to the acetylides in **8** and **9**, which are rotating freely. The ^1^H NMR spectrum reflects this structural feature in the distinctly different chemical shifts of the methyl and methylene protons. The two CH_3_ groups are observed as two singlets at δ 3.72 ppm (3H, ^Im^C*H*_3_) and δ 3.37 ppm (3H, ^Im^C*H*_3_), while the two broad singlets δ 5.89 ppm (1H, ^Im^C*H*) and δ 5.73 ppm (1H, ^Im^C*H*) are assigned to the CH protons of the carbene unit. Interestingly, the protons for the pyridine moiety appear as a set of three signals at δ 6.58 ppm (*t*, ^3^*J*_HH_ = 7.37 Hz, 1H, C*H*_pyr(4)_), 6.38 ppm (d, ^3^*J*_HH_ = 8.65 Hz, 1H, C*H*_pyr(5)_), and 5.63 ppm (d, ^3^*J*_HH_ = 6.30 Hz, 1H, C*H*_pyr(3)_). Two broad signals were observed in the ^31^P{^1^H} NMR spectrum at δ 82.23 and 61.66 ppm. The ^99^Tc NMR resonance for **10** was found at δ −1277 ppm (Δ_1/2_ = 5.5 kHz). The carbon signals for the carbonyl ligands and the carbene (−N–*C*–N−) could not be observed due to coupling to ^99^Tc. However, the carbonyl ligands gave rise to two bands at 1910 and 1822 cm^–1^ (1:1 ratio) in the IR spectrum, which are clearly shifted compared to **4**.

### Five-Membered Heterocycles

Following the reaction with [DMIM](OTf), five-membered heterocycles with similar acidities, such as pyrrole (p*K*_a_ ≈ 23.0),^45^ reacted with **4**. Following the same protocol as above, the color of the solution changed from green to yellow after 10 min. Work-up gave the pyrrolide complex *cis*-[^99^Tc^I^(^Pyr^PNP^*t*Bu^)(CO)_2_(NC_4_H_4_)] (**11**, Scheme 3) as a yellow solid. Pyrazole (p*K*_a_ ≈ 19.8),^45^ slightly more acidic than pyrrole, reacted analogously, and the pyrazolide complex *cis*-[^99^Tc^I^(^Pyr^PNP^*t*Bu^)(CO)_2_(N_2_C_3_H_3_)] (**12**, Scheme 3) was obtained as a yellow solid. The comparable structures of both complexes were confirmed with spectroscopy and XRD studies.

**Figure 6:**
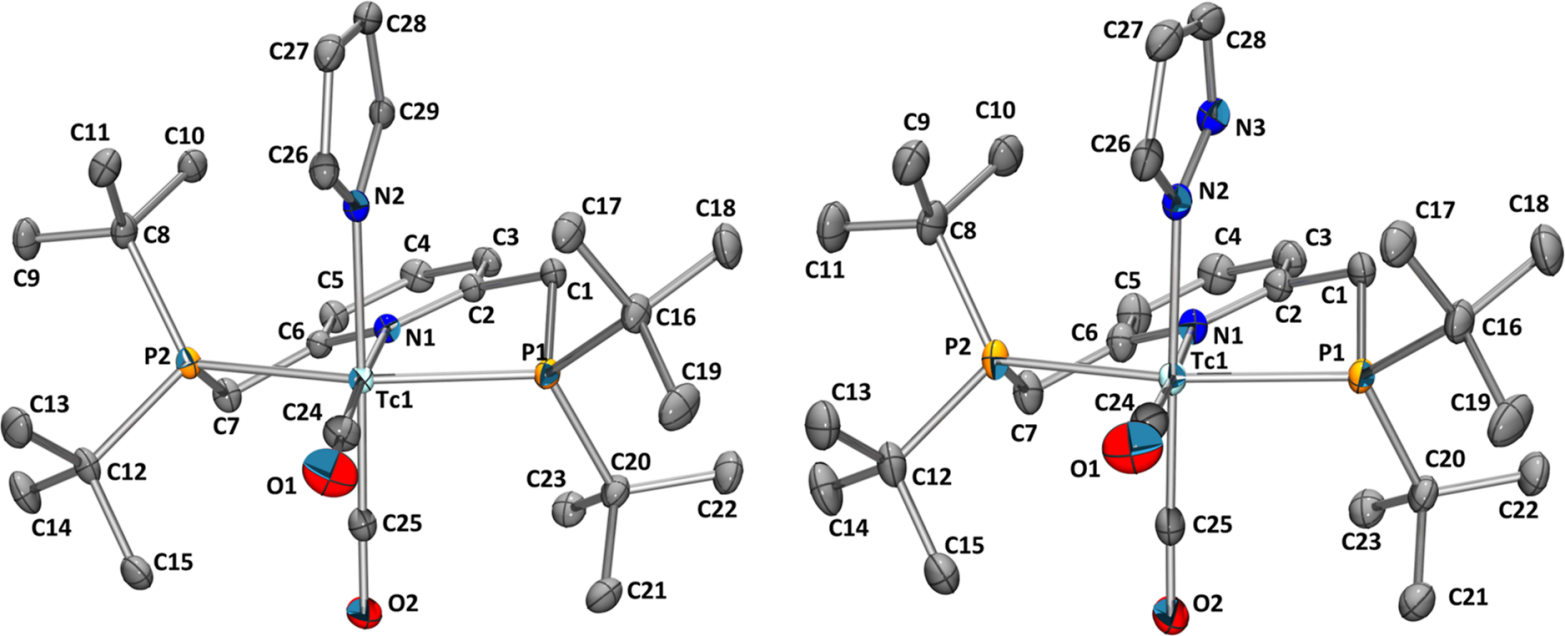
Ellipsoid replacement plots^37^ of *cis*-[^99^Tc^I^(^Pyr^PNP^*t*Bu^)(CO)_2_(NC_4_H_4_)] (**11**, left) and *cis*-[^99^Tc^I^(^Pyr^PNP^*t*Bu^)(CO)_2_(N_2_C_3_H_3_)] (**12**, right). Ellipsoids represent a 35% probability. Hydrogen atoms are omitted for clarity.

As was found for **10**, the ^1^H NMR spectra of **11** and **12** evidenced coordination of the ligands and hindered rotation due to being “sandwiched” between the −P(^*t*^Bu)_2_ groups. The pyrrolide protons appear as four distinct signals at δ 7.69 ppm (1H, C*H*_(26)_), 6.69 ppm (1H, C*H*_(27)_), 6.60 ppm (1H, C*H*_(28)_), and 6.10 ppm (1H, C*H*_(29)_). This asymmetry and the large down-field shift of the C*H*_(26)_ proton is a result of its proximity to the aromatic pyridine ring of the PNP ligand and the influence of the aromatic ring current (cf., solid-state structure in Figure 6). The 2D and correlation NMR spectra confirm the coordination of the pyrrolide. The protons of the PNP ligand appeared in comparable regions as for the other complexes in this study. The phosphorus resonances were observed as a broadened multiplet at δ 79.44 ppm in the ^31^P{^1^H} NMR spectrum (Δ_1/2_ = 3.7 kHz) while the ^99^Tc signal was found at δ −1000 ppm (Δ_1/2_ = 4.0 kHz). NMR spectra of the pyrazolide complex **12** are comparable to those of the nearly identical pyrrolide compound (Figures S67–S73 and S79–S85). Three protons for the pyrazolide moiety were observed at 8.25 ppm (1H, C*H*_(28)_), 7.83 ppm (1H, C*H*_(27)_), and 6.50 ppm (1H, C*H*_(26)_) in the ^1^H NMR spectrum. The pyrazolide ligand is also hindered in its rotation. Interestingly, the observation of a single set of three protons clearly indicates that the anionic ligand selectively coordinates with the imine oriented in the direction of the pyridine ring. This strongly suggests that the reaction proceeds either via a concerted deprotonation-coordination pathway or a stepwise pathway via imine coordination and subsequent deprotonation. The ^31^P(1H) and ^99^Tc NMR resonances of **12** appear at virtually the same shifts compared to **11** at δ 76.95 ppm (Δ_1/2_ = 3.8 kHz) and δ −1013 ppm (Δ_1/2_ = 4.0 kHz), respectively.

The structural similarity of **11** and **12** is confirmed by X-ray structure analyses. For example, both anionic ligands are bound to Tc with an approximately identical bond length (Tc1–N2) of 2.222(3) Å (**11**) and 2.2204(15) Å (**12**). The two structures reveal distorted octahedral geometries with bite angles defined by the PNP ligand (P1–Tc1–P2) of 158.06(4) Å for **11** and 157.359(17) Å for **12**, respectively. The carbonyl ligands are *cis* oriented (C24–Tc1–C25: 86.67(17)° for **11**, 89.61(9)° for **12**), substantiating the 1:1 ratio of the carbonyl bands found in the IR spectrum, comparable to all other cis compounds in this work. Unsurprisingly, the general coordination geometry of the PNP ligand was determined to be relatively similar to that of the other discussed compounds. The solid-state structure further verifies that the rotation of the pyrrolide and pyrazolide ligands is rendered impossible due to the bulky ^*t*^Bu groups. Especially in the pyrrolide complex **11**, this leads to the largely differing chemical shifts of the four protons as they are governed by the relative surroundings of the pincer ligand. Due to the constraints of the phosphine substituents and hindered rotation of the heterocyclic anionic ligands, the proton signals are not observed with their expected multiplicities but rather as broadened singlets. Pyrrolide and pyrazolide complexes **11** and **12** with ^99^Tc are the first of their kind. Only two pyrrolide complexes have been reported for Re^47,48^ and one pyrazolide complex for Mn within the manganese triad.^49^

## Conclusions

The well-established activation of substrates via MLC with rhenium pincer complexes was translated to its lighter homologue, technetium. Reactions with H_2_ and CO_2_ substantiated equivalent reactivities of Re and ^99^Tc in low oxidation states. We emphasize that this is not a necessity given the similarity between 4d and 5d elements, as distinct differences have been reported in the literature.^41,42^ The concept of MLC was applied to an extended substrate scope with a focus on weakly acidic ligands. The dearomatized pincer backbone of *cis*-[^99^Tc^I^(^Pyr^PNP^*t*Bu^*)(CO)_2_] acts as the internal base in reactions with H_2_, CO_2_, terminal alkynes, an imidazolium cation, pyrrole, and pyrazole. The driving force for these reactions is the rearomatization of the highly basic (Lewis and Brønsted) pincer ligand, formally stabilized by the technetium center. The formation of ^99^Tc acetylide complexes without initial deprotonation is the first of its kind for Tc. The fact that less acidic substrates such as 1-hexene did not react highlights the limitations of this reactivity pathway. Based on the p*K*_a_ values of the substrates, the p*K*_a_ of the dearomatized technetium complex can thus be roughly estimated as being between 28.7 and 32.2 (p*K*_a_ of ^*t*^BuOH in DMSO: 32.2).^50^ This work contributes to the closing of a “knowledge gap” between Re and Mn.

## Supporting Information Available

General experimental details, procedures, and data, spectra, and crystallographic data (PDF)
