# Supplementary material for: Binding Small Molecules to a cis-Dicarbonyl 99TcI-PNP Complex via Metal–Ligand Cooperativity
Source: Inorg Chem. 2023 Jun 23;62(27):10727–35. doi: 10.1021/acs.inorgchem.3c01177 (PMC10336964; doi:10.1021/acs.inorgchem.3c01177)
Supplement: Supplementary file 1 — ic3c01177_si_001.pdf [file ic3c01177_si_001.pdf]

Supporting Information

Binding small molecules to a *cis*-dicarbonyl $^{99}Tc^{I}$-PNP complex via metal-ligand cooperativity

Manuel Luca Besmer,[a] Henrik Braband,[a] Thomas Fox,[a] Bernhard Spingler,[a] Alfred P. Sattelberger,[b] and Roger Alberto* [a]

[a] Department of Chemistry, University of Zurich, Winterthurerstrasse 190, CH-8057 Zurich, Switzerland

[b] Department of Chemistry, University of Central Florida, 4111 Libra Drive, Orlando, FL 32816, United States

E-mail: ariel@chem.uzh.ch

Table of Contents

1 General Experimental Details 3

2. Remarks on NMR spectroscopy with ^{99}Tc complexes 3

3 Experimental Procedures and Data 5

3.1 [Tc(^{Pyr}PNP^{tBu})(CO)$_2$Cl] (3) 5

3.2 [Tc(^{Pyr}PNP^{tBu*})(CO)$_2$] (4) 5

3.3 [Tc(^{Pyr}PNP^{tBu}–COO)(CO)$_2$] (5) 6

3.4 [Tc(^{Pyr}PNP^{tBu}–CSS)(CO)$_2$] (6) 7

3.5 [Tc(^{Pyr}PNP^{tBu})(CO)$_2$H] (7) 7

3.6 [Tc(^{Pyr}PNP^{tBu})(CO)$_2$(–C≡C–Ph)] (8) 8

3.7 [Tc(^{Pyr}PNP^{tBu})(CO)$_2$(–C≡C–SiMe$_3$)] (9) 9

3.8 [Tc(^{Pyr}PNP^{tBu})(CO)$_2$(DMIM)] (10) 10

3.9 [Tc(^{Pyr}PNP^{tBu})(CO)$_2$(NC$_4$H$_4$)] (11) 11

3.10 [Tc(^{Pyr}PNP^{tBu})(CO)$_2$(N$_2$C$_3$H$_3$)] (12) 12

4 Spectra 13

4.1 [Tc(^{Pyr}PNP^{tBu})(CO)$_2$Cl] (3) 13

4.2 [Tc(^{Pyr}PNP^{tBu*})(CO)$_2$] (4) 14

4.3 [Tc(^{Pyr}PNP^{tBu}–COO)(CO)$_2$] (5) 19

4.4 [Tc(^{Pyr}PNP^{tBu}–CSS)(CO)$_2$] (6) 23

4.5 [Tc(^{Pyr}PNP^{tBu})(CO)$_2$H] (7) 28

4.6 [Tc(^{Pyr}PNP^{tBu})(CO)$_2$(–C≡C–Ph)] (8) 32

4.7 [Tc(^{Pyr}PNP^{tBu})(CO)$_2$(–C≡C–TMS)] (9) 36

4.8 [Tc(^{Pyr}PNP^{tBu})(CO)$_2$(DMIM)] (10) 41

4.9 [Tc(^{Pyr}PNP^{tBu})(CO)$_2$(NC$_4$H$_4$)] (11) 46

4.10 [Tc(^{Pyr}PNP^{tBu})(CO)$_2$(N$_2$C$_3$H$_3$)] (12) 52

5 Crystallographic Data 57
5.1 [Tc(^{Pyr}PNP^{tBu})Cl(CO)$_2$] (3) 57
5.2 [Tc(^{Pyr}PNP^{tBu*})(CO)$_2$] (4) 59
5.3 [Tc(^{Pyr}PNP^{tBu}–CSS)(CO)$_2$] (6) 61
5.4 [Tc(^{Pyr}PNP^{tBu})(CO)$_2$(–C≡C–SiMe$_3$)] (9) 63
5.5 [Tc(^{Pyr}PNP^{tBu})(CO)$_2$(NC$_4$H$_4$)] (11) 65
5.6 [Tc(^{Pyr}PNP^{tBu})(CO)$_2$(N$_2$C$_3$H$_3$)] (12) 67
6. References 69

1 General Experimental Details

Materials: Unless otherwise stated, all chemicals were of reagent grade quality or higher, obtained from commercial sources and used without further purification. Solvents for reactions were of *p.a.* grade or distilled prior to their use. Deuterated NMR-solvents were purchased from *Armar Chemicals* (CH) or *Cambridge Isotope Laboratories, Inc.* (UK). $(NH_4)[^{99}TcO_4]$ was purchased from *Oak Ridge* and treated with H_2O_2 prior to reactions for re-oxidation of black, surface TcO_2.[1] Complex [**1**] ($Tc(^{Pyr}PNP^{tBu})Cl_3$) and 1,3-dimethylimidazolium trifluoromethanesulfonate [DMIM](OTf) were prepared according to literature synthesis.[2,3] All reactions were carried out in an inert atmosphere (nitrogen) glove-box (*MBraun* LABmaster DP). Caution: ^{99}Tc is a weak β-emitter. All experiments must be carried out in licensed and appropriately shielded laboratories for low-level radioactive materials. **NMR:** NMR spectra were recorded in C_6D_6 at 298 K on *Bruker AV-400* (400 MHz) or *Bruker AV2-500* (500 MHz) spectrometers. Sensitive samples were measured in *Young* valve-closed NMR tubes. ^{1}H and ^{13}C chemical shifts (δ) are given in ppm relative to residual solvent resonances (C_6D_5H ^{1}H: δ 7.16; ^{13}C: δ 128.39). Signal assignments are based on coupling constants, increment calculations and/or supportive NMR experiments. The NMR measurements were highly influenced by the characteristics of technetium. Its paramagnetic nature and the large nuclear quadrupolar moment both strongly accelerate t_1 and t_2 relaxation up to complete signal extinction. The values given for the ^{99}Tc chemical shifts are referenced to pertechnetate. **IR:** FT-IR spectra were recorded with *SpectrumTwo FT-IR Spectrometer* (*Perkin–Elmer*) and samples were run as KBr pellets. **Visible absorption spectroscopy:** *Compact Spectrometer CCS200/M (THORLABS);* lamp: *SLS201/M (THORLABS)*. **LSC: Technetium content measurements:** Products were dissolved in the appropriate solvents. The measurements were carried out with a scintillation cocktail (Packard Ultimate Gold XR) and a *Hidex 300 SL* liquid scintillation counter. Yields of compounds were calculated from activity yields by LSC measurement. **X-ray diffraction:** Single-crystal X-ray diffraction data was collected at 160(1) K on a *Rigaku OD XtaLAB Synergy*, Dualflex, *Pilatus 200K* diffractometer using a single wavelength X-ray source (Cu K_α radiation: λ = 1.54184 Å for (**3**, **4**, **6**); or Mo K_α radiation: l = 0.71073 Å for **11**) or on a *Rigaku XtaLAB Synergy*, Dualflex, *HyPix* diffractometer using a single wavelength X-ray source (Cu K_α radiation: λ = 1.54184 Å) (**9**, **12**) from a micro-focus sealed X-ray tube and an *Oxford* liquid-nitrogen *Cryostream* cooler. The selected suitable single crystal was mounted using polybutene oil on a flexible loop fixed on a goniometer head and immediately transferred to the diffractometer. Pre-experiments, data collection, data reduction and analytical absorption correction[4] were performed with the program suite *CrysAlis^{Pro}*.[5] Using *Olex2*,[6] the structures were solved with the *SHELXT*[7] small molecule structure solution program and refined with the *SHELXL 2018/3*[8] program package by full-matrix least-squares minimization on F^2. *PLATON*[9] was used to check the result of the X-ray analysis. For more details about the data collection and refinement parameter, see the CIF files.

2. Remarks on NMR spectroscopy with ^{99}Tc complexes

The influence of quadrupolar nuclei like ^{99}Tc (spin I = 9/2, quadrupole relaxation time T_{1Q}) onto the relaxation time of scalarly coupled nuclei like ^{13}C ($T_{1\text{-}SC}$) is described by formula (I) according to literature.[10]

$$\frac{1}{T_{1-SC}} = \frac{8\pi^2 J^2}{3} I(I+1)\frac{T_{1Q}}{1+(\omega_C-\omega_I)^2 T_{1Q}^2} \qquad \text{(I)}$$

In this regard, a significant increase of ^{13}C T_1 relaxation due to strong scalar coupling with ^{99}Tc might be an explanation for the unobservability of carbon sites which are directly bound to the technetium center (CO, acetylide, carbene ligands):

Considering ^{13}C-^{99}Tc coupling constants J of some 100 Hz and a typical ^{99}Tc relaxation time T_{1Q} in the *millisecond* range, in conjunction with the close resonance frequencies of carbon and technetium (125 vs. 113 MHz), relation (I) predicts significant scalar ^{13}C relaxation $1/T_{1\text{-}SC}$ in the kHz range, causing broadening and weakening effects onto the ^{13}C signal, up to its loss.

Rhenium bound ^{13}C nuclei are, in contrast, *not* influenced by scalar T_1 relaxation as both rhenium isotopes ($^{185,187}Re$) undergo extraordinarily fast quadrupolar relaxation T_{1Q} in the *microsecond* range. Therefore, equation (I) yields values close to zero, cancelling any scalar relaxation effects $1/T_{1\text{-}SC}$ in the case of rhenium.

3 Experimental Procedures and Data

3.1 [Tc(^{Pyr}PNP^{tBu})(CO)$_2$Cl] (3)

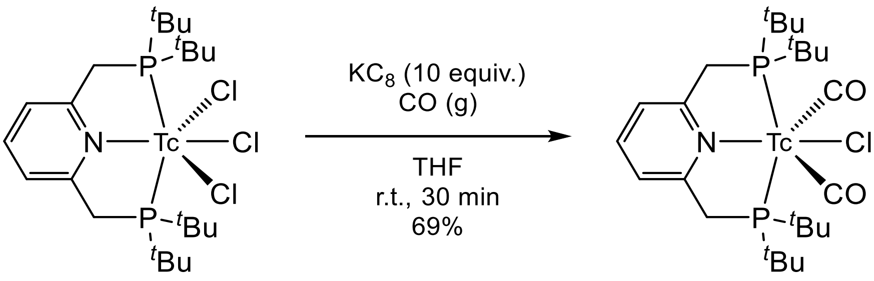

[1] [3]

Synthesis: A round-bottom flask was charged with starting material **1** (6.2 mg, 10.3 µmol), KC_8 (13.5 mg, 100 µmol) and a magnetic stir bar. The closed flask was then flushed with CO (g) by means of a syringe for 15 min. Subsequently, THF (3 mL) was added, and the suspension was stirred for 30 min during which time the color of the suspension turned intensely red. The solvent was evaporated under a stream of Ar(g). Extraction with benzene (3x3 mL) and subsequent filtration through a P3 glass filter resulted in a deep red solution. After evaporation of solvent the product **3** was afforded as red crystalline material (4.16 mg, 7.1 µmol, 69%). Red single crystals were obtained by slow evaporation of a concentrated benzene solution.

Analysis: IR (KBr) ν [cm^{-1}]: 3408*s*, 2918*m*, 2851*m*, 2024*w*, 1952*w* (CO), 1867*m* (CO), 1645*m*, 1458*m*, 1392*w*, 1370*w*, 1178*w*, 1039*w*, 1008*m*, 914*w*, 851*w*, 669*w*, 614*w*. **^{99}Tc NMR** (112.57 MHz, C_6D_6) δ [ppm]: -1266 ($\Delta_{1/2}$ = 337 Hz). **Vis** (C_6H_6) λ_{max} 413 (2808), λ_{max} 477 (885), λ_{max} 527 (959). **^{99}Tc analysis** Calc. for $C_{25}H_{43}ClNO_2P_2Tc$ (%): 18.28. Found: 16.91.

3.2 [Tc(^{Pyr}PNP^{tBu*})(CO)$_2$] (4)

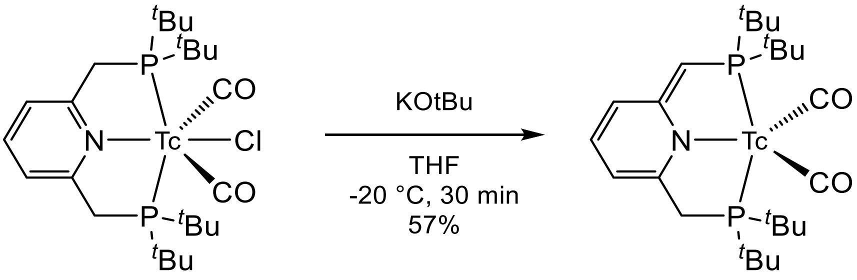

[3] [4]

Synthesis: Complex **3** (8.9 mg, 15.2 µmol) was dissolved in THF (4 mL) in a round-bottom flask and the red solution was cooled to −20 °C. The stirred solution was treated with dropwise addition of KO^{t}Bu (1 M in THF, 3 equiv.). After allowing the reaction mixture to warm, the solution was stirred at 23 °C for 30 min the color changed from red to green and the volatiles were evaporated *in vacuo*. The green residue was extracted with pentane, filtered through a syringe filter (PTFE 0.22 µm) and the solvent was evaporated at ambient pressure. Product **4** was obtained as a green solid (9 µmol, 4.8 mg) in a yield of 57% (+ 1 PPh_3). Crystals suitable for XRD were grown from a saturated pentane solution at −20 °C.

Analysis: **IR** (KBr) ν [cm^{-1}]: 3435*m*, 2956*s*, 2923*s*, 2853*m*, 1926*m* (υ_{CO}, 1:1 ratio with 1845 cm^{-1}), 1845*m* (υ_{CO}, 1:1 ratio with 1926 cm^{-1}), 1463*m*, 1377*w*, 1201*m*, 990*w*, 481*w*. **^{1}H NMR** (400 MHz, C_6D_6)

δ [ppm]: 6.38 (*m*, 2 H, C$H_{pyr(4,5)}$); 5.41 (*d*, $^{3}J_{HH}$ = 5.79 Hz, 1 H, C$H_{pyr(3)}$); 3.76 (*d*, $^{2}J_{HP}$ = 2.76 Hz, 1 H, PC*H*); 2.74 (*d*, $^{2}J_{HP}$ = 9.03 Hz, 1 H, PCH_2); 1.28 (*d*, $^{3}J_{PH}$ = 13.39 Hz, 18 H, ((CH_3)$_3$CP, overlaid with impurities); 0.98 (*d*, $^{3}J_{PH}$ = 13.16 Hz, 18 H, ((CH_3)$_3$CP, overlaid with impurities). **^{13}C{^{1}H} NMR** (125.72 MHz, C_6D_6) δ [ppm]: 173.3 (1 C, $C_{pyr(2)}$, from HMBC); 159.8 (1 C, $C_{pyr(6)}$, from HMBC); 132.0 (*s*, 1 C, $CH_{pyr(4)}$); 116.6 (*d*, $^{3}J_{PC}$ = 17.2 Hz, 1 C, $CH_{pyr(3)}$); 99.5 (*d*, $^{3}J_{PC}$ = 10.38 Hz, 1 C, $CH_{pyr(5)}$); 68.3 (*d*, $^{1}J_{PC}$ = 48.30 Hz, 1 C, P*C*H=C); 38.2 (*d*, $^{1}J_{PC}$ = 22.59 Hz, 2 C, P(*C*(CH$_3$)$_3$)$_2$); 36.1 (*d*, $^{1}J_{PC}$ = 13.81 Hz, 2 C, H$_2$C−P(*C*(CH$_3$)$_3$)$_2$); 35.7 (*d*, $^{1}J_{PC}$ = 15.31 Hz, 1 C, P*C*H$_2$); 29.6 (*d*, $^{2}J_{PC}$ = 4.73 Hz, 6 C, P(C(*C*H$_3$)$_3$)$_2$); 29.1 (*d*, $^{2}J_{PC}$ = 4.77 Hz, 6 C, P(C(*C*H$_3$)$_3$)$_2$); missing signals for 2 *C*O. **^{31}P{^{1}H} NMR** (202.46 MHz, C_6D_6) δ [ppm]: 80.44 (*m*, 1 P, *P*CH$_2$C), 70.67 (*m*, 1 P, *P*CH=C). **^{99}Tc NMR** (112.57 MHz, C_6D_6) δ [ppm]: −1103 ($\Delta_{1/2}$ = 3.4 kHz). **Vis** (THF) λ_{max} 395 (1473), λ_{max} 412 (1738), λ_{max} 616 (794). **^{99}Tc analysis** Calc. for $C_{25}H_{42}NO_2P_2Tc$ $C_{26}H_{42}NO_2P_2Tc$ (·1 $C_{18}H_{15}P$) (%): 12.11. Found: 12.62.

3.3 [Tc(^{Pyr}PNP^{tBu}–COO)(CO)$_2$] (5)

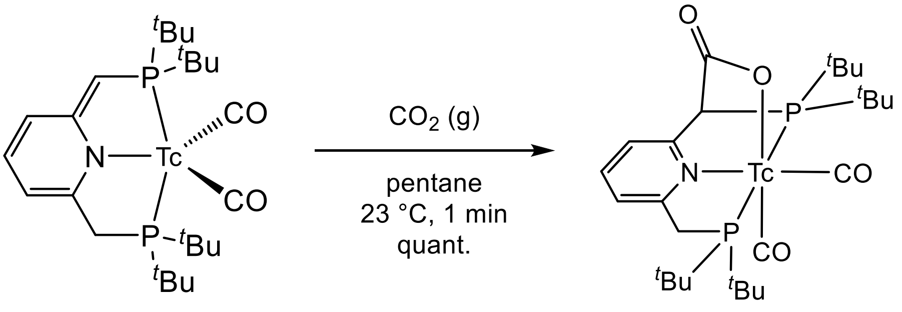

[4] [5]

Synthesis: Freshly prepared **4** (6.8 mg, 12.4 µmol) was dissolved in pentane (3 mL) in a round-bottom flask. CO_2 (g) was bubbled through the greenish solution via syringe at 23 °C and a rapid color change to pale yellow was observed. After 1 min the reaction was complete and the product precipitated. The solvent was evaporated under a stream of Ar to give **5** in quantitative yield as a pale-yellow residue.

Analysis: **IR** (KBr) ν [cm^{-1}]: 3440*m*, 2962*m*, 2923*m*, 2870*m*, 2853*m*, 1929*s* (υ_{CO}, 1:1 ratio with 1848 cm^{-1}), 1848*s* (υ_{CO}, 1:1 ratio with 1929 cm^{-1}), 1648*s*, 1459*s*, 1434*s*, 1394*s*, 1323*m*, 1176*m*, 1022*m*, 877*w*, 836*w*, 696*w*, 630*w*. **^{1}H NMR** (500 MHz, C_6D_6) δ [ppm]: 6.78 (*t*, $^{3}J_{HH}$ = 7.78 Hz, 1 H, C$H_{pyr(4)}$); 6.73 (*d*, $^{3}J_{HH}$ = 7.76 Hz, 1 H, CH_{pyr}); 6.34 (*d*, $^{3}J_{HH}$ = 7.70 Hz, 1 H, CH_{pyr}); 4.61 (*dd*, $^{2}J_{HP}$ = 6.68 Hz, $^{4}J_{HP}$ = 1.55 Hz, 1 H, PC*H*); 2.93 (*dd*, $^{2}J_{HH}$ = 15.92 Hz, $^{2}J_{HP}$ = 4.37 Hz, 1 H, PCH_2); 2.83 (*dd*, $^{2}J_{HH}$ = 16.10 Hz, $^{2}J_{HP}$ = 9.54 Hz, 1 H, PCH_2); 1.57 (*d*, $^{3}J_{HP}$ = 12.61 Hz, 9 H, (CH_3)$_3$CP); 1.31 (*d*, $^{3}J_{HP}$ = 13.25 Hz, 9 H, (CH_3)$_3$CP overlaid with impurities); 0.94 (*d*, $^{3}J_{HP}$ = 12.07 Hz, 9 H, (CH_3)$_3$CP overlaid with impurities); 0.80 (*d*, $^{3}J_{HP}$ = 12.50 Hz, 9 H, (CH_3)$_3$CP). **^{13}C{^{1}H} NMR** (125.72 MHz, C_6D_6) δ [ppm]: 170.4 (*dd*, $^{2}J_{CP}$ = 10.11 Hz, $^{4}J_{CP}$ = 1.74 Hz, 1 C, Tc−O−*C*=O); 162.7 (*d*, $^{2}J_{PC}$ = 4.87 Hz, 1 C, C_{pyr}); 162.5 (*t*, $^{2}J_{PC}$ = 3.65 Hz, 1 C, C_{pyr}); 137.8 (*s*, 1 C, $CH_{pyr(4)}$); 119.7 (*d*, $^{3}J_{PC}$ = 5.99 Hz, 1 C, CH_{pyr}); 119.4 (*d*, $^{3}J_{PC}$ = 6.75 Hz, 1 C, CH_{pyr}); 61.8 (*d*, $^{1}J_{CP}$ = 4.94 Hz, 1 C, P*C*H−COO); 37.48 (*d*, $^{1}J_{CP}$ = 13.36 Hz, 1 C, P*C*H$_2$); 37.2 (*m*, overlay 4 C, P(*C*(CH$_3$)$_3$)); 30.9 (*d*, $^{2}J_{CP}$ = 4.50 Hz, 3 C, P(C(*C*H$_3$)$_3$)$_2$); 30.5 (*d*, $^{2}J_{CP}$ = 4.06 Hz, 3 C, P(C(*C*H$_3$)$_3$)$_2$); 30.1 (*d*, $^{2}J_{CP}$ = 3.52 Hz, 3 C, P(C(*C*H$_3$)$_3$)$_2$); 29.7 (*d*, $^{2}J_{CP}$ = 4.18 Hz, 3 C, P(C(*C*H$_3$)$_3$)$_2$); missing signals for 2 *C*O. **^{31}P{^{1}H} NMR** (202.46 MHz, C_6D_6) δ [ppm]: 113.2 (broad *m*, 1 P), 91.1 (broad *s*, 1 P). **^{99}Tc NMR** (112.57 MHz, C_6D_6) δ [ppm]: −1061 ($\Delta_{1/2}$ = 2.3 kHz). **Vis** (C_6H_6) λ_{max} 397 (397), λ_{sh} 521 (128). **^{99}Tc analysis** Calc. for $C_{26}H_{42}NO_2P_2Tc$ (·0.15 $C_{18}H_{15}P$) (%): 15.63. Found: 13.05.

3.4 [Tc(^{Pyr}PNP^{tBu}–CSS)(CO)$_2$] (6)

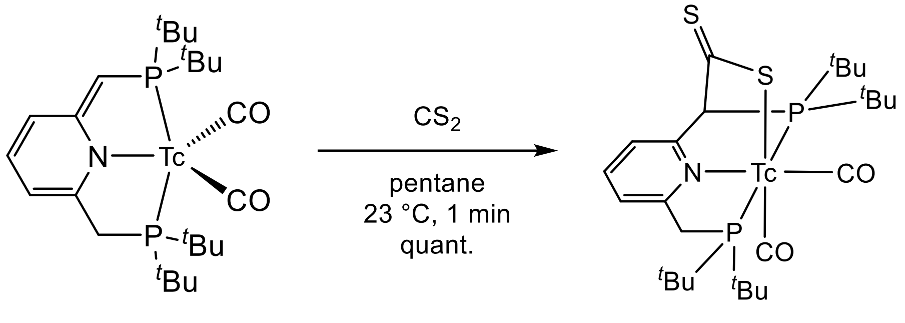

[**4**] [**6**]

Synthesis: Freshly prepared **4** (4.1 mg, 7.4 µmol) was dissolved in pentane (3 mL) in a round-bottom flask and cooled to -20 °C. A few drops of CS_2 were added to the cooled, stirred solution upon which a upon which a rapid color change to dark yellow and precipitation was observed. After 1 min the reaction was complete, and the solvent was slowly evaporated. Extraction of the residue with C_6H_6 and subsequent filtration yielded **6** (+ 1.35 PPh_3) quantitatively as a dark yellow residue. Crystals of **6** (· PPh3) suitable for XRD were grown from a saturated toluene solution at −20 °C.

Analysis: **IR** (KBr) ν [cm^{-1}]: 3434*w*, 2953*m*, 2925*s*, 2853*m*, 1934*s* (υ_{CO}, 1:1 ratio with 1872 cm^{-1}), 1872*s* (υ_{CO}, 1:1 ratio with 1934 cm^{-1}), 1584*w*, 1460*m*, 1434*w*, 1373*w*, 1262*w*, 1174*w*, 1090*w*, 1040*w*, 1021*m*, 1006*m*, 801*w*, 742*w*, 696*w*, 640*w*, 610*w*. **^{1}H NMR** (500 MHz, C_6D_6) δ [ppm]: 6.99 (*d*, $^3J_{HH}$ = 7.95 Hz, 1 H, −CPH−C−CH_{pyr}); 6.71 (*t*, $^3J_{HH}$ = 7.75 Hz, 1 H, C$H_{pyr(4)}$); 6.29 (*d*, $^3J_{HH}$ = 7.70 Hz, 1 H, −CPH$_2$−C−CH_{pyr}); 5.89 (*dd*, $^2J_{HP}$ = 6.63 Hz, $^4J_{HP}$ = 1.48 Hz, 1 H, PC*H*); 2.87 (*dd*, $^2J_{HH}$ = 15.86 Hz, $^2J_{HP}$ = 4.90 Hz, 1 H, PCH_2); 2.79 (*dd*, $^2J_{HH}$ = 15.88 Hz, $^2J_{HP}$ = 9.48 Hz, 1 H, PCH_2); 1.67 (*d*, $^3J_{HP}$ = 12.71 Hz, 9 H, (CH_3)$_3$CP); 1.29 (*d*, $^3J_{HP}$ = 13.11 Hz, 18 H, (CH_3)$_3$CP overlaid with impurities); 0.93 (*d*, $^3J_{HP}$ = 11.16 Hz, 9 H, (CH_3)$_3$CP). **^{13}C{^{1}H} NMR** (125.72 MHz, C_6D_6) δ [ppm]: 162.0 (*dd*, $^2J_{PC}$ = 5.08 Hz, $^4J_{PC}$ = 2.47 Hz, 1 C, −CPH$_2$−C_{pyr}); 161.0 (*m*, 2 C overlapping, Tc−S−*C*=S and C_{pyr}−CPH−CSS); 137.3 (*s*, 1 C, $CH_{pyr(4)}$); 119.5 (*d*, $^3J_{CP}$ = 6.10 Hz, 1 C, −CPH−C−CH_{pyr}); 119.4 (*d*, $^3J_{CP}$ = 7.11 Hz, 1 C, −CPH$_2$−C−CH_{pyr}); 84.4 (*d*, $^1J_{CP}$ = 8.28 Hz, 1 C, P*C*H−CSS); 38.7 (*d*, $^1J_{CP}$ = 13.28 Hz, 1 C, PCH_2); 38.1 (*d*, $^1J_{CP}$ = 5.42 Hz, 1 C, P(*C*(CH$_3$)$_3$)); 38.0 (*d*, $^1J_{CP}$ = 6.98 Hz, 1 C, P(*C*(CH$_3$)$_3$)); 37.5 (*d*, $^1J_{CP}$ = 4.30 Hz, 2 C, P(*C*(CH$_3$)$_3$)); 31.9 (*d*, $^2J_{CP}$ = 3.76 Hz, 3 C, P(C(CH_3)$_3$)$_2$); 30.5 (*d*, $^2J_{CP}$ = 4.30 Hz, 9 C, P(C(CH_3)$_3$)$_2$); missing signals for 2 *C*O. **^{31}P{^{1}H} NMR** (202.46 MHz, C_6D_6) δ [ppm]: 135.9 (broad *m*, 1 P); 88.9 (broad *m*, 1 P). **^{99}Tc NMR** (112.57 MHz, C_6D_6) δ [ppm]: –1282 ($\Delta_{1/2}$ = 1.9 kHz). **Vis** (C_6H_6) λ_{max} 428 (4317), λ_{max} 567 (525), λ_{max} 893 (97). **^{99}Tc analysis** Calc. for $C_{26}H_{42}NO_2P_2S_2Tc$ (·1.35 $C_{18}H_{15}P$) (%): 10.10. Found: 8.66.

3.5 [Tc(^{Pyr}PNP^{tBu})(CO)$_2$H] (7)

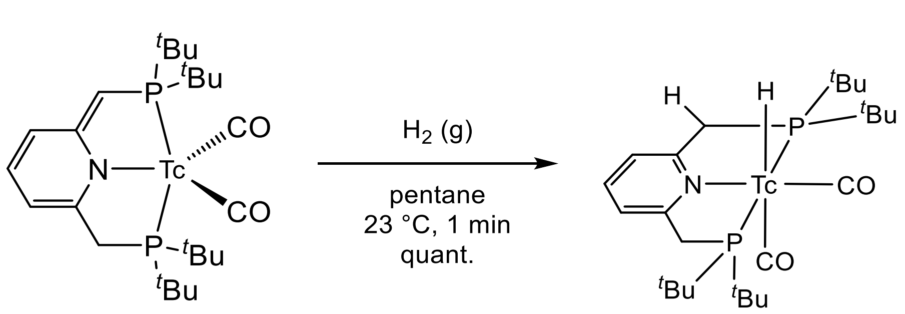

[**4**] [**7**]

Synthesis: Freshly prepared **4** (5.4 mg, 9.8 µmol) was dissolved in C_6H_6 (1 mL) in a round-bottom flask. H_2 (g) was bubbled through the greenish solution at 23 °C and a rapid color change to yellow was observed. After 1 min the reaction was complete, and the solvent was evaporated under a stream of Ar to give **7** in quantitative yield (+ 1 PPh_3) as a yellow residue.

Analysis: **IR** (KBr) ν [cm^{-1}]: 3434*m*, 2956*m*, 2921*m*, 2864*m*, 1890*s* (υ_{CO}, 1:1 ratio with 1831 cm^{-1}), 1831*s* (υ_{CO}, 1:1 ratio with 1890 cm^{-1}), 1760*w*, 1638*w*, 1564*w*, 1476*w*, 1458*m*, 1434*m*, 1387*w*, 1366*w*, 1277*w*, 1181*w*, 1020*w*, 836*w*, 812*w*, 742*w*, 696*w*, 625*w*, 481*w*. **^{1}H NMR** (500 MHz, C_6D_6) δ [ppm]: 6.68 (*t*, $^3J_{HH}$ = 7.68 Hz, 1 H, C$H_{pyr(4)}$); 6.37 (*d*, $^3J_{HH}$ = 7.70 Hz, 2 H, C$H_{pyr(3,5)}$); 3.03 (*s*, 4 H, PCH_2); 1.35 (*t*, $^3J_{HP}$ = 6.28 Hz, 18 H, (CH_3)$_3$CP overlaid with impurities); 1.29 (*t*, $^3J_{HP}$ = 6.25 Hz, 18 H, (CH_3)$_3$CP overlaid with impurities); −3.20 (*t*, $^2J_{HP}$ = 24.09 Hz, 1 H, Tc−H). **^{13}C{^{1}H} NMR** (125.72 MHz, C_6D_6) δ [ppm]: 163.5 (*m*, 2 C, C_{pyr}); 134.5 (*s*, 1 C, $CH_{pyr(4)}$); 119.19 (*t*, $^3J_{PC}$ = 3.94 Hz, 2 C, $CH_{pyr(3,5)}$); 39.8 (*t*, $^1J_{CP}$ = 5.42 Hz, 2 C, PCH$_2$); 37.1 (*t*, $^1J_{CP}$ = 5.2 Hz, 2 C, P(C(CH$_3$)$_3$)); 35.1 (*t*, $^1J_{CP}$ = 7.74 Hz, 2 C, P(C(CH$_3$)$_3$)); 30.1 (*t*, $^2J_{CP}$ = 3.07 Hz, 6 C, P(C(CH$_3$)$_3$)$_2$); 30.0 (*t*, $^2J_{CP}$ = 2.75 Hz, 6 C, P(C(CH$_3$)$_3$)$_2$); missing signals for 2 *C*O. **^{31}P{^{1}H} NMR** (202.46 MHz, C_6D_6) δ [ppm]: 103.2 (broad *m*, 2 P). **^{99}Tc NMR** (112.57 MHz, C_6D_6) δ [ppm]: –1097 ($\Delta_{1/2}$ = 2.7 kHz). **Vis** (C_6H_6) λ_{sh} 420 (245), λ_{max} 436 (265), λ_{max} 685 (21). **^{99}Tc analysis** Calc. for $C_{25}H_{44}NO_2P_2Tc$ (·1 $C_{18}H_{15}P$) (%): 12.72. Found: 10.86.

3.6 [Tc(^{Pyr}PNP^{tBu})(CO)$_2$(–C≡C–Ph)] (8)

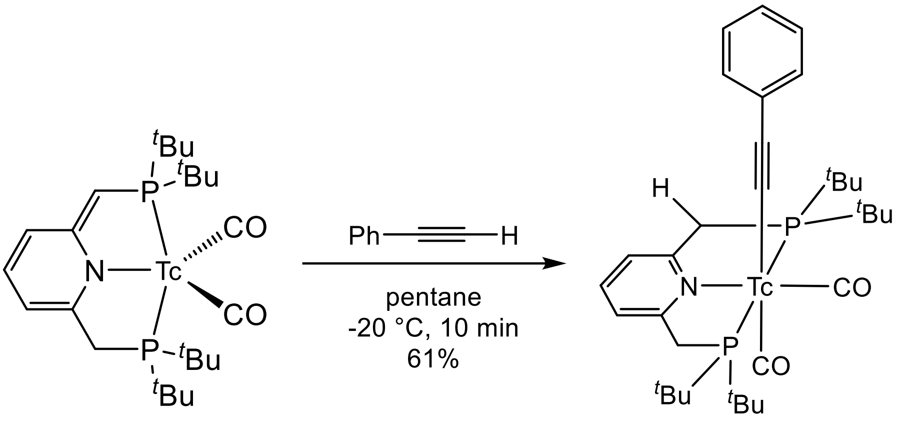

[**4**] [**8**]

Synthesis: Freshly prepared **4** (2.2 mg, 4.0 µmol) was dissolved in pentane (2 mL) in a round-bottom flask and cooled to -20 °C. Subsequently, phenylacetylene (8 equiv. as a solution in 1 mL of C_6H_6) was added to the stirred solution and let warm to room temperature. After 10 min the color of the solution had changed to slightly orangish and the volatiles were removed *in vacuo*. The product was extracted with C_6H_6, filtered and the solvent was slowly evaporated. The product **8** was obtained as a yellow/orange solid in a yield of 61 % (+ 0.38 PPh_3).

Analysis: **IR** (KBr) ν [cm^{-1}]: 3435*m*, 3070*w*, 3056*w*, 2955*s*, 2921*s*, 2852*s*, 2070*w* (C≡C), 1915*s* (υ_{CO}, 1:1 ratio with 1841 cm^{-1}), 1841*s* (υ_{CO}, 1:1 ratio with 1915 cm^{-1}), 1594*w*, 1480*m*, 1458*m*, 1368*m*, 1204*m*, 1177*m*, 1024*m*, 1000*m*, 961*w*, 841*w*, 823*w*, 754*w*, 693*w*, 628*w*, 482*w*. **^{1}H NMR** (500 MHz, C_6D_6) δ [ppm]: 7.40 (overlaid *d*, 2 H, CH_{phenyl}); 7.11 (*t*, $^3J_{HH}$ = 7.68 Hz, 2 H, CH_{phenyl}); 6.94 (*t*, $^3J_{HH}$ = 7.38 Hz, 1 H, C$H_{phenyl(4)}$); 6.76 (*t*, $^3J_{HH}$ = 7.65 Hz, 1 H, C$H_{pyr(4)}$); 6.45 (*d*, $^3J_{HH}$ = 7.65 Hz, 2 H, C$H_{pyr(3,5)}$); 3.89 (*d*, $^2J_{HH}$ = 15.36 Hz, 2 H, PCH_2); 3.06 (*d*, $^2J_{HH}$ = 15.36 Hz, 2 H, PCH_2); 1.45 (*t*, $^3J_{PH}$ = 6.18 Hz, 18 H, (PC(CH_3)$_3$); 1.25 (*t*, $^3J_{PH}$ = 5.90 Hz, 18 H, (PC(CH_3)$_3$). **^{13}C{^{1}H} NMR** (125.72 MHz, C_6D_6) δ [ppm]: 163.4 (*t*, $^2J_{PC}$ = 3.84 Hz, 2 C, $C_{pyr(2,6)}$); 135.7 (*s*, 1 C, $CH_{pyr(4)}$); 130.3 (*s*, 1 C, C_{phenyl}−C≡C−Tc); 130.1 (*s*,

2 C, CH_{phenyl}); 127.7 (*s*, 2 C, CH_{phenyl}, overlaid by solvent); 123.7 (*s*, 1 C, CH_{phenyl}); 119.6 (*t*, $^3J_{PC}$ = 3.75 Hz, 2 C, $CH_{pyr(3,5)}$); 117.0 (*s*, 1 C, −*C*≡C−Tc); 39.1 (*t*, $^1J_{PC}$ = 5.52 Hz, 2 C, P*C*H_2); 37.3 (*t*, $^1J_{PC}$ = 6.42 Hz, 2 C, P(*C*$(CH_3)_3)_2$); 37.0 (*t*, $^1J_{PC}$ = 5.11 Hz, 2 C, P(*C*$(CH_3)_3)_2$); 30.9 (*t*-like *s*, 6 C, P(C(*C*$H_3)_3)_2$); 30.2 (*t*-like *s*, 6 C, P(C(*C*$H_3)_3)_2$); missing signals for 2 *C*O and Tc−*C*≡C−. **^{31}P{^{1}H} NMR** (202.46 MHz, C_6D_6) δ [ppm]: 91.91 (broad *s*, 2 P). **^{99}Tc NMR** (112.57 MHz, C_6D_6) δ [ppm]: not observed. **Vis** (C_6H_6) λ_{max} 407 (598), λ_{sh} 514 (239), λ_{max} 705 (148), λ_{max} 791 (137). **^{99}Tc analysis** Calc. for $C_{33}H_{48}NO_2P_2Tc$ (·0.38 $C_{18}H_{15}P$) (%): 13.17. Found: 12.12.

3.7 [Tc(^{Pyr}PNP^{tBu})(CO)$_2$(–C≡C–SiMe$_3$)] (9)

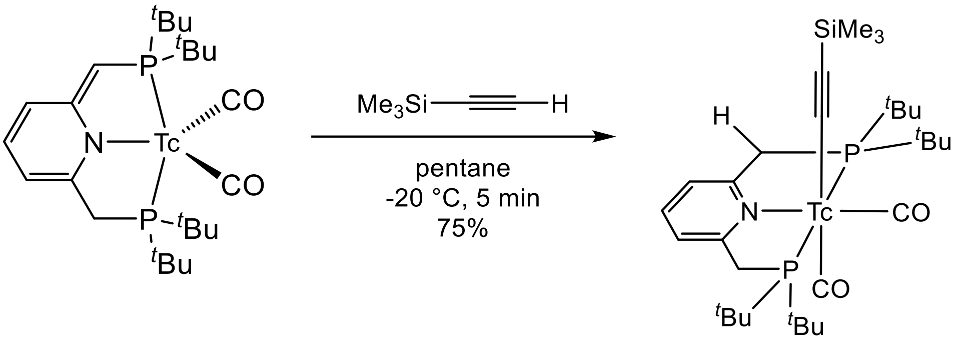

[**4**] [**9**]

Synthesis: Freshly prepared **4** (4.4 mg, 8.0 µmol) was dissolved in pentane (3 mL) in a round-bottom flask and cooled to -20 °C. Subsequently, a drop of trimethylsilylacetylene was added to the stirred solution with an *Eppendorf* pipette and left to warm to room temperature. After 5 min the color of the solution had changed to yellowish orange and the volatiles were removed *in vacuo*. The product was extracted with C_6H_6, filtered and the solvent was slowly evaporated. The product **9** was obtained as a yellow/orange solid in a yield of 75 % (+ 0.83 PPh_3). Crystals of **9** suitable for XRD were grown from a saturated pentane solution at −20 °C.

Analysis: **IR** (KBr) ν [cm^{-1}]: 3469*m*, 2962*s*, 2925*s*, 2856*s*, 2010*w* (C≡C), 1952*w*, 1915*s* (υ_{CO}, 1:1 ratio with 1844 cm^{-1}), 1844*s* (υ_{CO}, 1:1 ratio with 1915 cm^{-1}), 1586*w*, 1468*m*, 1376*m*, 1351*m*, 1243*m*, 1206*m*, 1175*m*, 1121*w*, 1022*w*, 1001*w*, 905*w*, 858*w*, 838*w*, 753*w*, 696*w*, 642*w*, 483*w*. **^{1}H NMR** (500 MHz, C_6D_6) δ [ppm]: 6.70 (*t*, $^3J_{HH}$ = 7.65 Hz, 1 H, C*H*$_{pyr(4)}$); 6.41 (*d*, $^3J_{HH}$ = 7.75 Hz, 2 H, C*H*$_{pyr(3,5)}$); 3.97 (*dt*, $^2J_{HH}$ = 15.37 Hz, $^2J_{HP}$ = 3.51 Hz, 2 H, C*H*$_2$); 3.03 (*dt*, $^2J_{HH}$ = 15.57 Hz, $^2J_{HP}$ = 2.99 Hz, 2 H, C*H*$_2$); 1.48 (*t*, $^3J_{PH}$ = 6.35 Hz, 18 H, ((C*H*$_3)_3$CP); 1.22 (*t*, $^3J_{PH}$ = 6.05 Hz, 18 H, ((C*H*$_3)_3$CP); 0.22 (*s*, 9 H, Si(C*H*$_3)_3$). **^{13}C{^{1}H} NMR** (125.72 MHz, C_6D_6) δ [ppm]: 163.8 (*t*, $^2J_{PC}$ = 3.89 Hz, 2 C, $C_{pyr(2,6)}$); 136.0 (*s*, 1 C, $CH_{pyr(4)}$); 121.2 (1 C, Tc−C≡*C*−Si, from ^{13}C−^{1}H HMBC); 119.9 (*t*, $^3J_{PC}$ = 3.90 Hz, 2 C, $CH_{pyr(3,5)}$); 39.6 (*t*, $^1J_{PC}$ = 5.48 Hz, 2 C, P*C*H_2); 37.7 (*m*, 4 C, P*C*$(CH_3)_2$); 31.8 (*t*-like *m*, 6 C, P(C(*C*$H_3)_3)_2$); 30.6 (*t*-like *m*, 6 C, P(C(*C*$H_3)_3)_2$); 1.6 (*s*, 3 C, (Si(*C*$H_3)_3$) missing signals for 2 *C*O. **^{31}P{^{1}H} NMR** (202.46 MHz, C_6D_6) δ [ppm]: 92.6 (*bs*, 2 H). **^{29}Si NMR** (99.36 MHz, C_6D_6) δ [ppm]: -29.31 (*s*, 1 H). **^{99}Tc NMR** (112.57 MHz, C_6D_6) δ [ppm]: -1435 ($\Delta_{1/2}$ = 15.7 kHz). **Vis** (C_6H_6) λ_{max} 401 (511), λ_{max} 568 (183), λ_{sh} 640 (21), λ_{max} 896 (76). **^{99}Tc analysis** Calc. for $C_{30}H_{52}NO_2P_2SiTc$ (·0.83 $C_{18}H_{15}P$) (%): 11.43. Found: 9.93.

3.8 [Tc($^{PyrPNP^{tBu}}$)(CO)$_2$(DMIM)] (10)

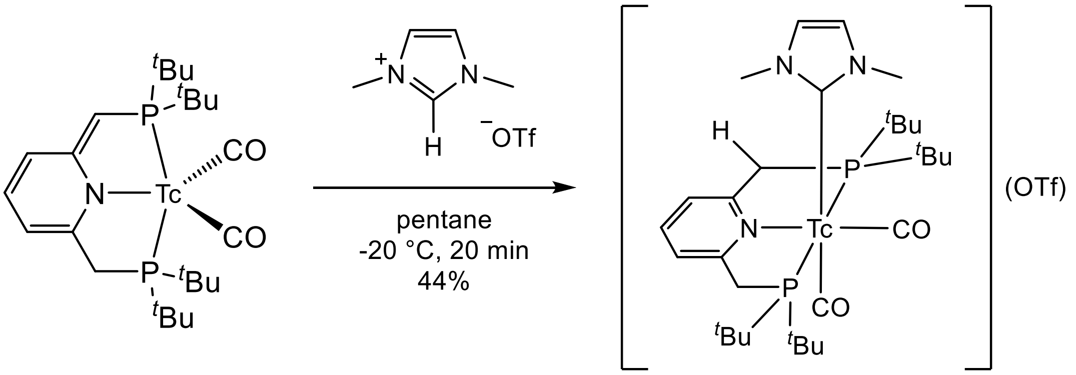

[**4**] [**10**]

Synthesis: Freshly prepared **4** (10.2 mg, 18.5 µmol) was dissolved in pentane (3 mL) in a round-bottom flask and cooled to -20 °C. A solution of [DMIM](OTf)) (4.6 mg, 18.5 µmol, 1 equiv.) in THF (0.5 mL) was prepared and added to the stirred, cooled solution. The color of the solution rapidly turned yellowish and yellowish precipitate was observed. After stirring the mixture for 14 h the volatiles were evaporated in vacuo. The crude product was extracted with C_6H_6 and filtered. The solvent was evaporated by slow ambient evaporation to yield **10** as a dark yellow solid in 44% yield (+ 1 PPh_3).

Analysis: **IR** (KBr) ν [cm^{-1}]: 3434*w*, 2919*s*, 2850*m*, 1910*m* (υ_{CO}, 1:1 ratio with 1822 cm^{-1}), 1822*m* (υ_{CO}, 1:1 ratio with 1910 cm^{-1}), 1597*w*, 1540*w*, 1464*m*, 1261*m*, 1178*w*, 1035*m*, 809*w*, 645*w*. **^{1}H NMR** (500 MHz, C_6D_6) δ [ppm]: 6.58 (*t*, $^3J_{HH}$ = 7.37 Hz, 1 H, C$H_{pyr(4)}$); 6.38 (*d*, $^3J_{HH}$ = 8.65 Hz, 1 H, C$H_{pyr(5)}$); 5.89 (*s*, 1 H, CH_{im}); 5.73 (*s*, 1 H, CH_{im}); 5.63 (*d*, $^3J_{HH}$ = 6.30 Hz, 1 H, C$H_{pyr(3)}$); 3.72 (*s*, 3 H, N–^{Im}CH_3); 3.37 (*s*, 3 H, N–^{Im}CH_3); 3.15 (*dd*, $^2J_{HH}$ = 15.59 Hz, $^2J_{HP}$ = 6.99 Hz, 2 H, PCH_2); 2.72 (*dd*, $^2J_{HH}$ = 15.52 Hz, $^2J_{HP}$ = 8.37 Hz, 2 H, PCH_2); 1.63 (*d*, $^3J_{HP}$ = 12.26 Hz, 9 H, (CH_3)$_3$CP); 1.40 (*d*, $^3J_{HP}$ = 12.26 Hz, 9 H, (CH_3)$_3$CP overlaid with impurities); 1.08 (*d*, $^3J_{HP}$ = 12.16 Hz, 9 H, (CH_3)$_3$CP overlaid with impurities); 0.65 (*d*, $^3J_{HP}$ = 11.36 Hz, 9 H, (CH_3)$_3$CP). **^{13}C{^{1}H} NMR** (125.72 MHz, C_6D_6) δ [ppm]: 214.9 (*dd*-like *m*, 1 C, F$_3$*C*); 172.0 (*dd*-like *m*, 1 C, *C*$_{pyr(6)}$); 160.0 (*dd*-like *m*, 1 C, *C*$_{pyr(2)}$); 131.9 (*s*, 1 C, *C*H$_{pyr(4)}$); 123.9 (*s*, 1 C, *C*H$_{Im}$); 123.5 (*s*, 1 C, *C*H$_{Im}$); 113.2 (*d*, $^3J_{PC}$ = 15.22 Hz, 1 C, *C*H$_{pyr(5)}$); 100.3 (*d*, $^3J_{PC}$ = 8.51 Hz, 1 C, *C*H$_{pyr(3)}$); 42.3 (*s*, 1 C, *C*H$_{3,\ Im}$); 40.62 (*d*, $^2J_{CP}$ = 16.15 Hz, 1 C, P(*C*(CH$_3$)$_3$)); 39.1 (*d*, $^1J_{PC}$ = 13.03 Hz, 2 C, P*C*H$_2$); 37.5 (*s*, 1 C, *C*H$_{3,\ Im}$); 31.7 (*d*, $^2J_{CP}$ = 4.82 Hz, 3 C, P(C(*C*H$_3$)$_3$)$_2$); 31.4 (*d*, $^2J_{CP}$ = 4.87 Hz, 3 C, P(C(*C*H$_3$)$_3$)$_2$); 30.5 (*d*, $^2J_{CP}$ = 4.25 Hz, 3 C, P(C(*C*H$_3$)$_3$)$_2$); 29.3 (*d*, $^2J_{CP}$ = 4.58 Hz, 3 C, P(C(*C*H$_3$)$_3$)$_2$); missing signals for 2 CO and carbene. **^{31}P{^{1}H} NMR** (202.46 MHz, C_6D_6) δ [ppm]: 82.23 (broad *s*, 1 P); 61.66 (broad *s*, 1 P). **^{99}Tc NMR** (112.57 MHz, C_6D_6) δ [ppm]: –1277 ($\Delta_{1/2}$ = 5.5 kHz). **Vis** (C_6H_6) λ_{max} 449 (626), λ_{max} 564 (153), λ_{max} 694 (68), λ_{max} 777 (30). **^{99}Tc analysis** Calc. for $C_{31}H_{51}F_3N_3O_5P_2STc$ (·1.05 $C_{18}H_{15}P$) (%): 9.23. Found: 7.05.

3.9 [Tc(^{Pyr}PNP^{tBu})(CO)$_2$(NC$_4$H$_4$)] (11)

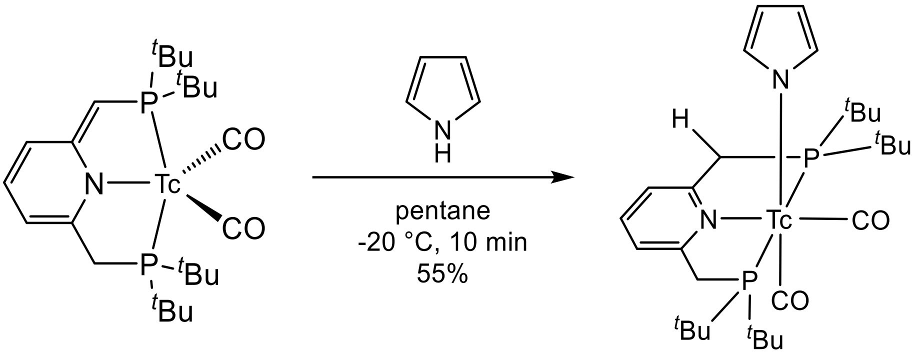

[**4**] [**11**]

Synthesis: Freshly prepared **4** (4.5 mg, 8.1 µmol) was dissolved in pentane (3 mL) in a round-bottom flask and cooled to -20 °C. Subsequently, 10 µl of pyrrole (18 equiv.) was added to the stirred solution and left to warm to room temperature. After 10 min the color of the solution had changed to yellow, and the mixture was stirred for 11 h. The remaining volatiles were removed *in vacuo*, the product was extracted in C_6H_6, filtered and the solvent was slowly evaporated. The product **11** was obtained as a yellowish solid in a yield of 55 % (+ 0.44 PPh_3). Crystals of **11** suitable for XRD were grown from a saturated pentane solution at −20 °C.

Analysis: **IR** (KBr) ν [cm^{-1}]: 3434*m*, 2923*s*, 2854*s*, 1919*s* (υ_{CO}, 1:1 ratio with 1828 cm^{-1}), 1851*m*, 1828*s* (υ_{CO}, 1:1 ratio with 1919 cm^{-1}), 1628*w*, 1597*w*, 1457*m*, 1369*w*, 1274*w*, 1274*w*, 1092*w*, 1180*w*, 1096*w*, 1025*w*, 716*w*. **^{1}H NMR** (500 MHz, C_6D_6) δ [ppm]: 7.69 (broad *s*, 1 H, C$H_{pyrrole(29)}$); 6.79 (*t*, $^3J_{HH}$ = 7.68 Hz, 1 H, C$H_{pyr(4)}$); 6.69 (broad *s*, 1 H, C$H_{pyrrole(28)}$); 6.60 (broad *s*, 1 H, C$H_{pyrrole(27)}$); 6.45 (*d*, $^3J_{HH}$ = 7.70 Hz, 2 H, C$H_{pyr(3,5)}$); 6.10 (broad *s*, 1 H, C$H_{pyrrole(26)}$); 3.66 (*d*, $^2J_{HH}$ = 16.01 Hz, 2 H, PCH_2); 3.01 (*d*, $^2J_{HH}$ = 15.96 Hz, 2 H, PCH_2); 1.21 (*t*, $^3J_{PH}$ = 6.08 Hz, 18 H, (PC(CH_3)$_3$); 0.86 (*t*, $^3J_{PH}$ = 6.20 Hz, 18 H, (PC(CH_3)$_3$). **^{13}C{^{1}H} NMR** (125.72 MHz, C_6D_6) δ [ppm]: 164.9 (*t*, $^2J_{PC}$ = 4.03 Hz, 2 C, $C_{pyr(2,6)}$); 137.2 (*s*, 1 C, *C*H$_{pyrrole(26)}$); 136.8 (*s*, 1 C, *C*H$_{pyr(4)}$); 126.9 (*s*, 1 C, *C*H$_{pyrrole(29)}$); 120.8 (*t*, $^3J_{PC}$ = 3.74 Hz, 2 C, *C*H$_{pyr(3,5)}$); 108.3 (*s*, 1 C, *C*H$_{pyrrole}$); 107.2 (*s*, 1 C, *C*H$_{pyrrole}$); 37.9 (*t*, $^1J_{PC}$ = 4.70 Hz, 2 C, P*C*H$_2$); 37.2 (*t*, $^1J_{PC}$ = 6.41 Hz, 2 C, P(*C*(CH$_3$)$_3$)$_2$); 36.8 (*t*, $^1J_{PC}$ = 4.61 Hz, 2 C, P(*C*(CH$_3$)$_3$)$_2$); 30.4 (*t*-like *s*, 6 C, P(C(*C*H$_3$)$_3$)$_2$); 30.0 (*t*-like *s*, 6 C, P(C(*C*H$_3$)$_3$)$_2$); missing signals for 2 *C*O. **^{31}P{^{1}H} NMR** (202.46 MHz, C_6D_6) δ [ppm]: 79.44 (broad *m*, 2 P). **^{99}Tc NMR** (112.57 MHz, C_6D_6) δ [ppm]: –1000 ($\Delta_{1/2}$ = 4.0 kHz). **Vis** (C_6H_6) λ_{max} 407 (324), λ_{sh} 603 (56), λ_{max} 704 (22). **^{99}Tc analysis** Calc. for $C_{29}H_{47}N_2O_2P_2Tc$ (·0.44 $C_{18}H_{15}P$) (%): 13.48. Found: 14.18.

3.10 [Tc(^{Pyr}PNP^{tBu})(CO)$_2$(N$_2$C$_3$H$_3$)] (12)

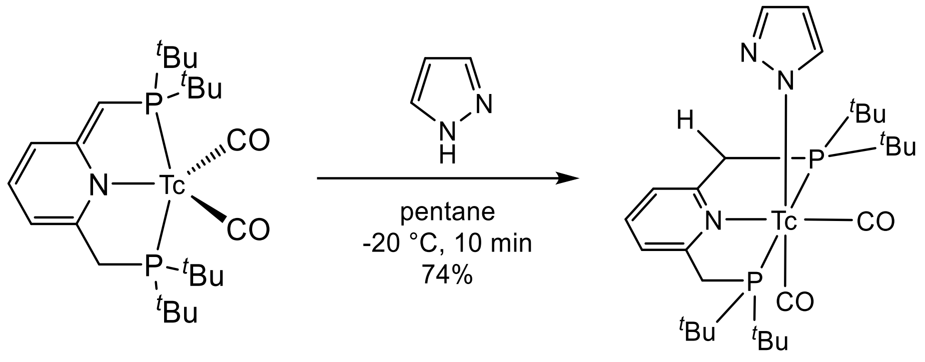

[**4**] [**12**]

Synthesis: Freshly prepared **4** (2.2 mg, 4.0 µmol) was dissolved in pentane (2 mL) in a round-bottom flask and cooled to -20 °C. Subsequently, pyrazole (1.75 equiv.) was added to the stirred solution as a solution in THF (0.07 M) and left to warm to room temperature. After 10 min the color of the solution had changed to yellow and the mixture was stirred for 16 h. The remaining volatiles were removed *in vacuo*, the product was extracted in C_6H_6, filtered and the solvent was slowly evaporated. The product **12** was obtained as a yellowish solid in a yield of 74 % (+ 0.54 PPh_3). Crystals of **12** suitable for XRD were grown from a saturated pentane solution at −20 °C.

Analysis: **IR** (KBr) ν [cm^{-1}]: 3434*m*, 2923*s*, 2853*s*, 1920*s* (υ_{CO}, 1:1 ratio with 1838 cm^{-1}), 1838*s* (υ_{CO}, 1:1 ratio with 1920 cm^{-1}), 1629*w*, 1458*m*, 1369*w*, 1276*w*, 1179*w*, 1092*w*, 1029*w*, 845*w*, 742*w*, 696*w*, 626*w*. **^{1}H NMR** (500 MHz, C_6D_6) δ [ppm]: 8.25 (broad *s*, 1 H, C$H_{pyrazole}$); 7.83 (broad *s*, 1 H, C$H_{pyrazole}$); 6.85 (*t*, $^3J_{HH}$ = 7.75 Hz, 1 H, C$H_{pyr(4)}$); 6.55 (*d*, $^3J_{HH}$ = 7.70 Hz, 2 H, C$H_{pyr(3,5)}$); 6.50 (broad *s*, 1 H, C$H_{pyrazole}$); 4.47 (*dt*, $^2J_{HH}$ = 15.43 Hz, $^2J_{HP}$ = 3.51 Hz, 2 H, PCH_2); 3.10 (*dt*, $^2J_{HH}$ = 15.58 Hz, $^2J_{HP}$ = 2.86 Hz, 2 H, PCH_2); 1.23 (*t*, $^3J_{PH}$ = 6.15 Hz, 18 H, (PC(CH_3)$_3$); 0.82 (*t*, $^3J_{PH}$ = 6.23 Hz, 18 H, (PC(CH_3)$_3$). **^{13}C{^{1}H} NMR** (125.72 MHz, C_6D_6) δ [ppm]: 165.1 (*t*, $^2J_{PC}$ = 3.70 Hz, 2 C, $C_{pyr(2,6)}$); 145.7 (*s*, 1 C, CH$_{pyrazole}$); 138.2 (*s*, 1 C, CH$_{pyrazole}$); 136.7 (*s*, 1 C, $C_{pyr(4)}$); 120.2 (*t*, $^3J_{PC}$ = 3.91 Hz, 2 C, CH$_{pyr(3,5)}$); 104.8 (*s*, 1 C, CH$_{pyrazole}$); 38.0 (*t*, $^1J_{PC}$ = 5.28 Hz, 2C, PCH$_2$); 37.2 (*t*, $^1J_{PC}$ = 6.40 Hz, 2 C, P(C(CH$_3$)$_3$)$_2$); 36.6 (*t*, $^1J_{PC}$ = 4.95 Hz, 2 C, P(C(CH$_3$)$_3$)$_2$); 30.6 (*t*, $^2J_{PC}$ = 2.27 Hz, 6 C, P(C(CH$_3$)$_3$)$_2$); 30.1 (*t*, $^2J_{PC}$ = 2.25 Hz, 6 C, P(C(CH$_3$)$_3$)$_2$); missing signals for 2 *C*O. **^{31}P{^{1}H} NMR** (202.46 MHz, C_6D_6) δ [ppm]: 76.95 (broad *s*, 2 P). **^{99}Tc NMR** (90.06 MHz, C_6D_6) δ [ppm]: –1013 ($\Delta_{1/2}$ = 4.0 kHz). **Vis** (C_6H_6) λ_{max} 418 (424), λ_{sh} 587 (144), λ_{max} 704 (62). **^{99}Tc analysis** Calc. for $C_{28}H_{46}N_3O_2P_2Tc$ (·0.54 $C_{18}H_{15}P$) (%): 13.03. Found: 11.61.

4 Spectra

4.1 [Tc(^{Pyr}PNP^{tBu})(CO)$_2$Cl] (3)

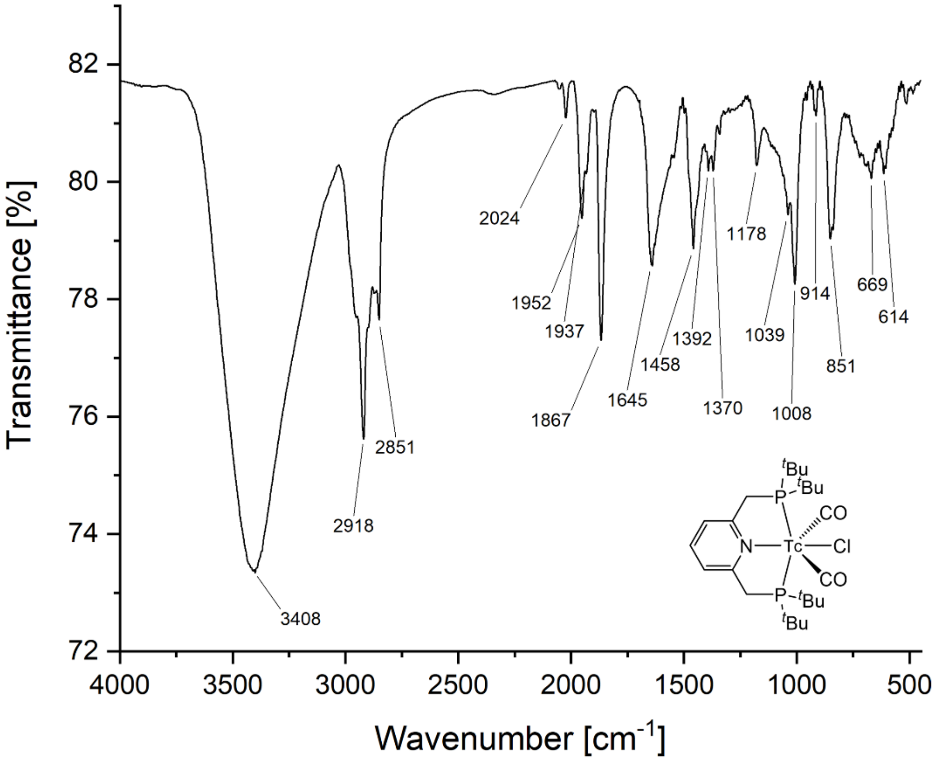

Figure S1: IR spectrum (KBr) of [Tc(^{Pyr}PNP^{tBu})(CO)$_2$Cl] (**3**).

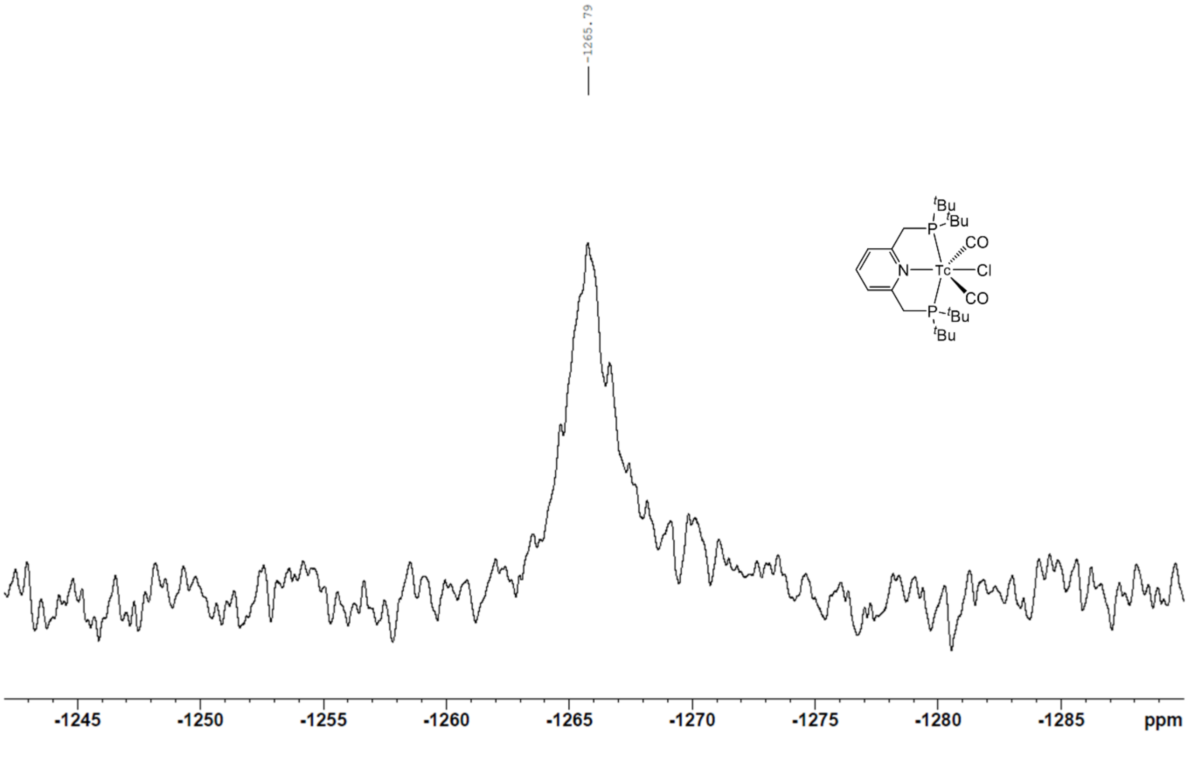

Figure S2: ^{99}Tc NMR spectrum of [Tc(^{Pyr}PNP^{tBu})(CO)$_2$Cl] (**3**).

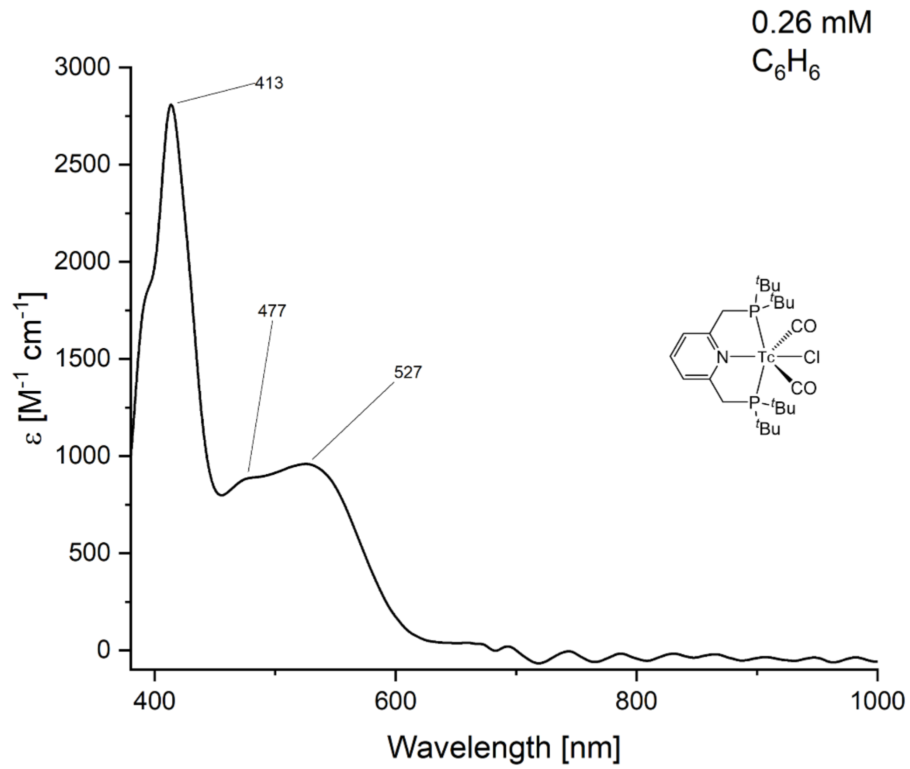

Figure S3: VIS spectrum (C_6H_6) of [Tc(^{Pyr}PNP^{tBu})$(CO)_2$Cl] (**3**).

4.2 [Tc(^{Pyr}PNP^{tBu*})$(CO)_2$] (4)

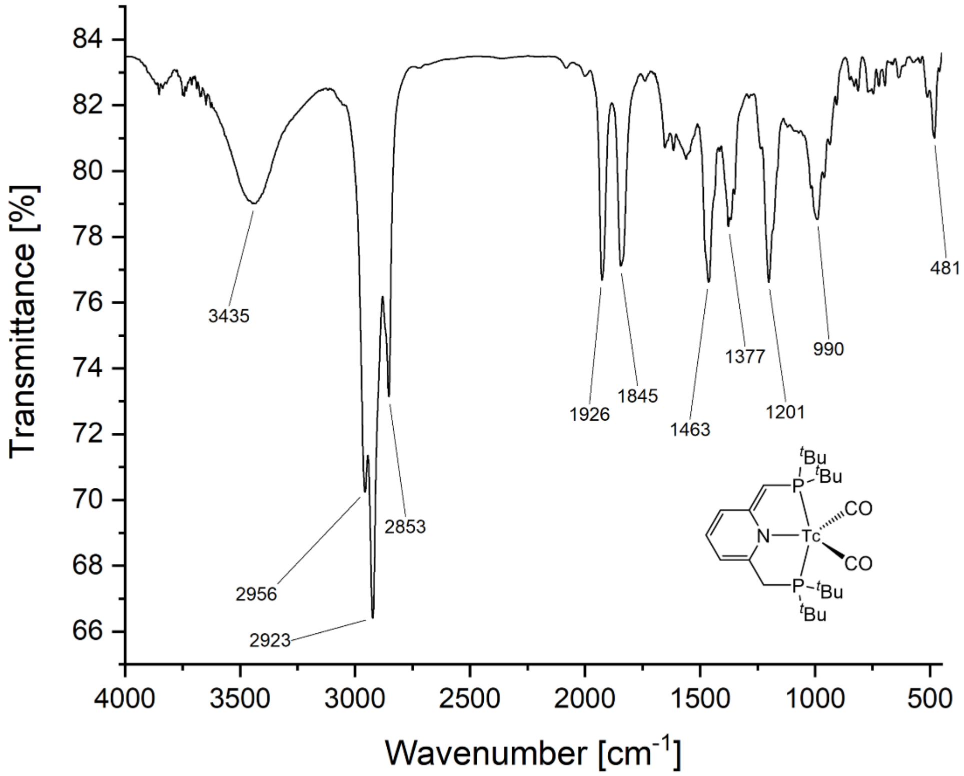

Figure S4: IR spectrum (KBr) of [Tc(^{Pyr}PNP^{tBu*})$(CO)_2$] (**4**).

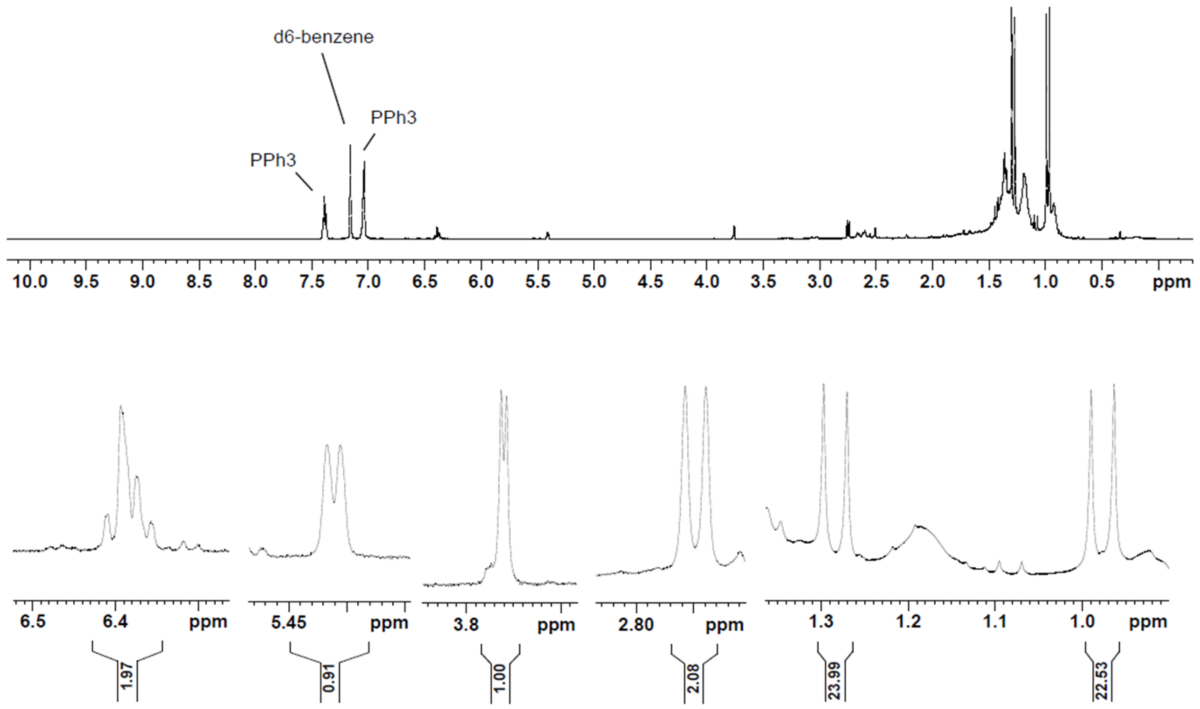

Figure S5: ^{1}H NMR spectrum of [Tc(^{Pyr}PNP^{tBu*})(CO)$_2$] (**4**).

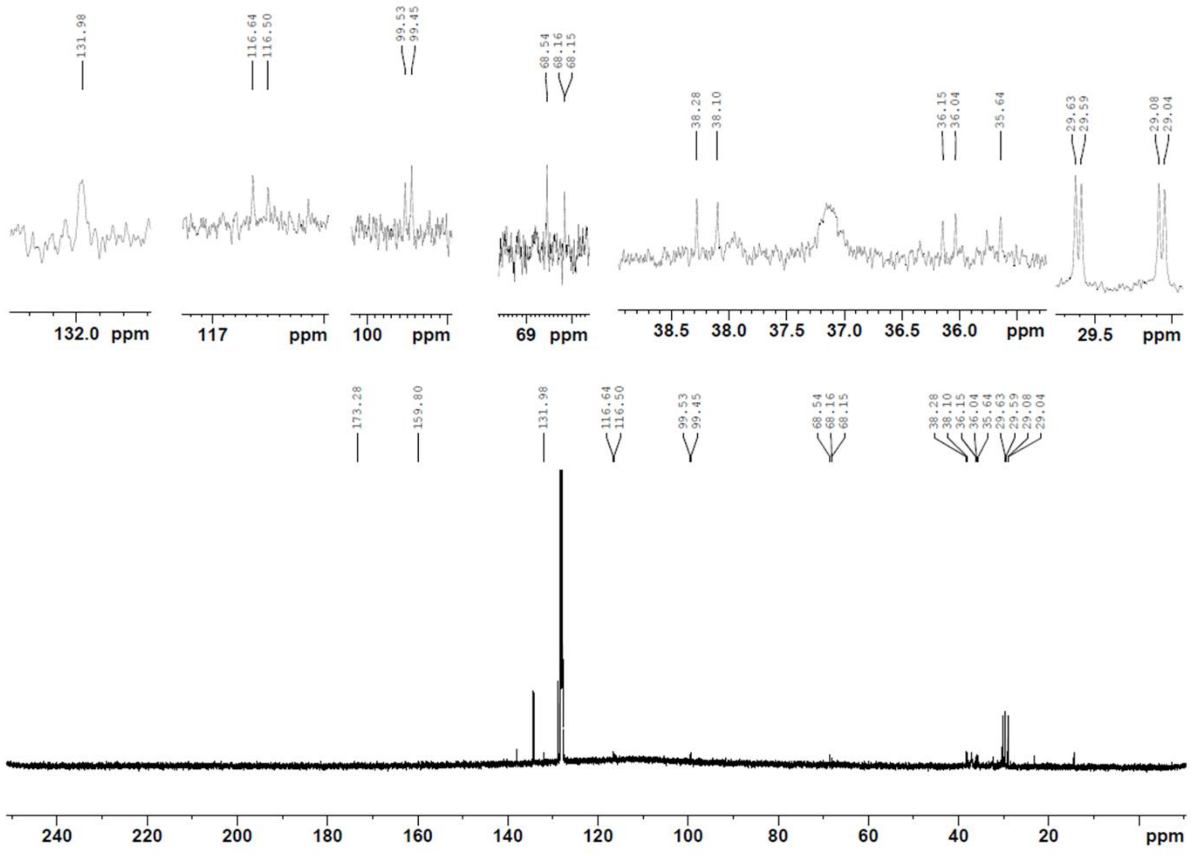

Figure S6: ^{13}C{^{1}H} NMR spectrum of [Tc(^{Pyr}PNP^{tBu*})(CO)$_2$] (**4**).

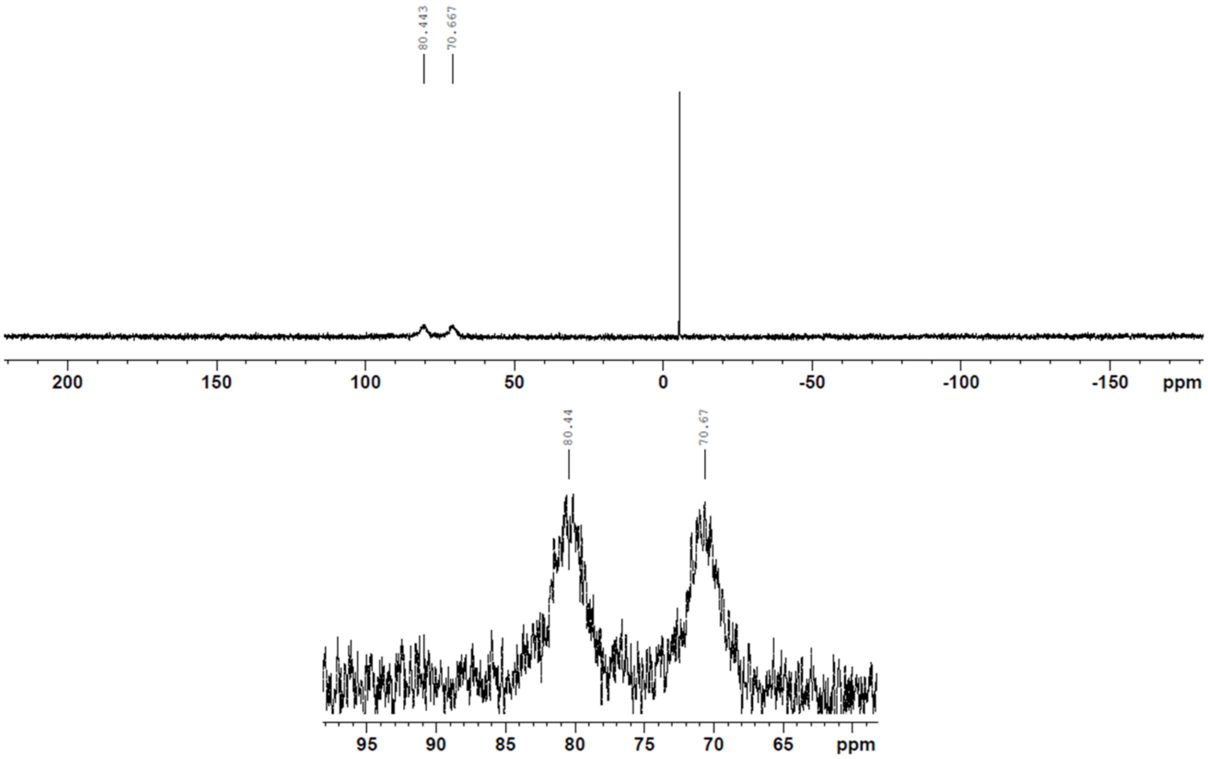

Figure S7: ^{31}P{^{1}H} NMR spectrum of [Tc(^{Pyr}PNP^{tBu*})(CO)$_2$] (**4**).

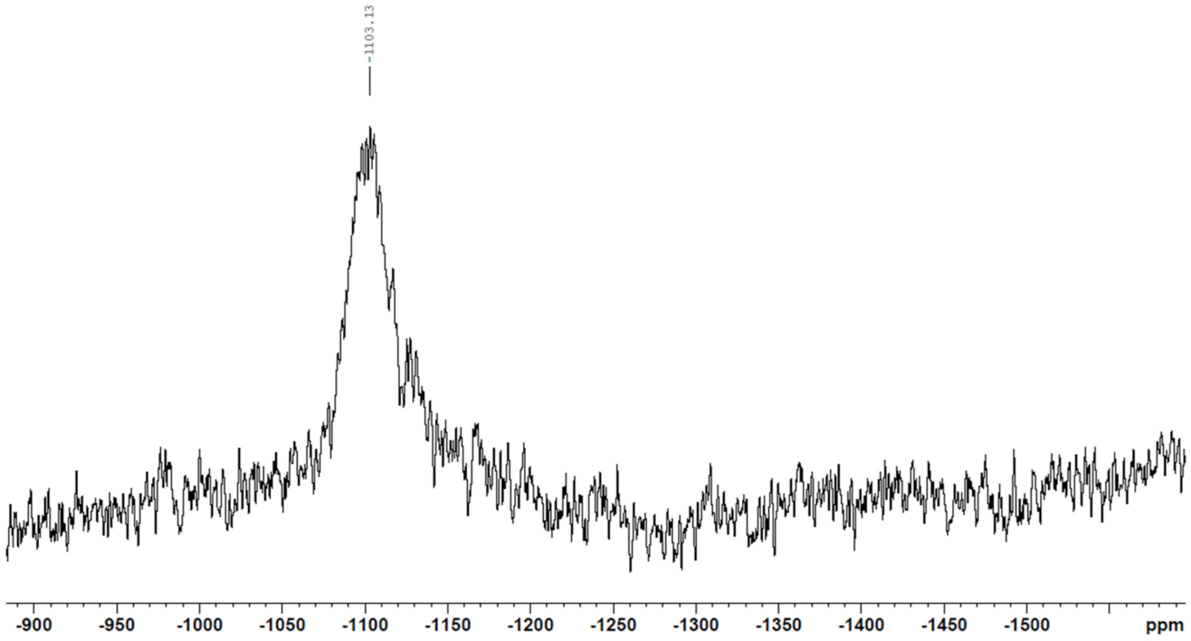

Figure S8: ^{99}Tc NMR spectrum of [Tc(^{Pyr}PNP^{tBu*})(CO)$_2$] (**4**).

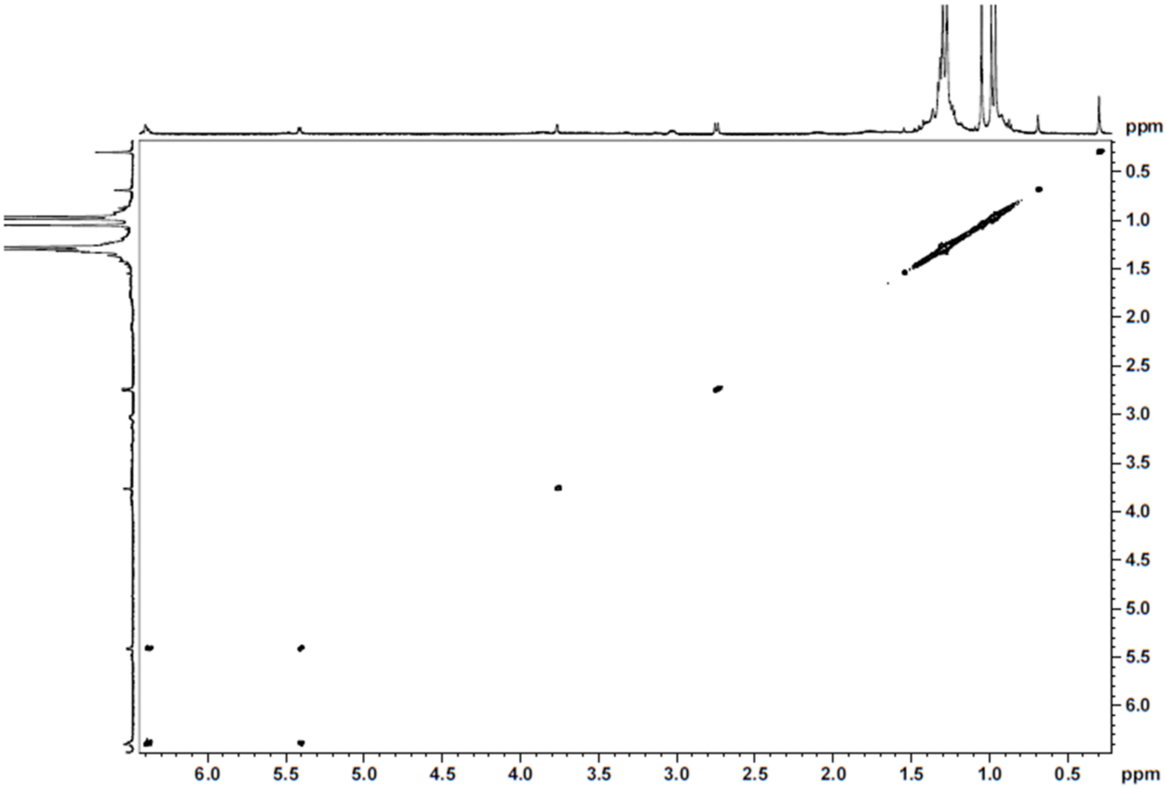

Figure S9: ^{1}H-^{1}H COSY NMR spectrum of [Tc(^{Pyr}PNP^{tBu*})(CO)$_2$] (**4**).

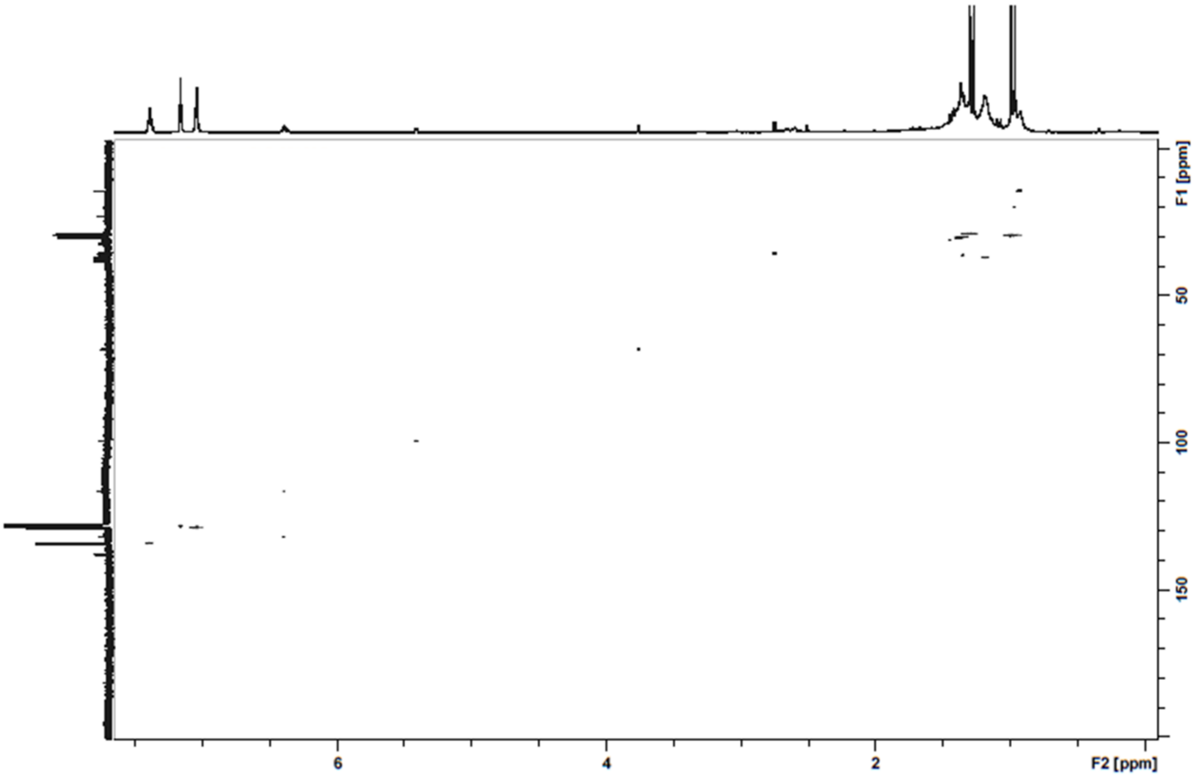

Figure S10: ^{1}H-^{13}C HSQC NMR spectrum of [Tc(^{Pyr}PNP^{tBu*})(CO)$_2$] (**4**).

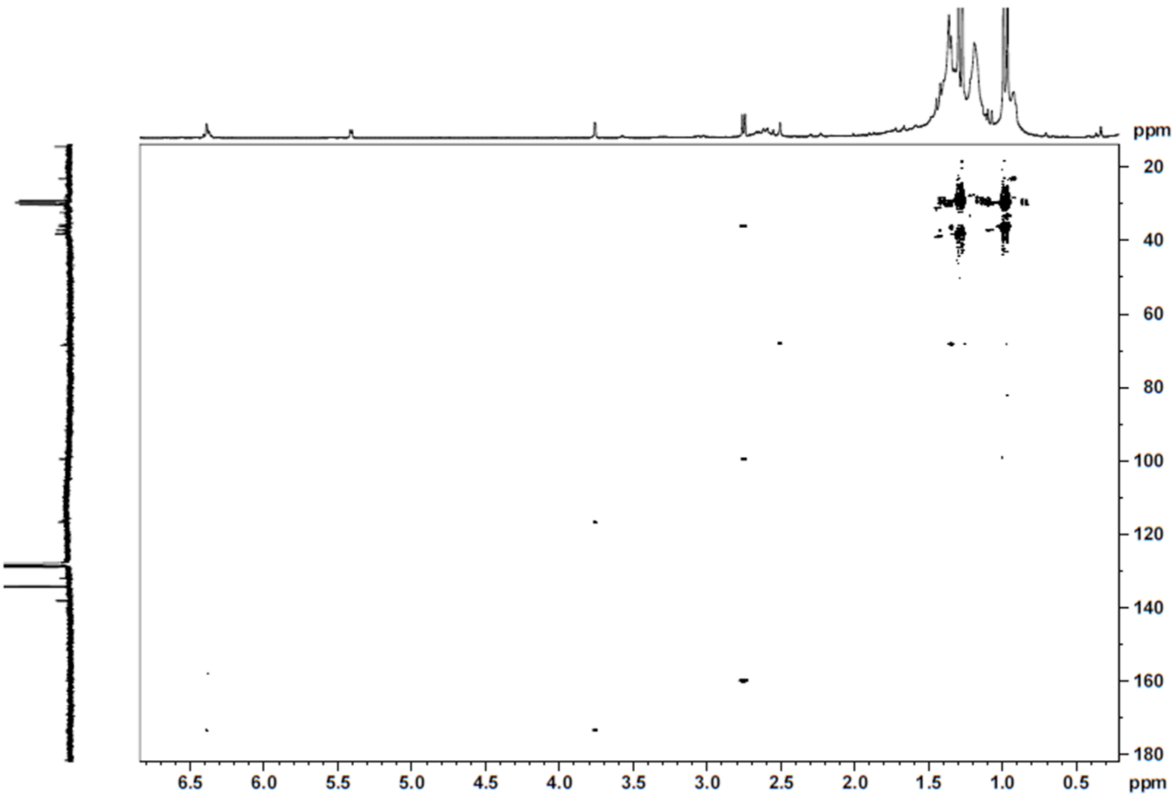

Figure S11: ^{1}H-^{13}C HMBC NMR spectrum of [Tc(^{Pyr}PNP^{tBu*})(CO)$_2$] (**4**).

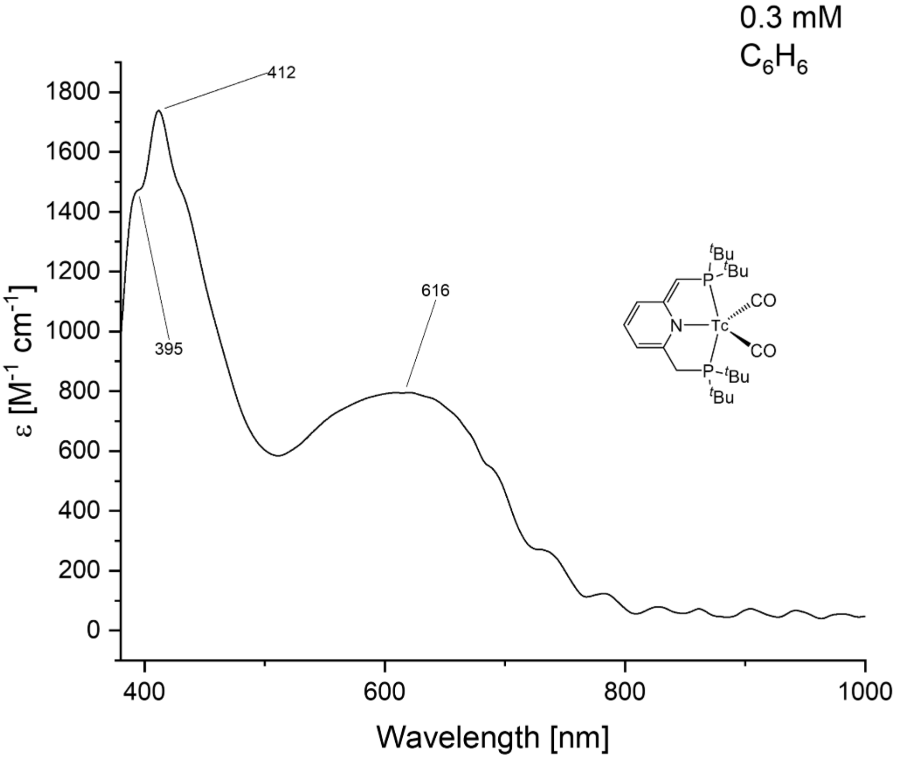

Figure S12: VIS spectrum (C_6H_6) of [Tc(^{Pyr}PNP^{tBu*})(CO)$_2$] (**4**).

4.3 [Tc(^{Pyr}PNP^{tBu}–COO)(CO)$_2$] (5)

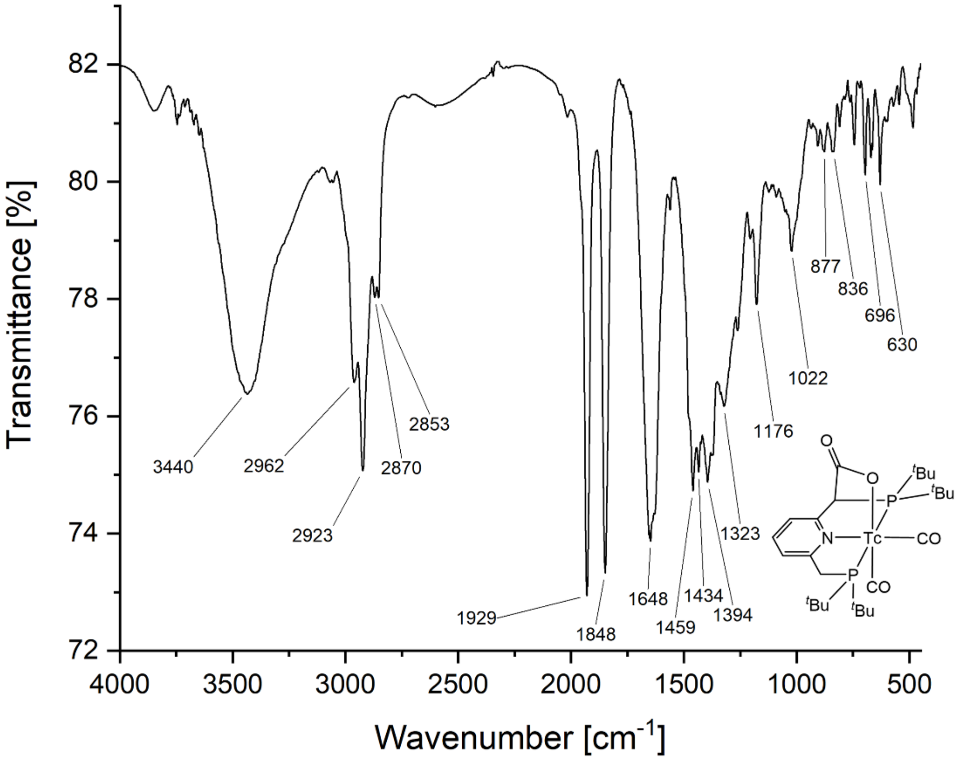

Figure S13: IR spectrum (KBr) of [Tc(^{Pyr}PNP^{tBu}–COO)(CO)$_2$] (**5**).

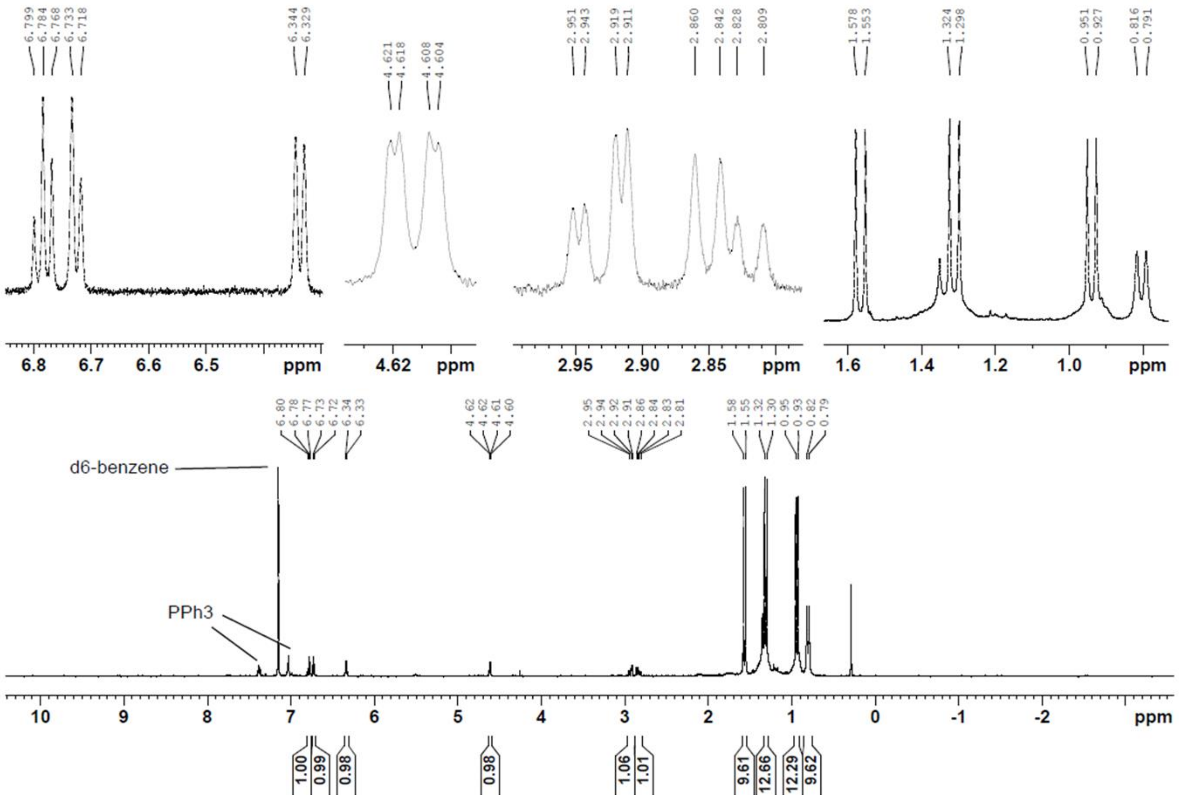

Figure S14: ^{1}H NMR spectrum of [Tc(^{Pyr}PNP^{tBu}–COO)(CO)$_2$] (**5**).

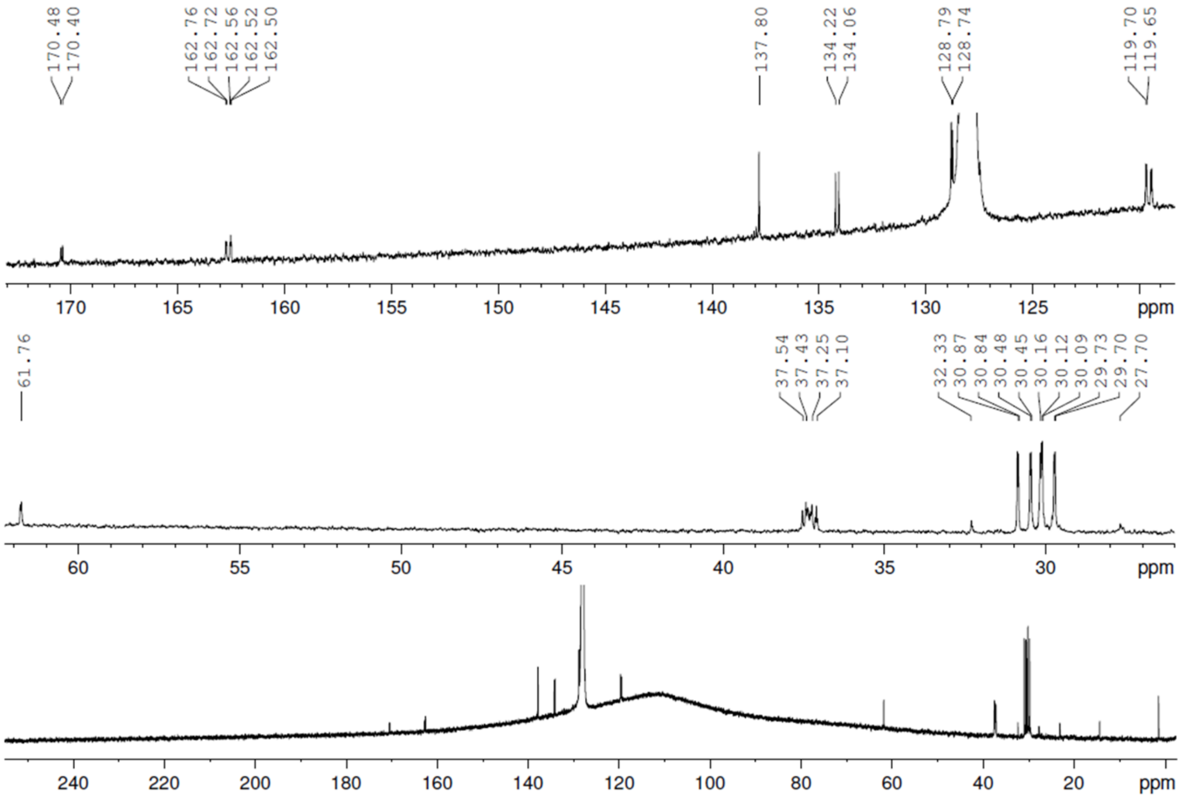

Figure S15: $^{13}C\{^{1}H\}$ NMR spectrum of $[Tc(^{Pyr}PNP^{tBu}–COO)(CO)_2]$ (**5**).

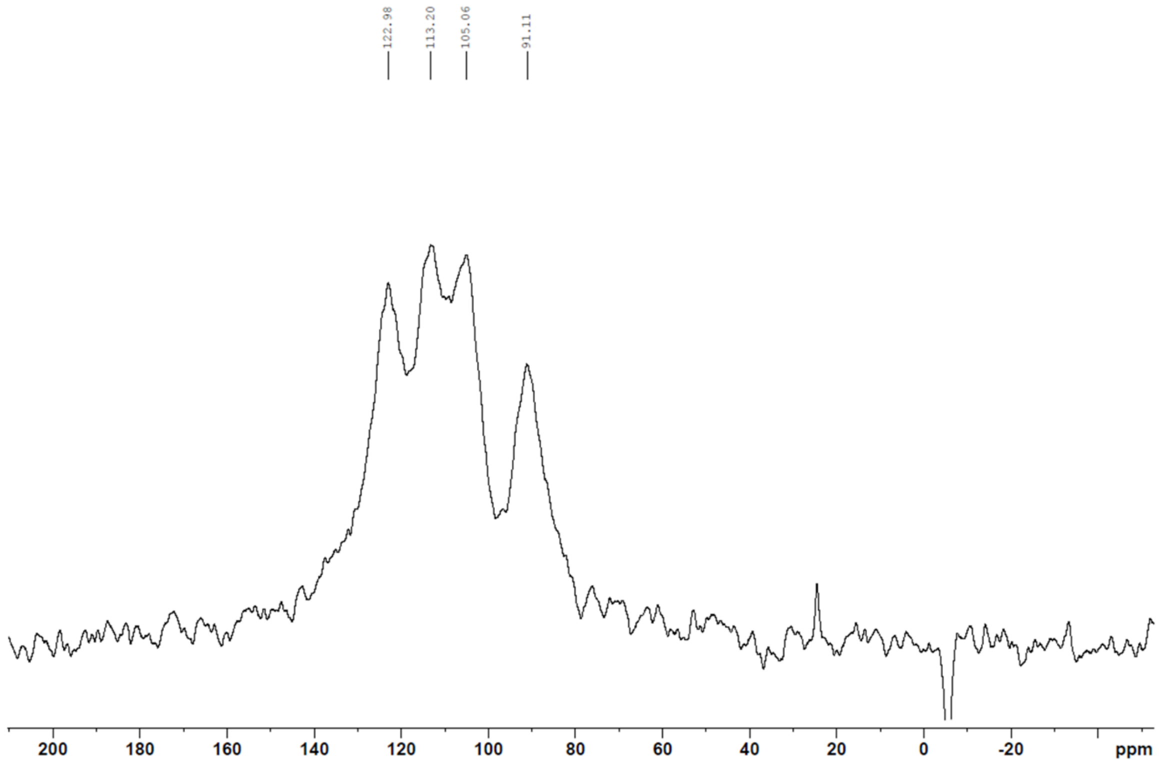

Figure S16: $^{31}P\{^{1}H\}$ NMR spectrum of $[Tc(^{Pyr}PNP^{tBu}–COO)(CO)_2]$ (**5**).

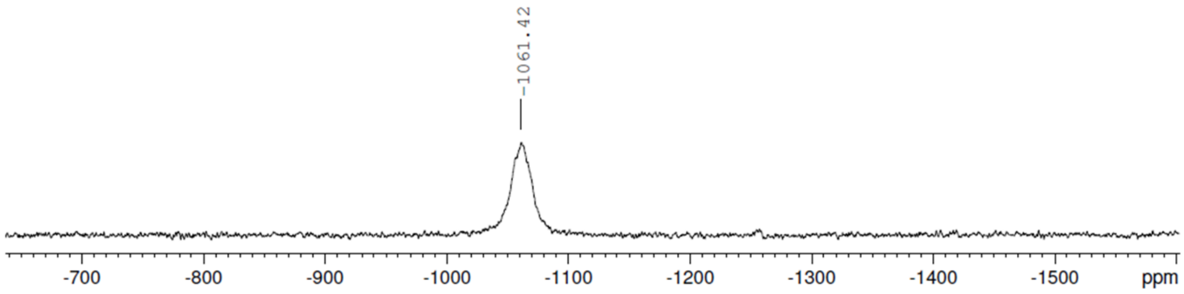

Figure S17: ^{99}Tc NMR spectrum of [Tc(^{Pyr}PNP^{tBu}–COO)(CO)$_2$] (**5**).

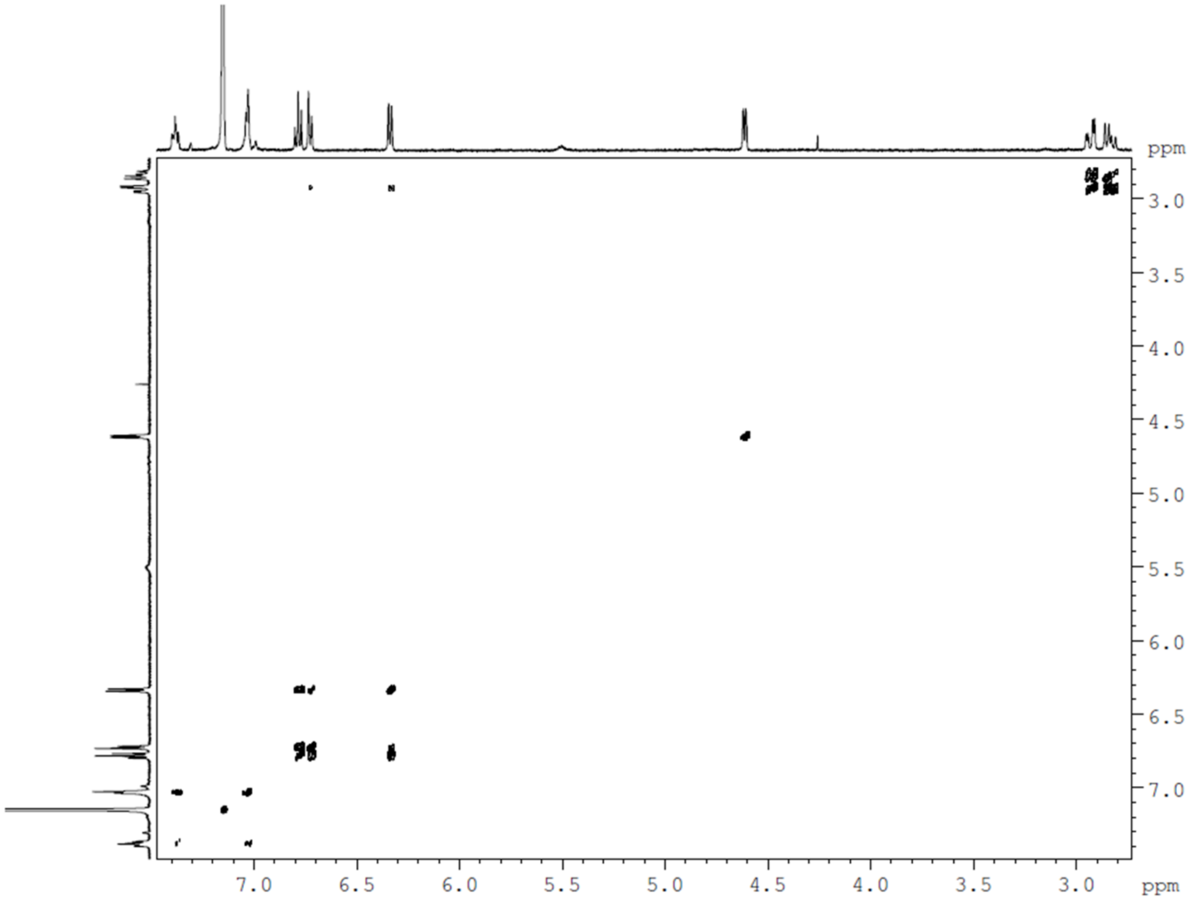

Figure S18: ^{1}H-^{1}H COSY NMR spectrum of [Tc(^{Pyr}PNP^{tBu}–COO)(CO)$_2$] (**5**).

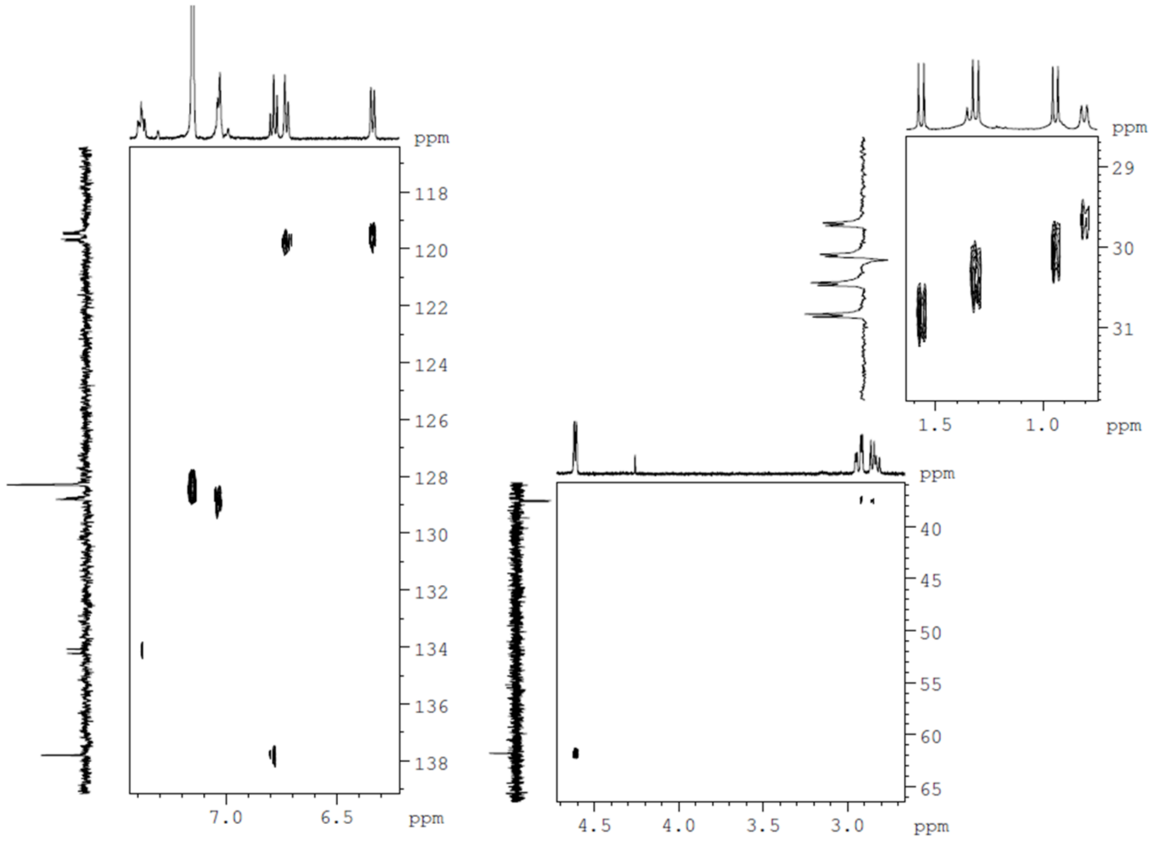

Figure S19: ^{1}H-^{13}C HSQC NMR spectrum of [Tc(^{Pyr}PNP^{tBu}–COO)$(CO)_2$] (**5**).

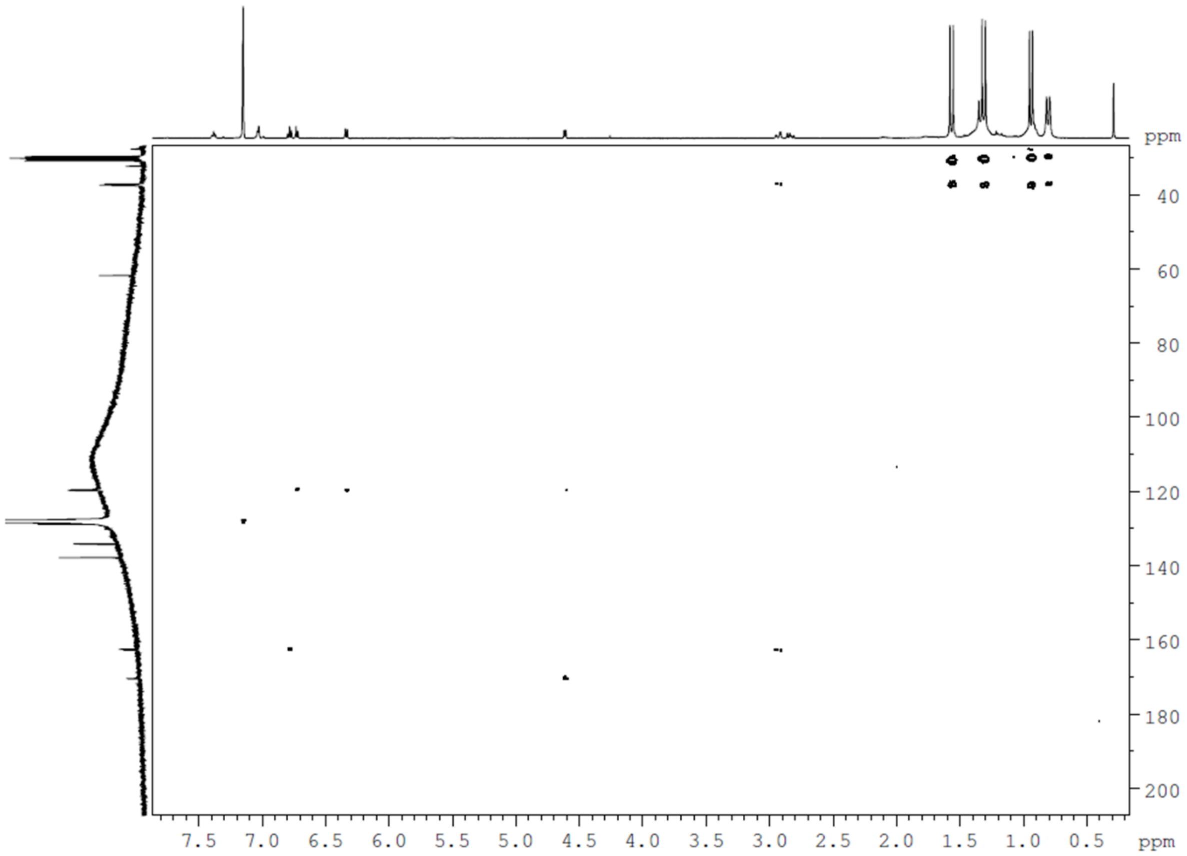

Figure S20: ^{1}H-^{13}C HMBC NMR spectrum of [Tc(^{Pyr}PNP^{tBu}–COO)$(CO)_2$] (**5**).

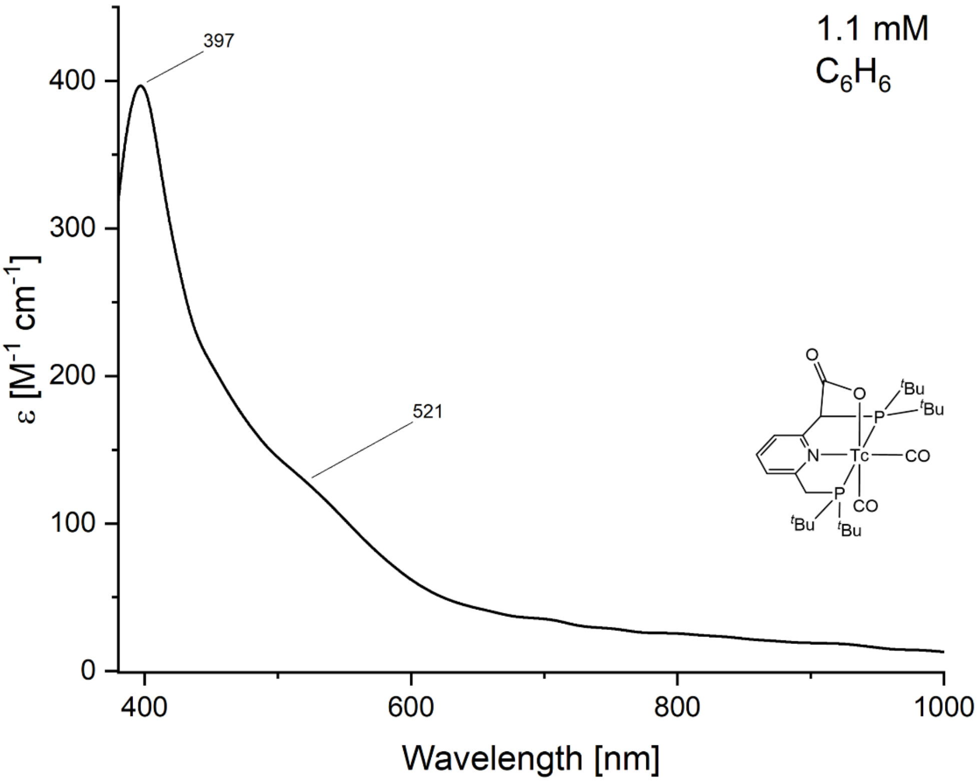

Figure S21: VIS spectrum (C_6H_6) of [Tc(^{Pyr}PNP^{tBu}–COO)$(CO)_2$] (**5**).

4.4 [Tc(^{Pyr}PNP^{tBu}–CSS)$(CO)_2$] (6)

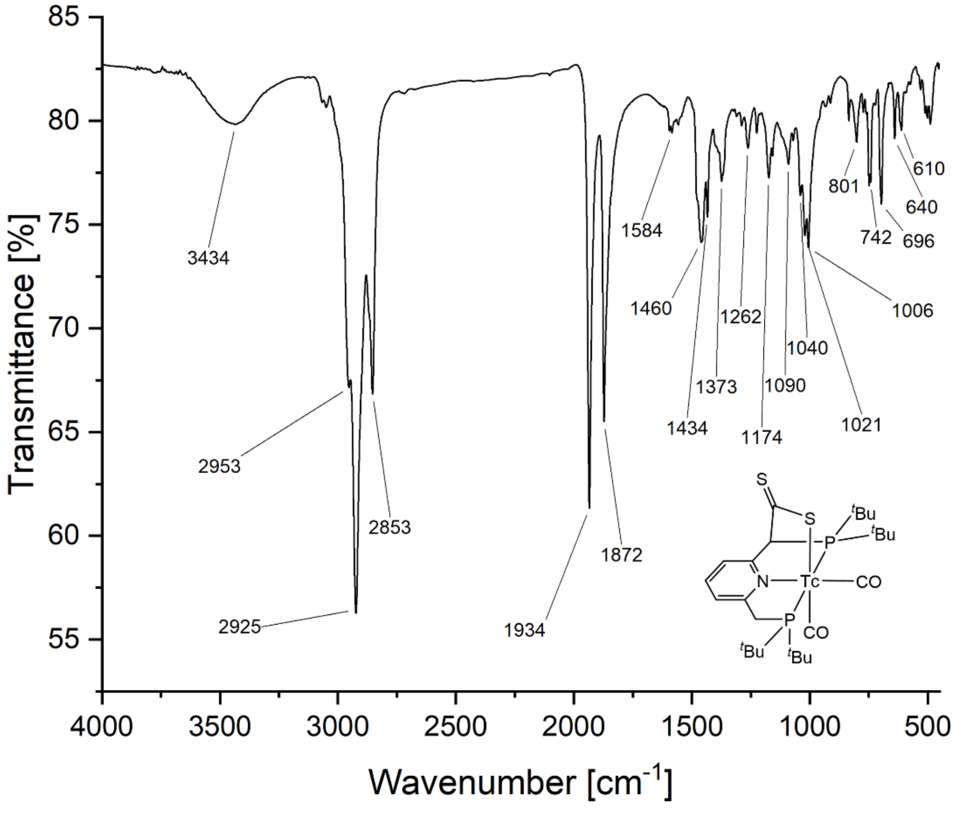

Figure S22: IR spectrum (KBr) of [Tc(^{Pyr}PNP^{tBu}–CSS)$(CO)_2$] (**6**).

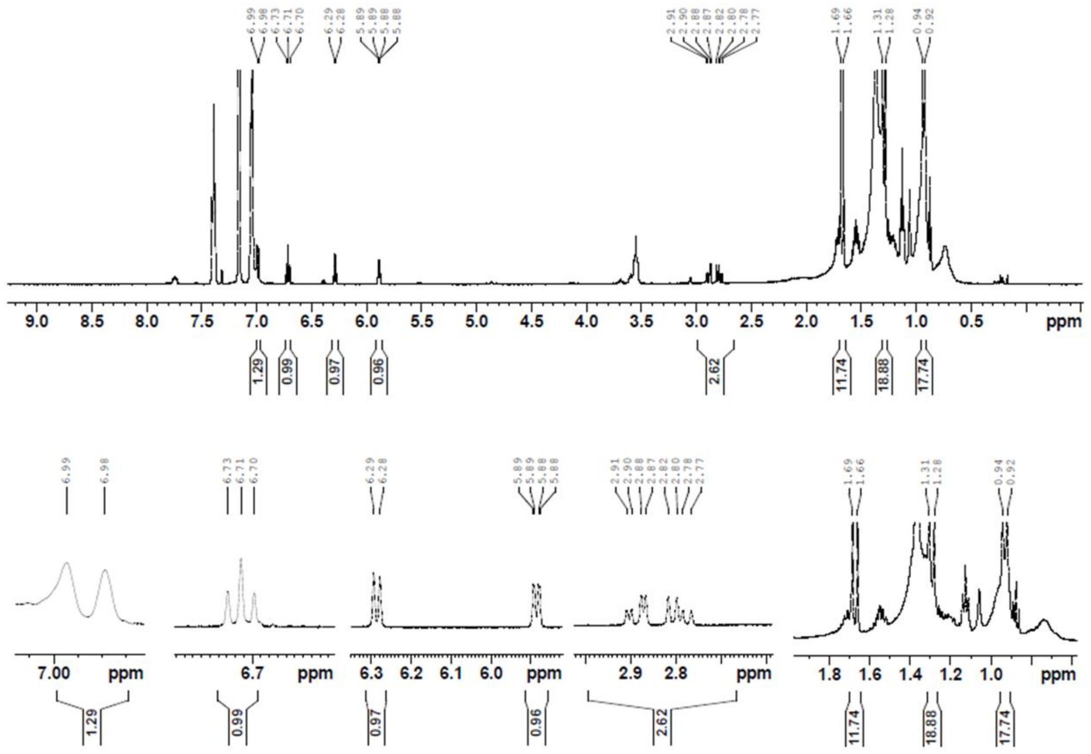

Figure S23: ^{1}H NMR spectrum of [Tc(^{Pyr}PNP^{tBu}–CSS)(CO)$_2$] (**6**).

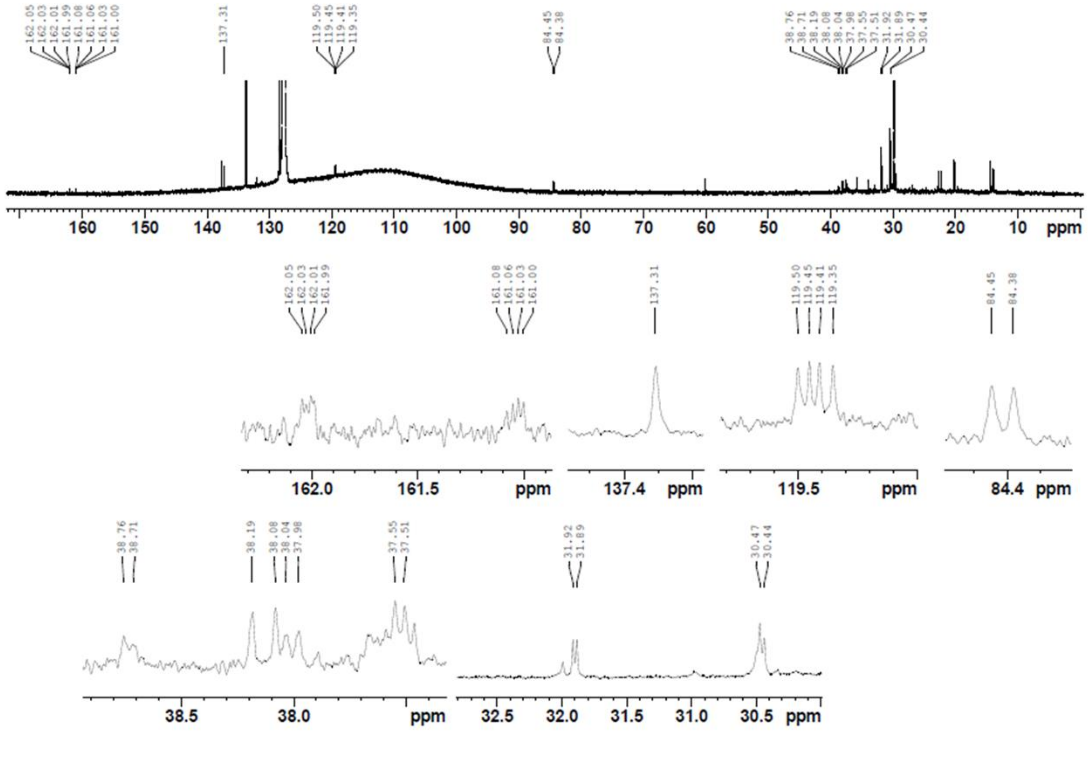

Figure S24: $^{13}C\{^{1}H\}$ NMR spectrum of [Tc(^{Pyr}PNP^{tBu}–CSS)(CO)$_2$] (**6**).

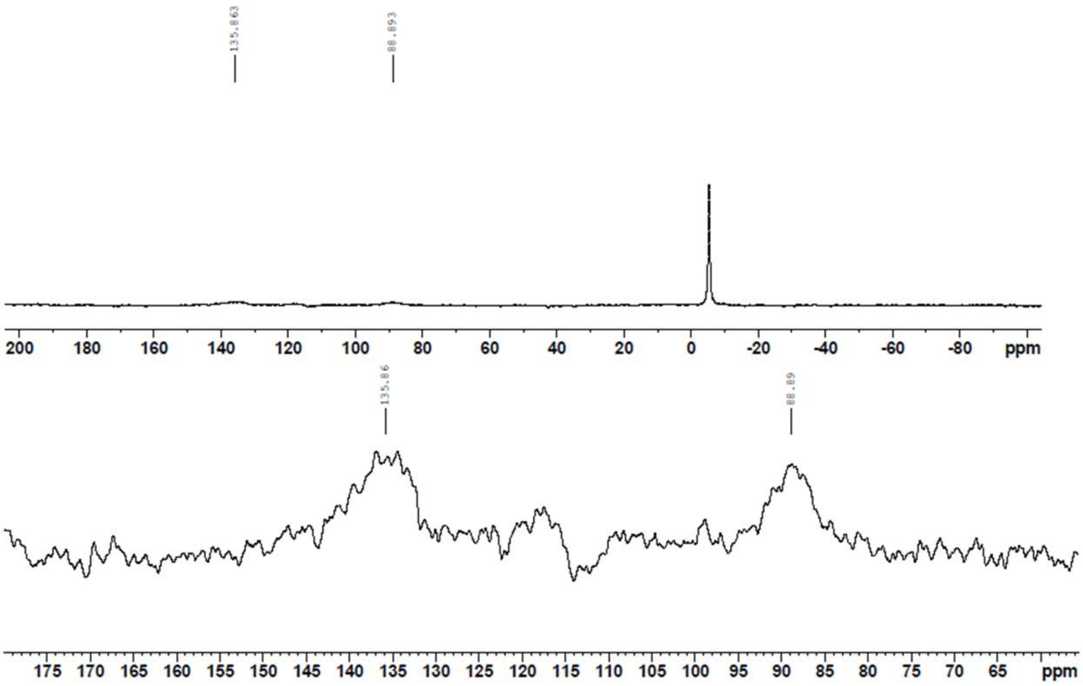

Figure S25: $^{31}P\{^{1}H\}$ NMR spectrum of [Tc(^{Pyr}PNP^{tBu}–CSS)$(CO)_2$] (**6**).

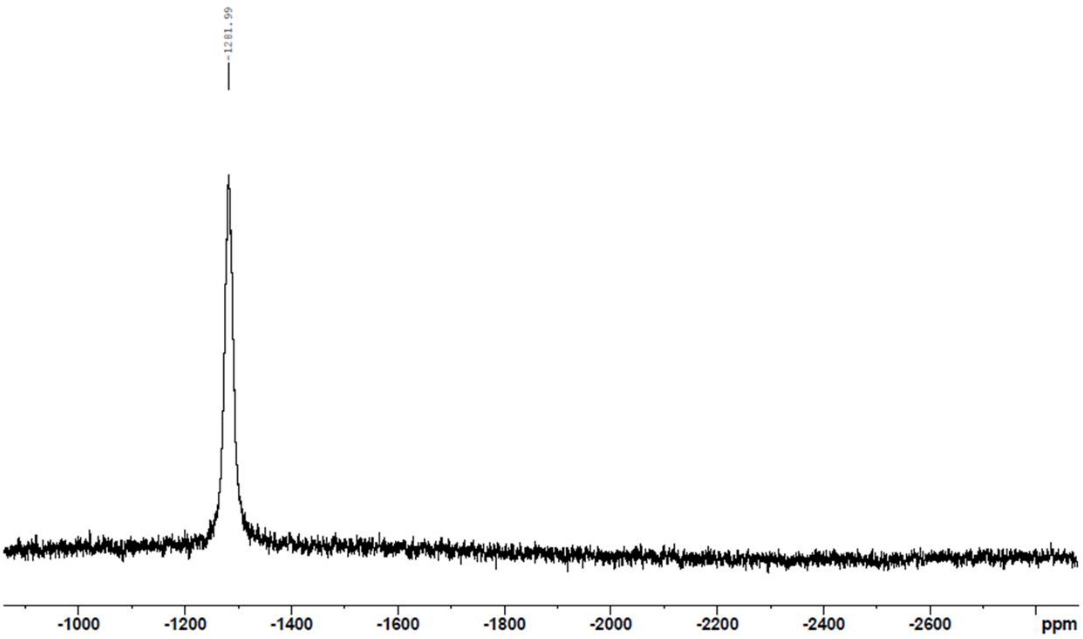

Figure S26: ^{99}Tc NMR spectrum of [Tc(^{Pyr}PNP^{tBu}–CSS)$(CO)_2$] (**6**).

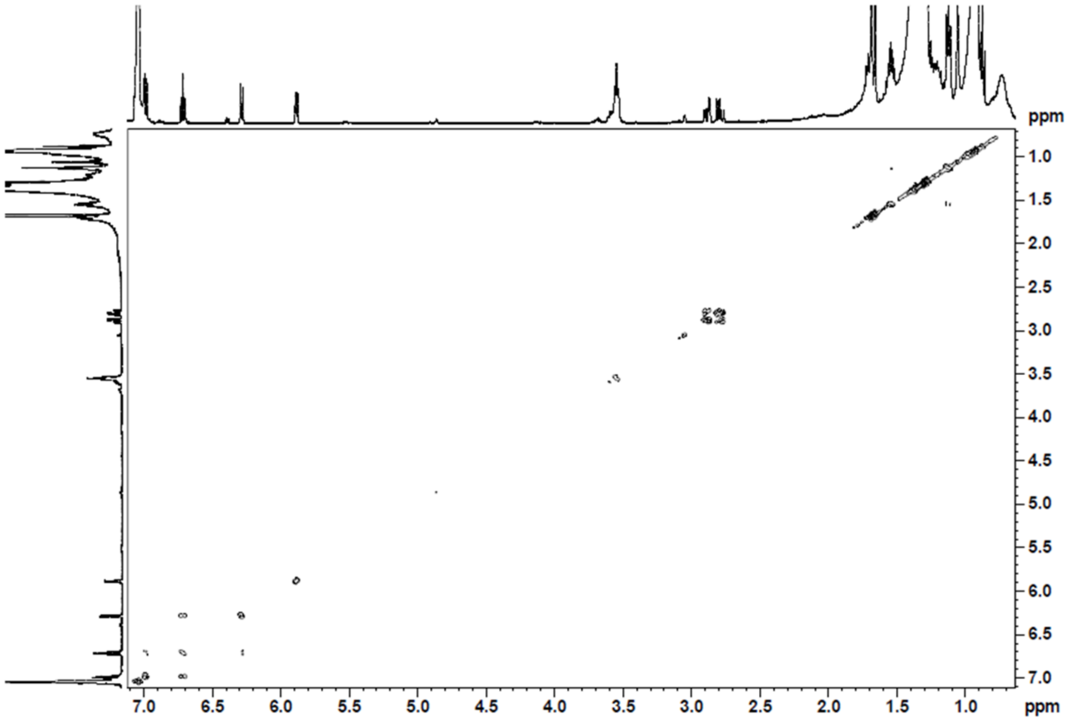

Figure S27: ^{1}H-^{1}H COSY NMR spectrum of [Tc(^{Pyr}PNP^{tBu}–CSS)$(CO)_2$] (**6**).

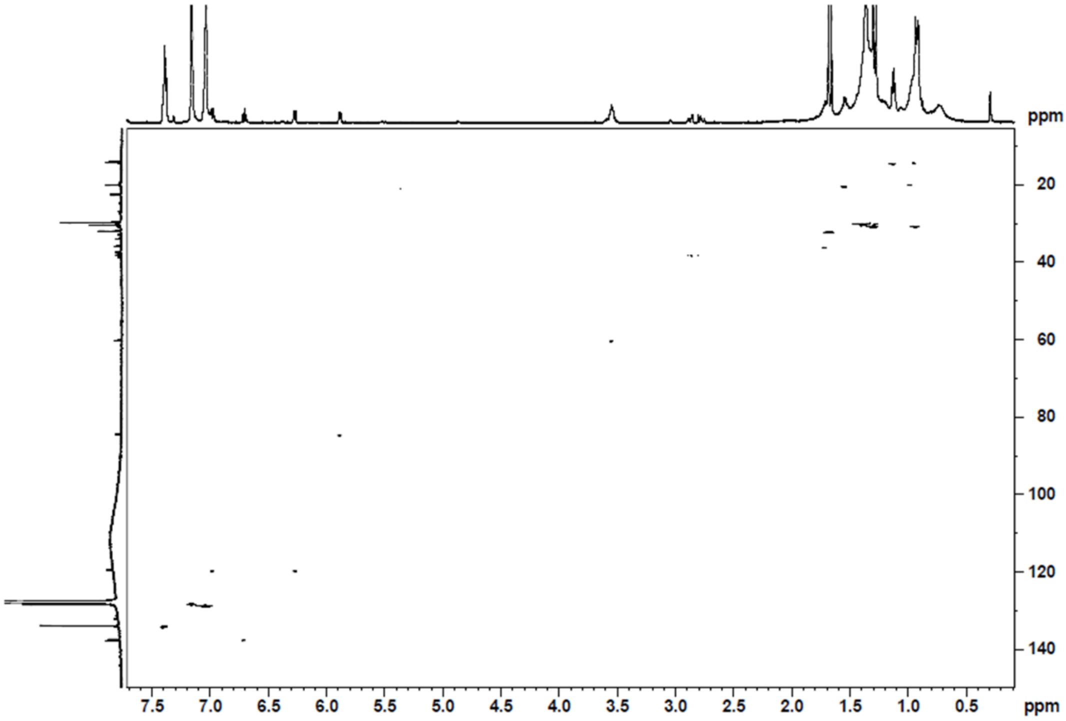

Figure S28: ^{1}H-^{13}C HSQC NMR spectrum of [Tc(^{Pyr}PNP^{tBu}–CSS)$(CO)_2$] (**6**).

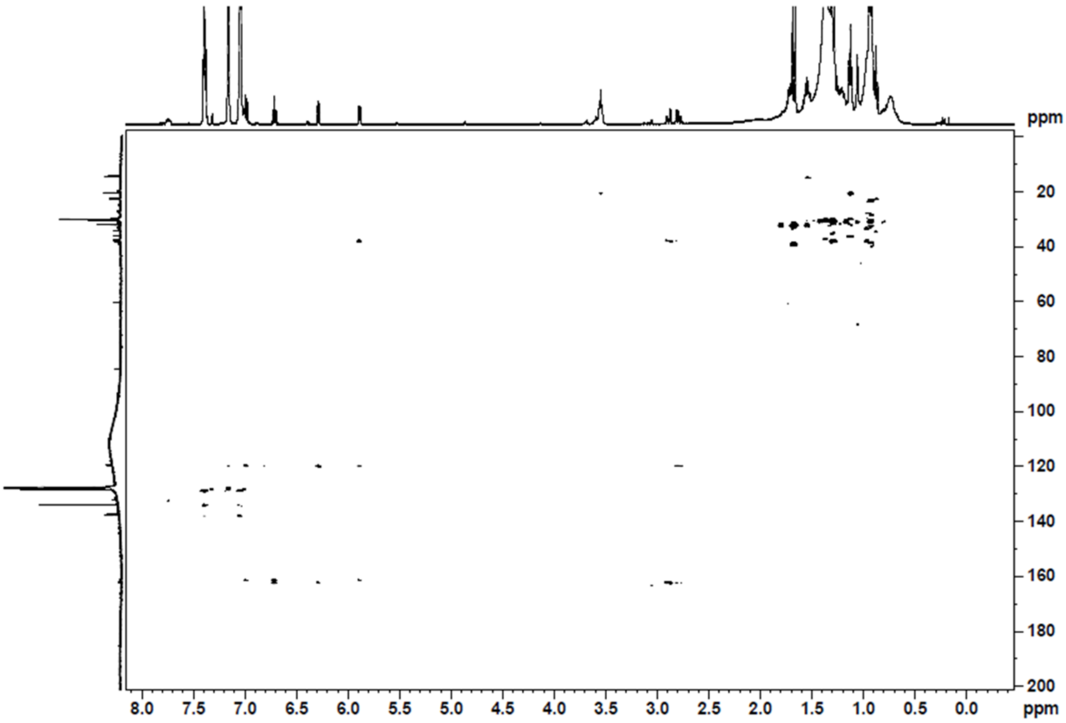

Figure S29: ^{1}H-^{13}C HMBC NMR spectrum of [Tc(^{Pyr}PNP^{tBu}–CSS)$(CO)_2$] (**6**).

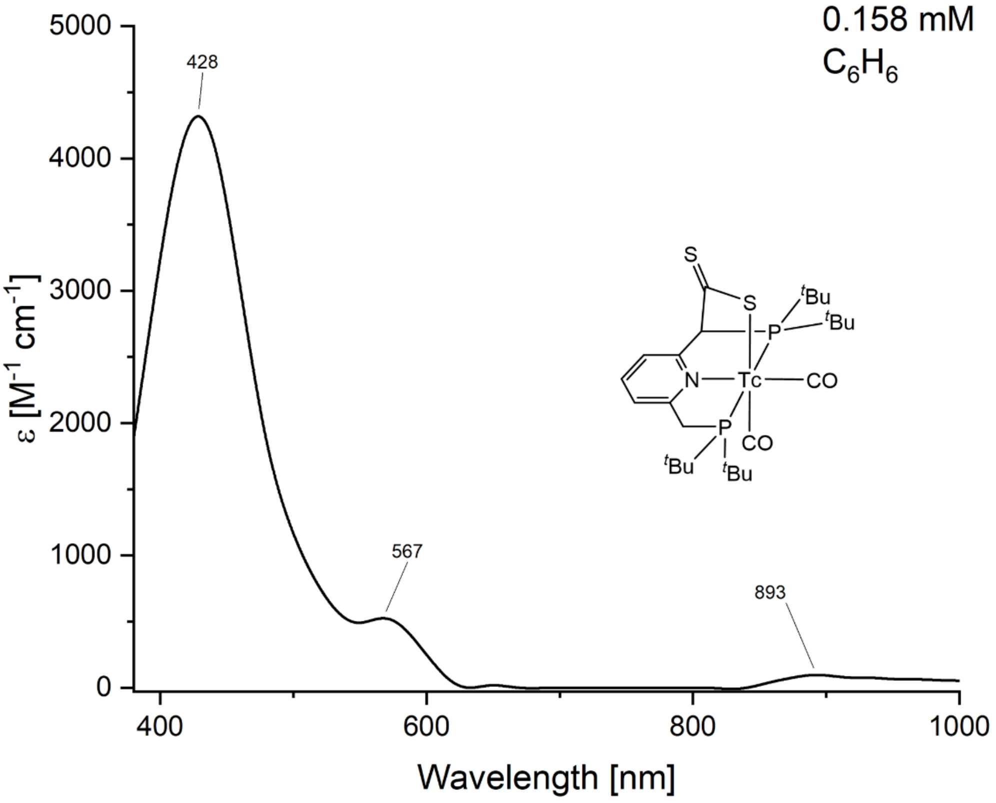

Figure S30: VIS spectrum (C_6H_6) of [Tc(^{Pyr}PNP^{tBu}–CSS)$(CO)_2$] (**6**).

4.5 [Tc(^{Pyr}PNP^{tBu})(CO)$_2$H] (7)

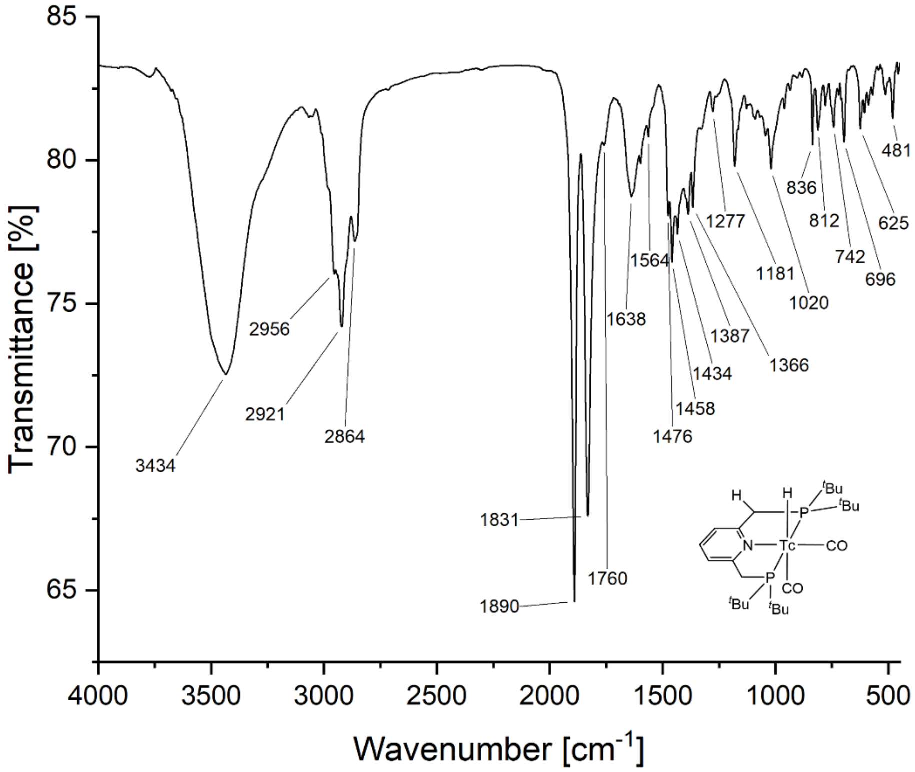

Figure S31: IR spectrum (KBr) of [Tc(^{Pyr}PNP^{tBu})(CO)$_2$H] (**7**).

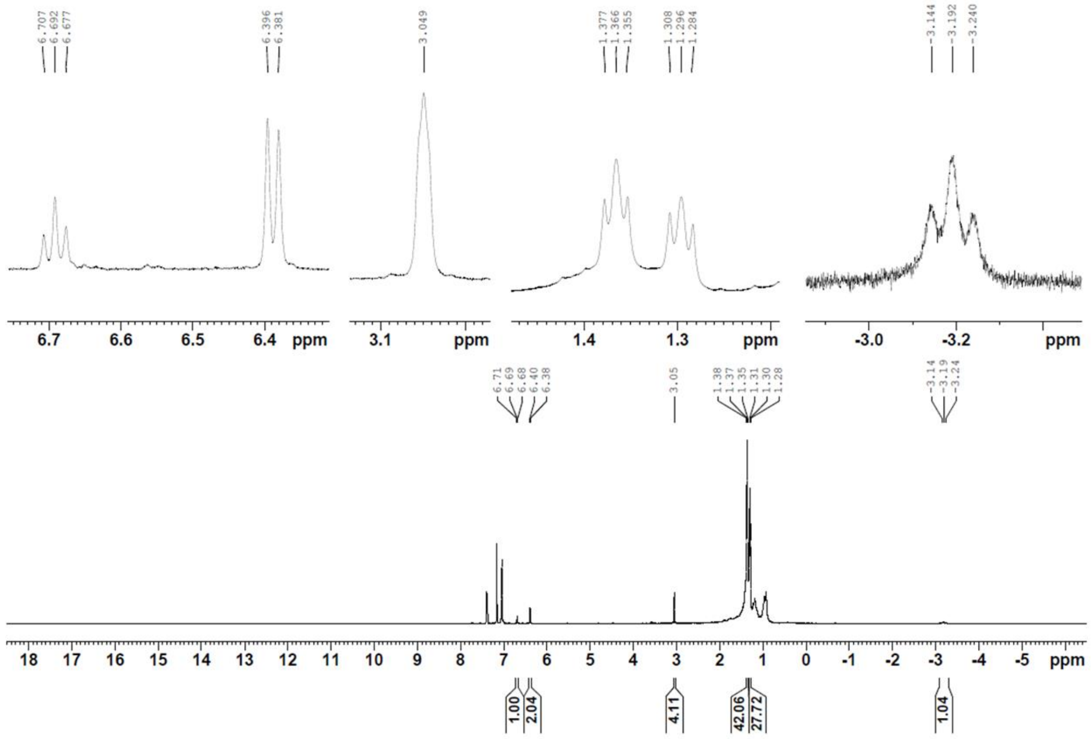

Figure S32: ^{1}H NMR spectrum of [Tc(^{Pyr}PNP^{tBu})(CO)$_2$H] (**7**).

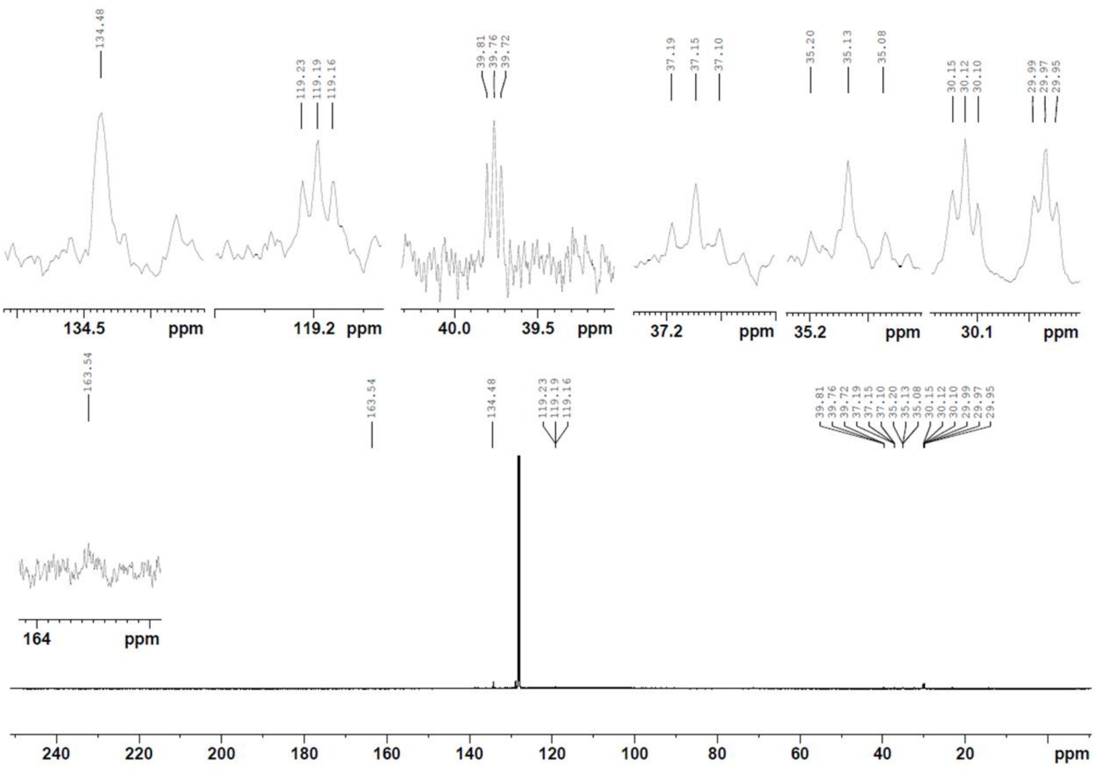

Figure S33: $^{13}C\{^{1}H\}$ NMR spectrum of [Tc(^{Pyr}PNP^{tBu})(CO)$_2$H] (**7**).

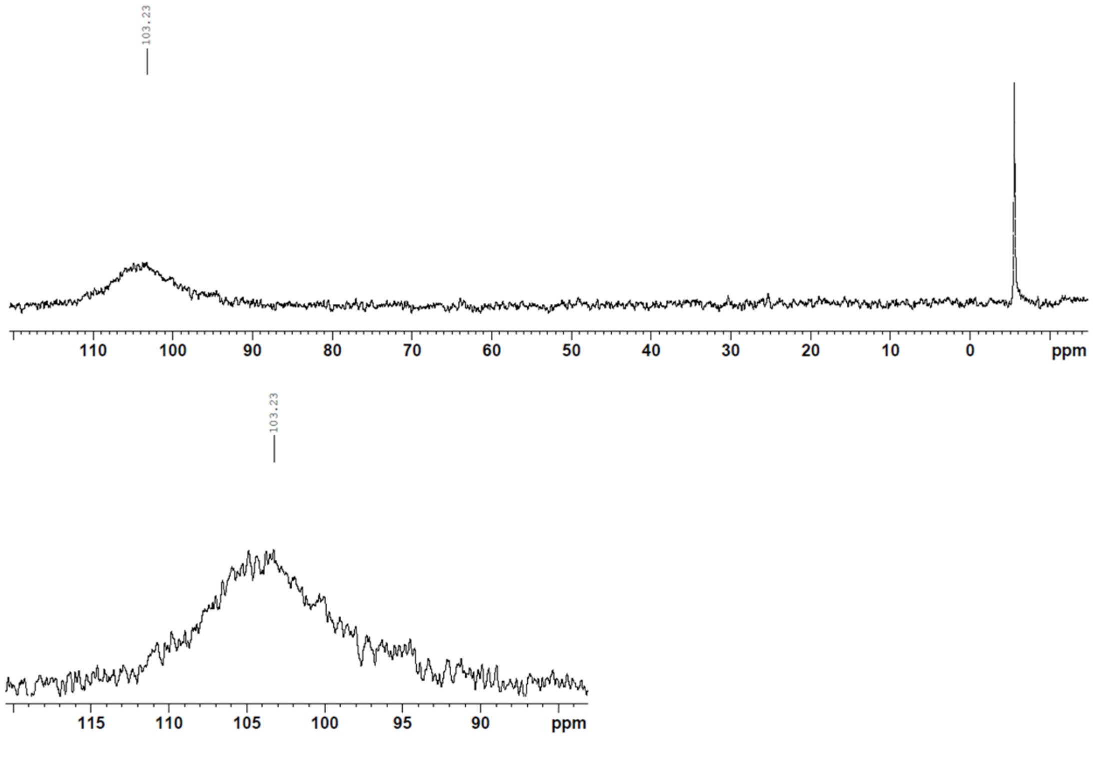

Figure S34: ^{31}P NMR spectrum of [Tc(^{Pyr}PNP^{tBu})(CO)$_2$H] (**7**).

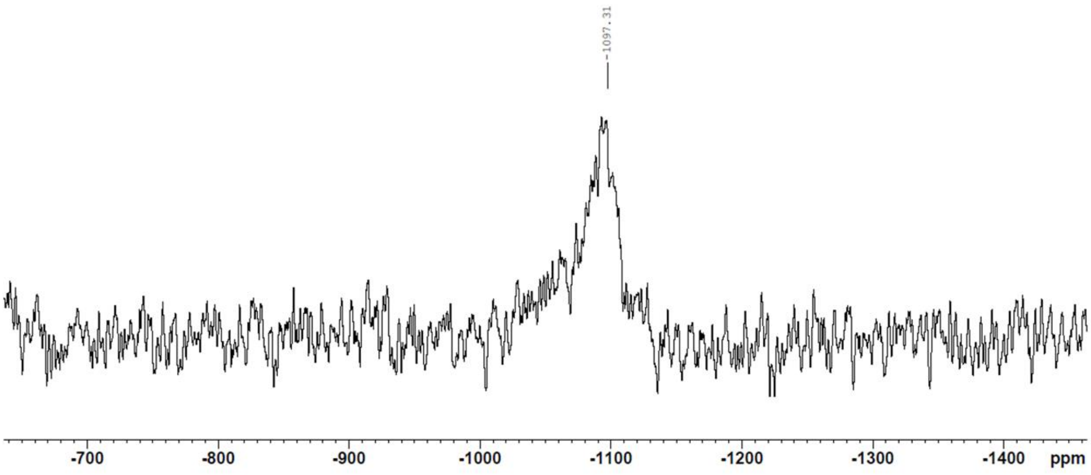

Figure S35: ^{99}Tc NMR spectrum of [Tc(^{Pyr}PNP^{tBu})(CO)$_2$H] (**7**).

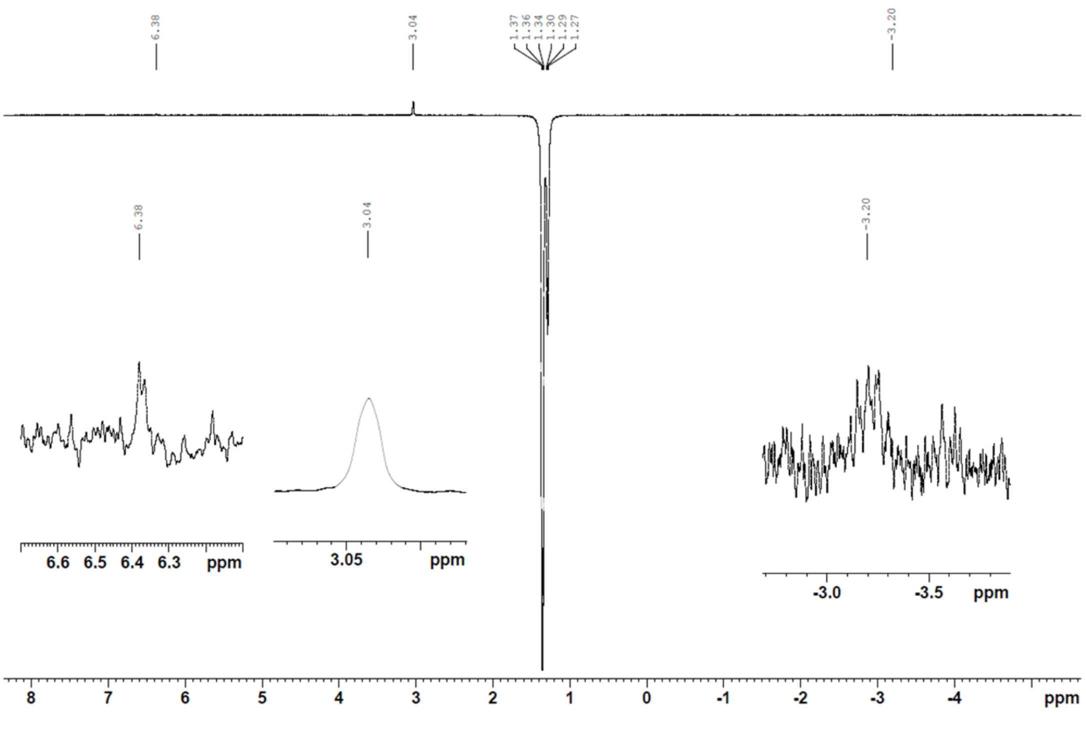

Figure S36: 1D-NOE ^{1}H NMR spectrum of [Tc(^{Pyr}PNP^{tBu})(CO)$_2$H] (**7**), irradiated on 1.35 ppm (^{t}Bu signal).

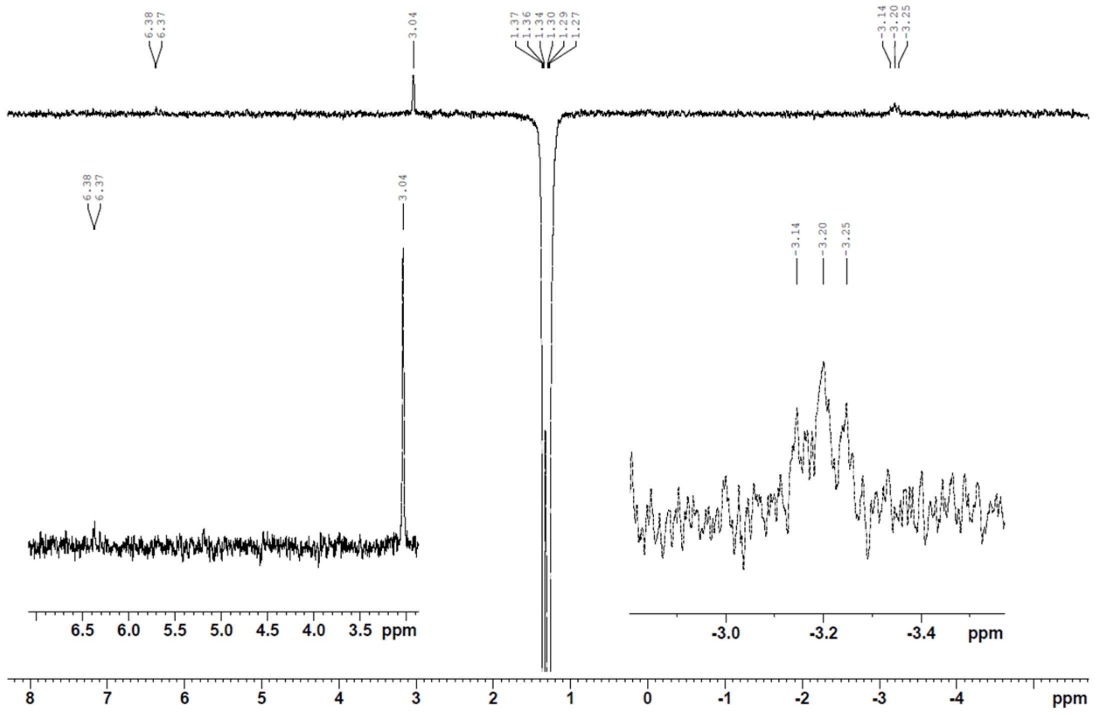

Figure S37: 1D-NOE ^{1}H NMR spectrum of [Tc(^{Pyr}PNP^{tBu})(CO)$_2$H] (**7**), irradiated on 1.29 ppm (^{t}Bu signal).

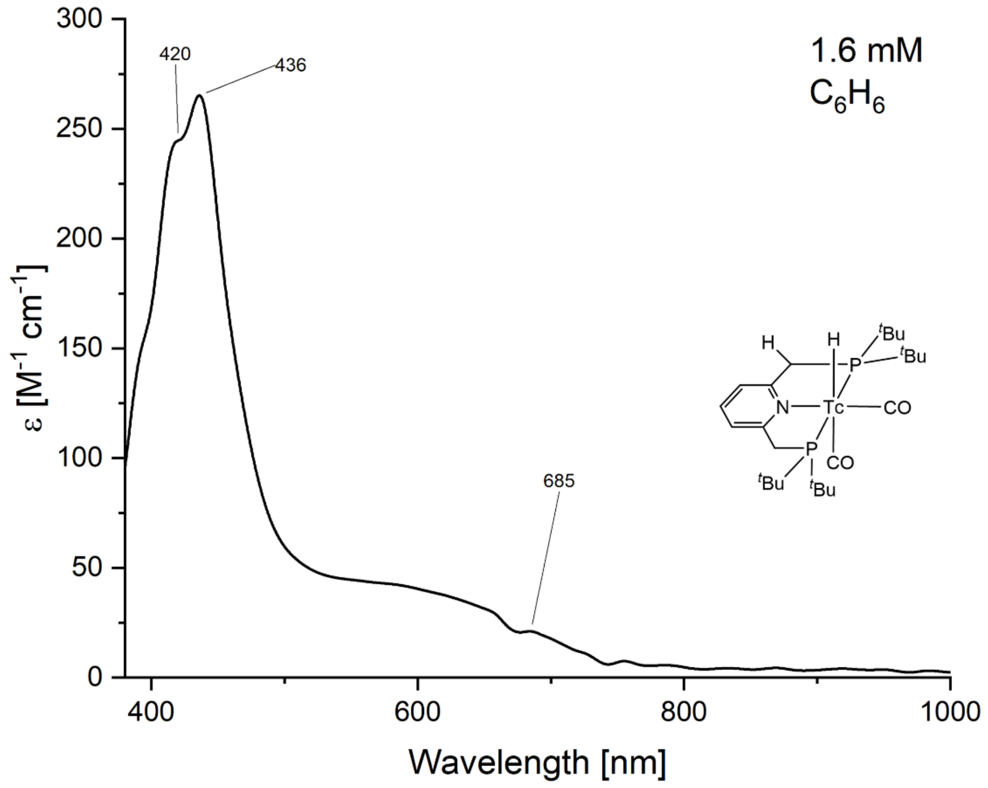

Figure S38: VIS spectrum (C_6H_6) of [Tc(^{Pyr}PNP^{tBu})(CO)$_2$H] (**7**).

4.6 [Tc(^{Pyr}PNP^{tBu})(CO)$_2$(–C≡C–Ph)] (8)

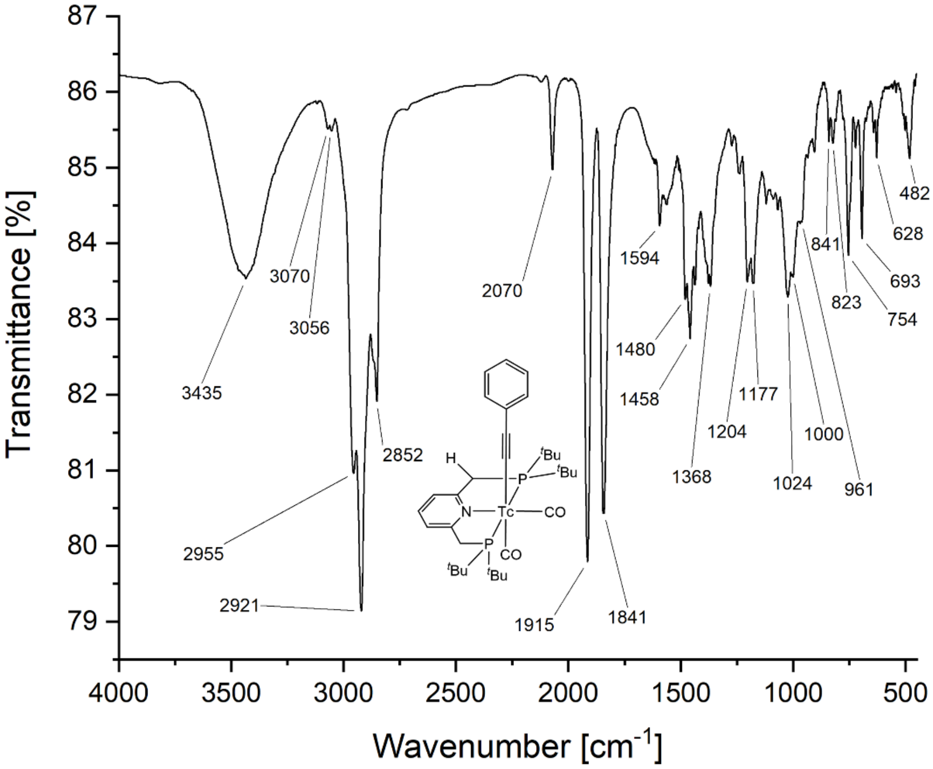

Figure S39: IR spectrum (KBr) of [Tc(^{Pyr}PNP^{tBu})(CO)$_2$(–C≡C–Ph)] (**8**).

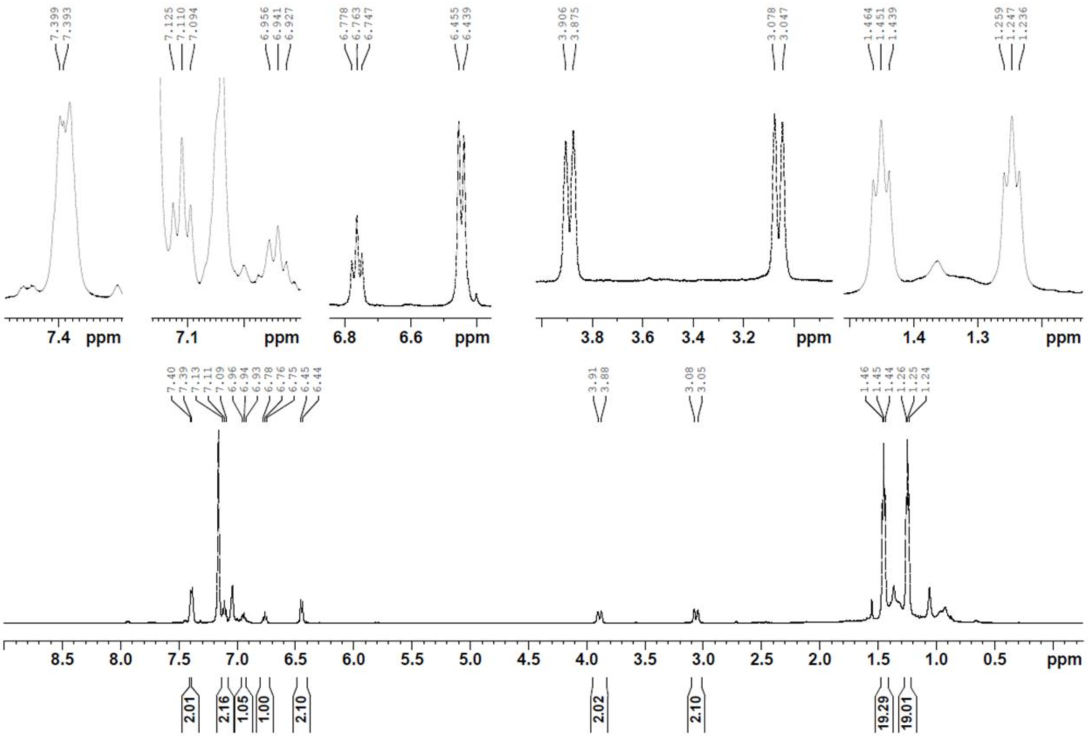

Figure S40: ^{1}H NMR spectrum of [Tc(^{Pyr}PNP^{tBu})(CO)$_2$(–C≡C–Ph)] (**8**).

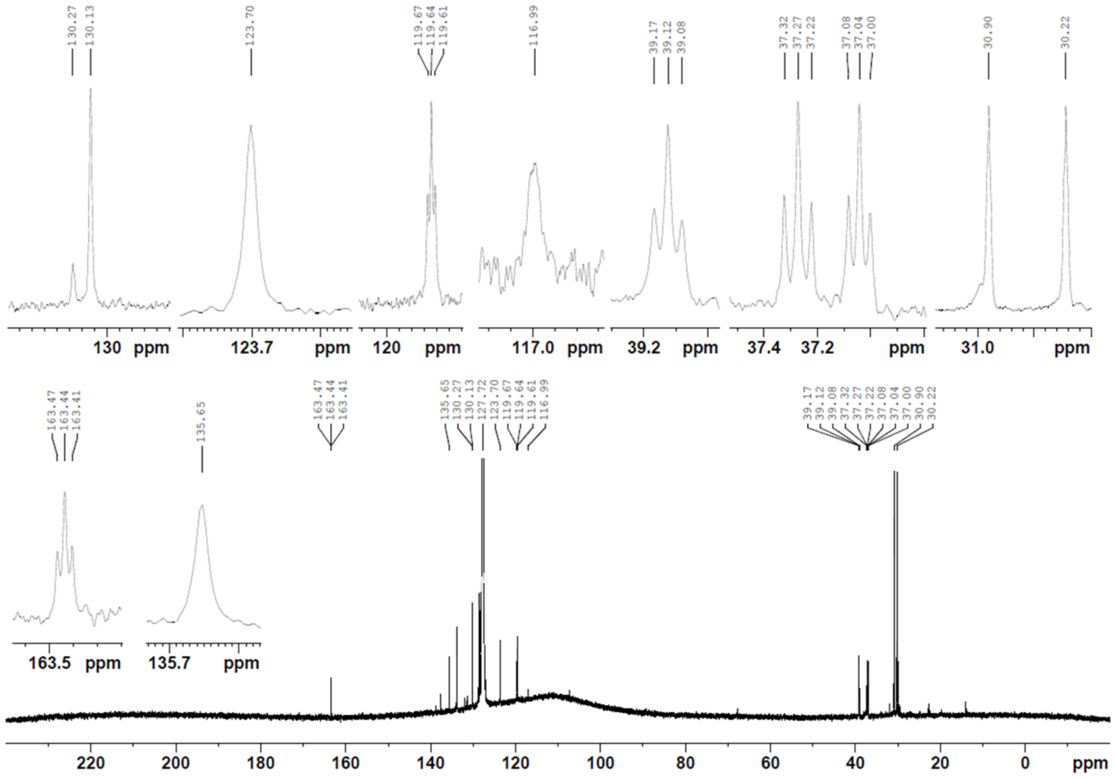

Figure S41: $^{13}C\{^{1}H\}$ NMR spectrum of [Tc(^{Pyr}PNP^{tBu})(CO)$_2$(–C≡C–Ph)] (**8**).

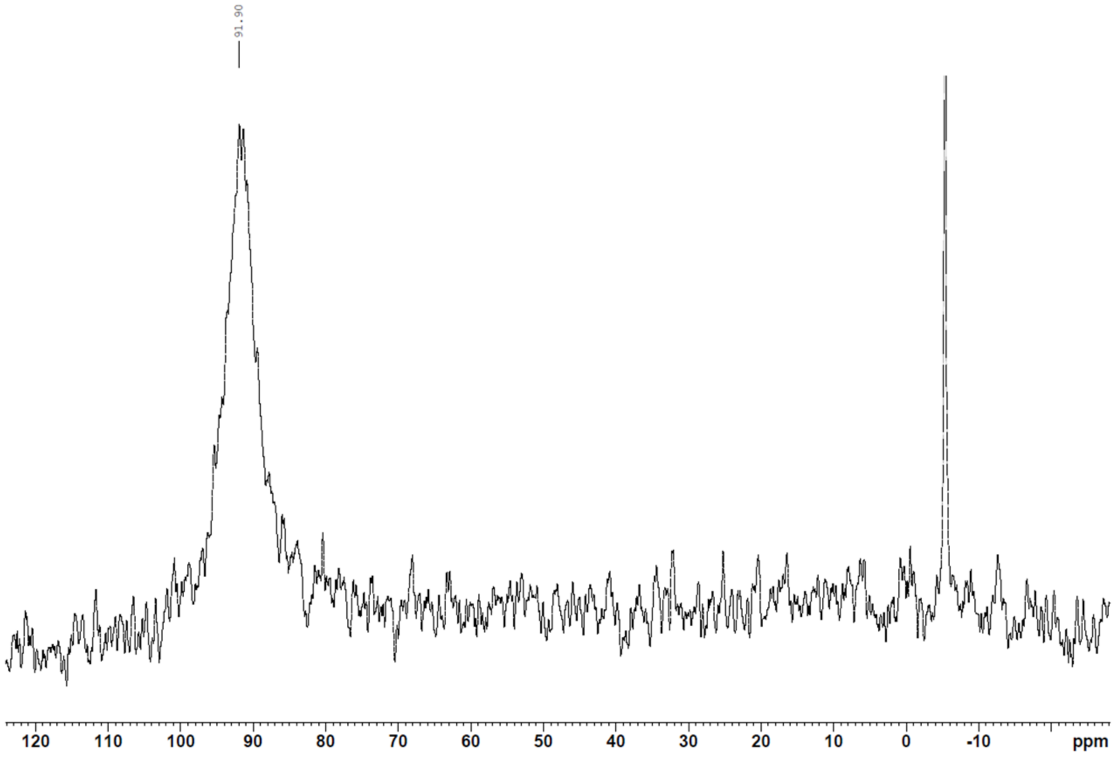

Figure S42: $^{31}P\{^{1}H\}$ NMR spectrum of [Tc(^{Pyr}PNP^{tBu})(CO)$_2$(–C≡C–Ph)] (**8**).

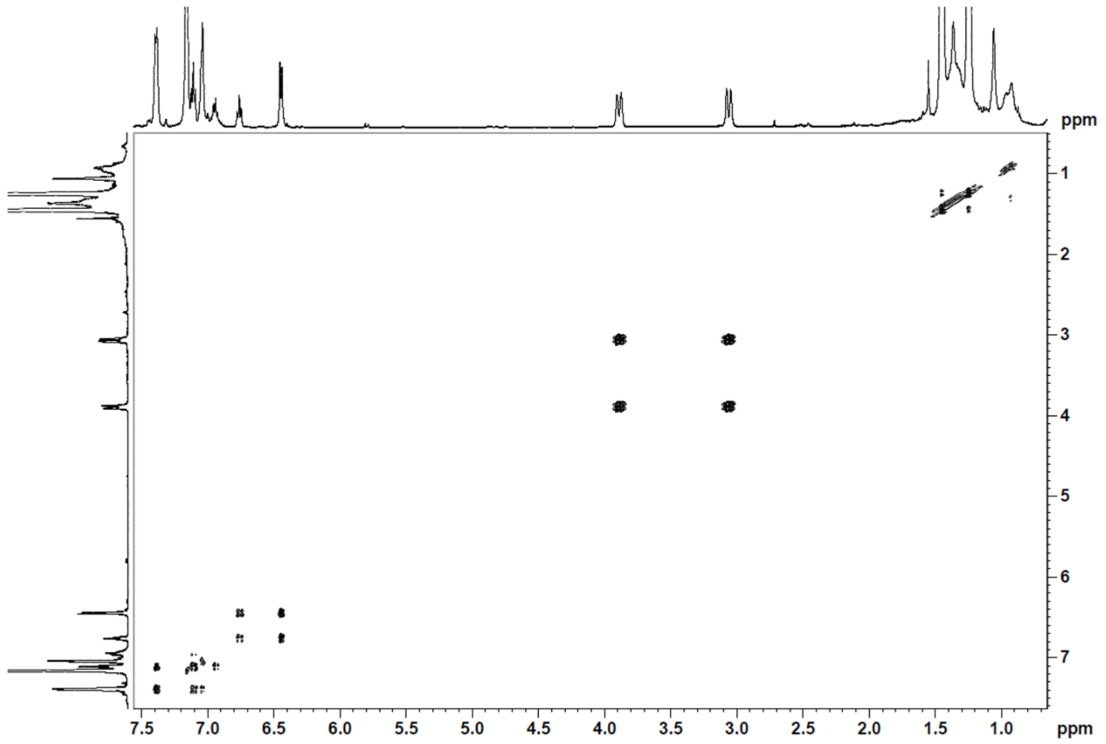

Figure S43: ^{1}H-^{1}H COSY NMR spectrum of [Tc($^{Pyr}PNP^{tBu}$)(CO)$_2$(–C≡C–Ph)] (**8**).

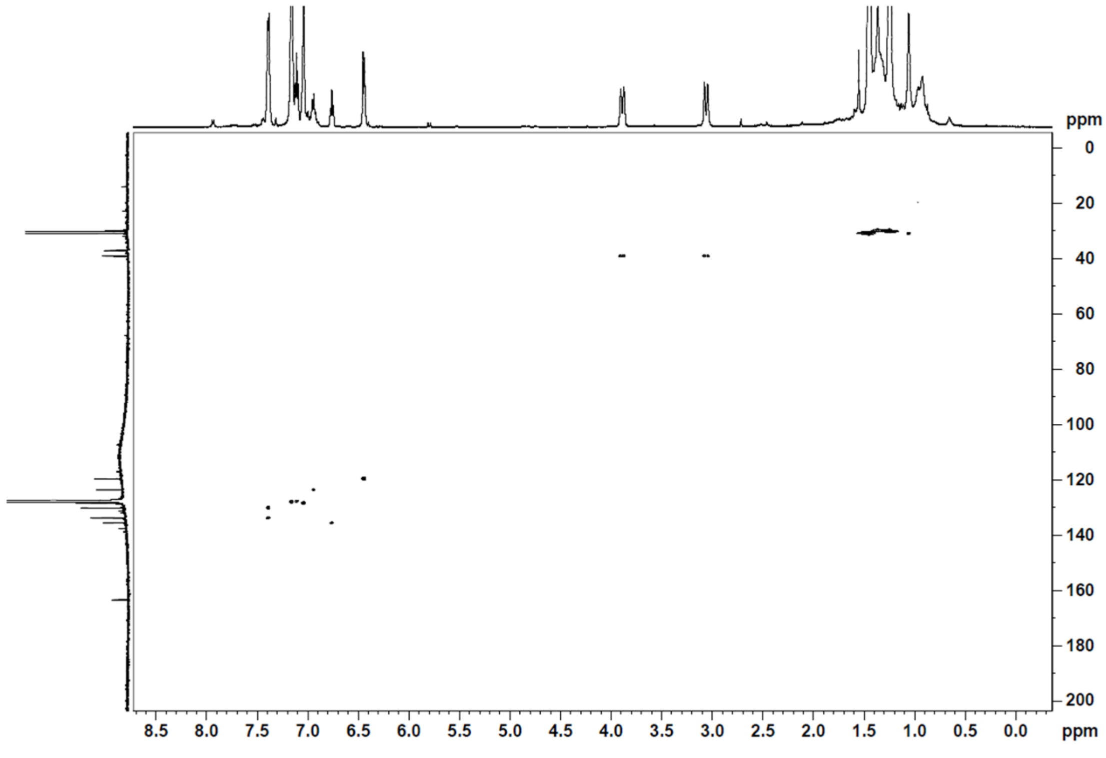

Figure S44: ^{1}H-^{13}C HSQC NMR spectrum of [Tc($^{Pyr}PNP^{tBu}$)(CO)$_2$(–C≡C–Ph)] (**8**).

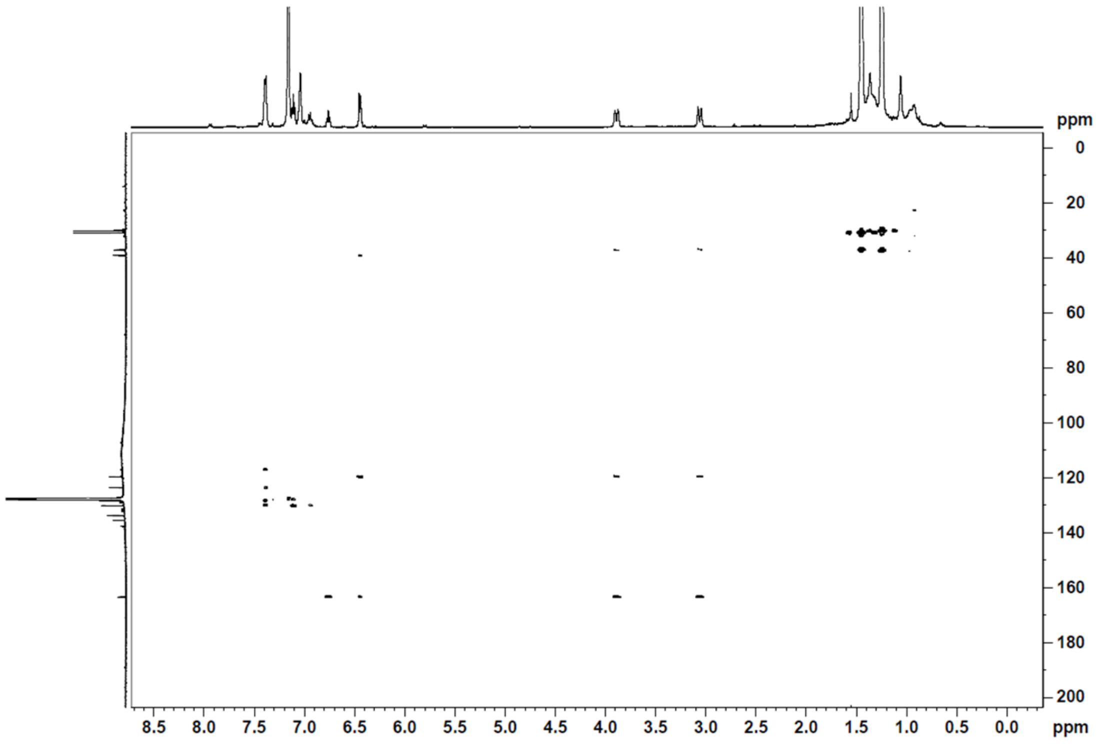

Figure S45: ^{1}H-^{13}C HMBC NMR spectrum of [Tc($^{Pyr}PNP^{tBu}$)(CO)$_2$(–C≡C–Ph)] (**8**).

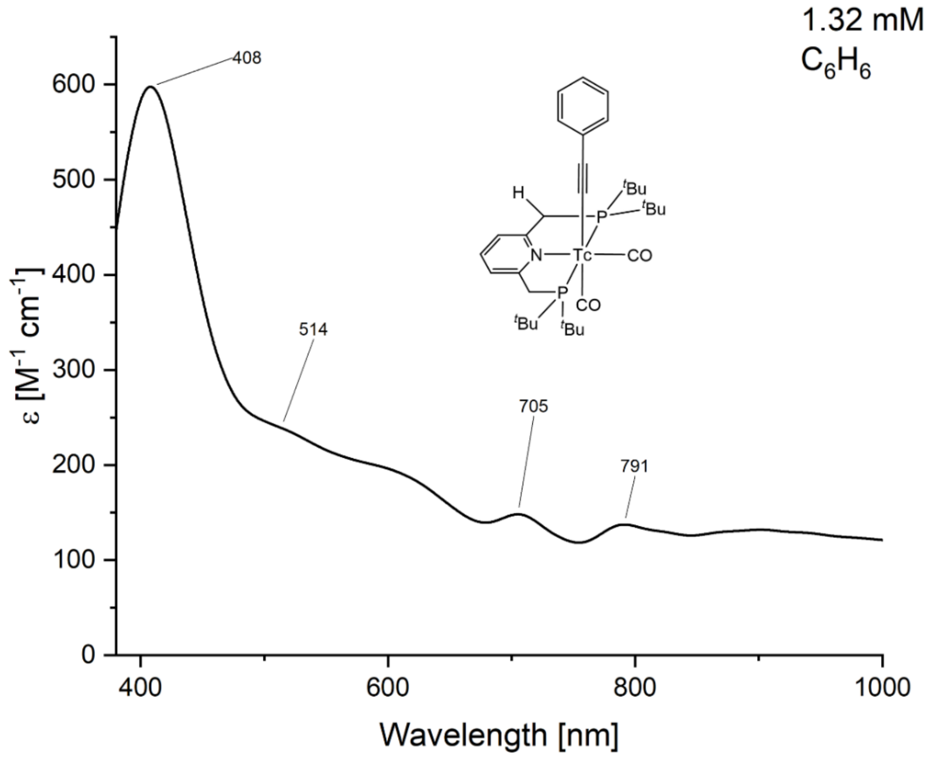

Figure S46: VIS spectrum (C_6H_6) of [Tc($^{Pyr}PNP^{tBu}$)(CO)$_2$(–C≡C–Ph)] (**8**).

4.7 [Tc(^{Pyr}PNP^{tBu})(CO)$_2$(–C≡C–TMS)] (9)

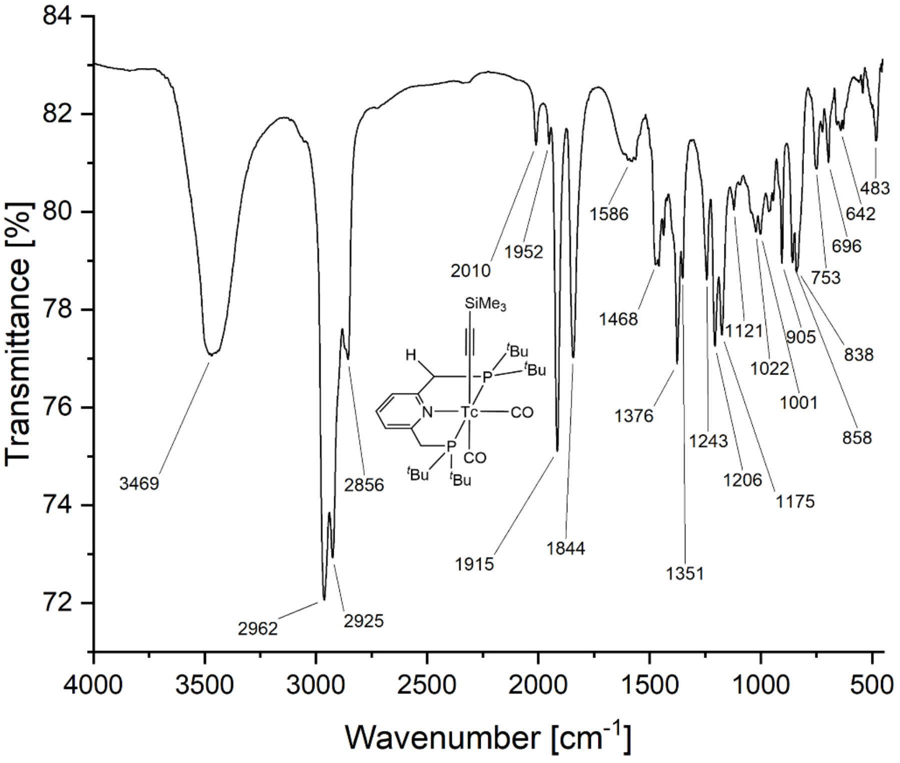

Figure S47: IR spectrum (KBr) of [Tc(^{Pyr}PNP^{tBu})(CO)$_2$(–C≡C–TMS)] (**9**).

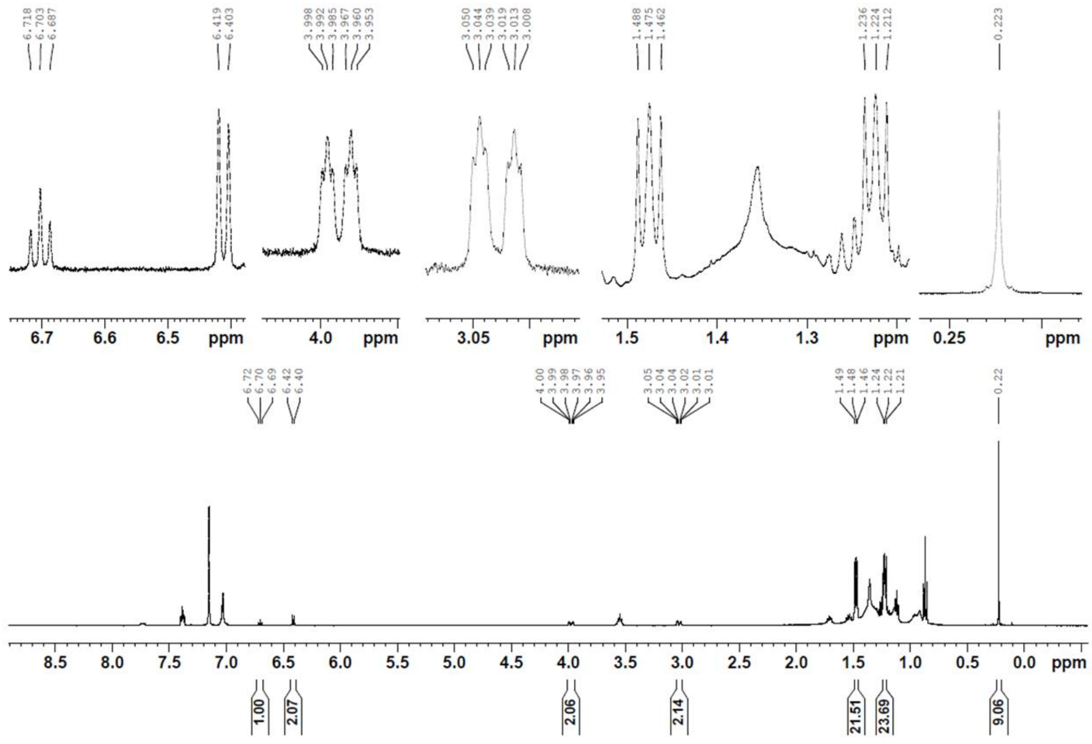

Figure S48: ^{1}H NMR spectrum of [Tc(^{Pyr}PNP^{tBu})(CO)$_2$(–C≡C–TMS)] (**9**).

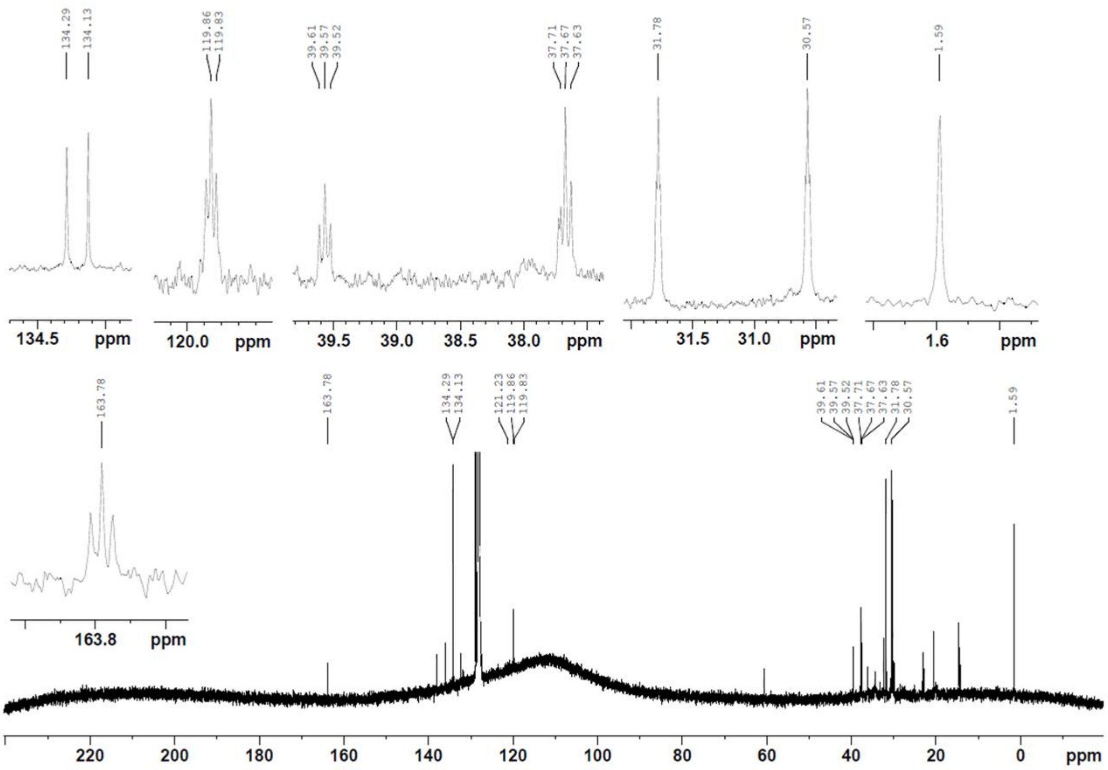

Figure S49: $^{13}C\{^{1}H\}$ NMR spectrum of [Tc(^{Pyr}PNP^{tBu})(CO)$_2$(–C≡C–TMS)] (**9**).

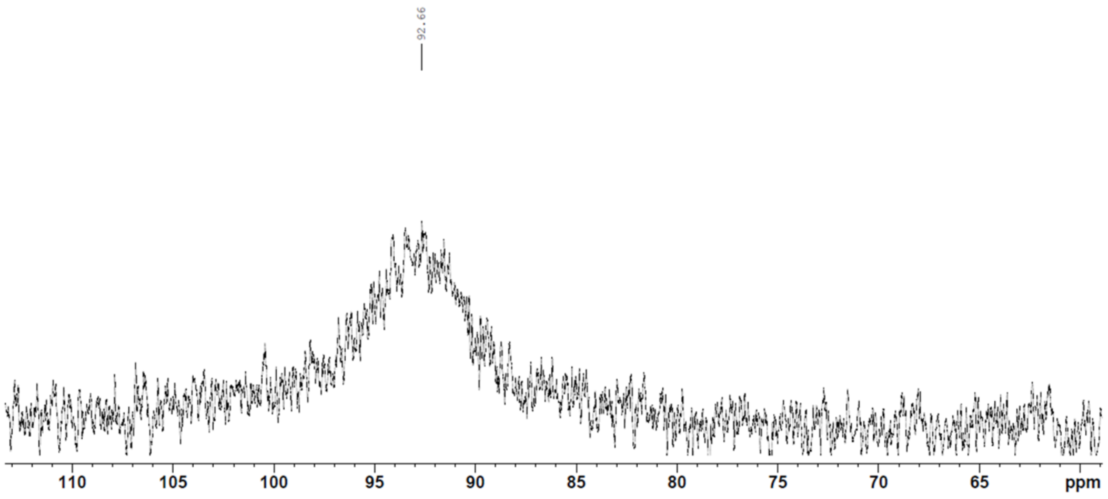

Figure S50: $^{31}P\{^{1}H\}$ NMR spectrum of [Tc(^{Pyr}PNP^{tBu})(CO)$_2$(–C≡C–TMS)] (**9**).

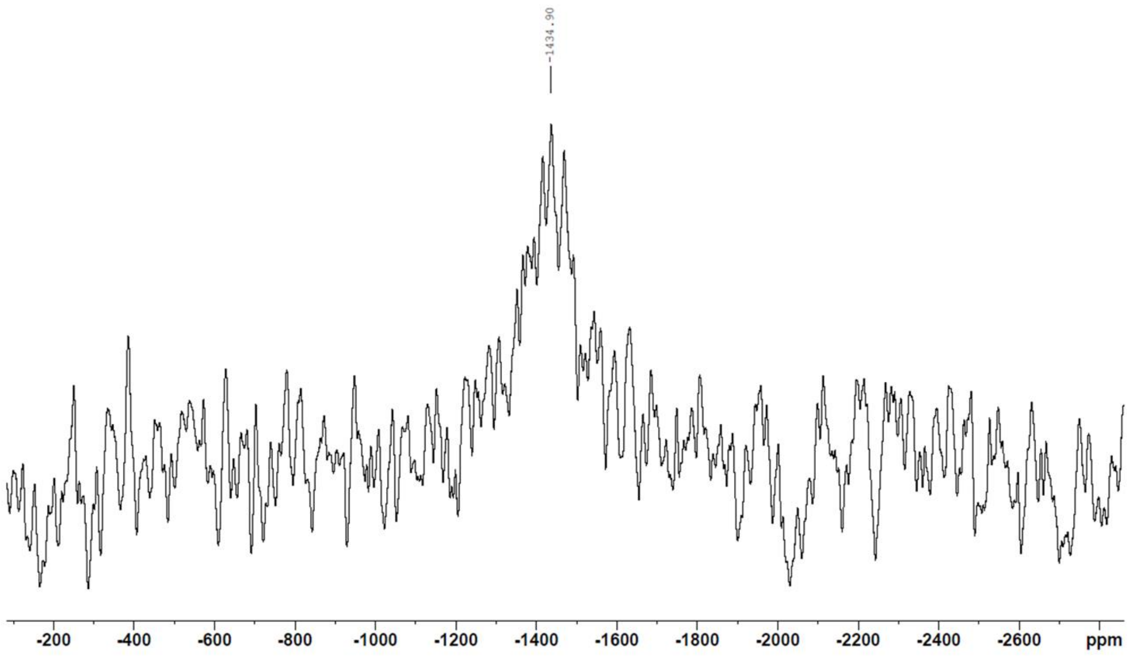

Figure S51: ^{99}Tc NMR spectrum of [Tc(^{Pyr}PNP^{tBu})(CO)$_2$(–C≡C–TMS)] (**9**).

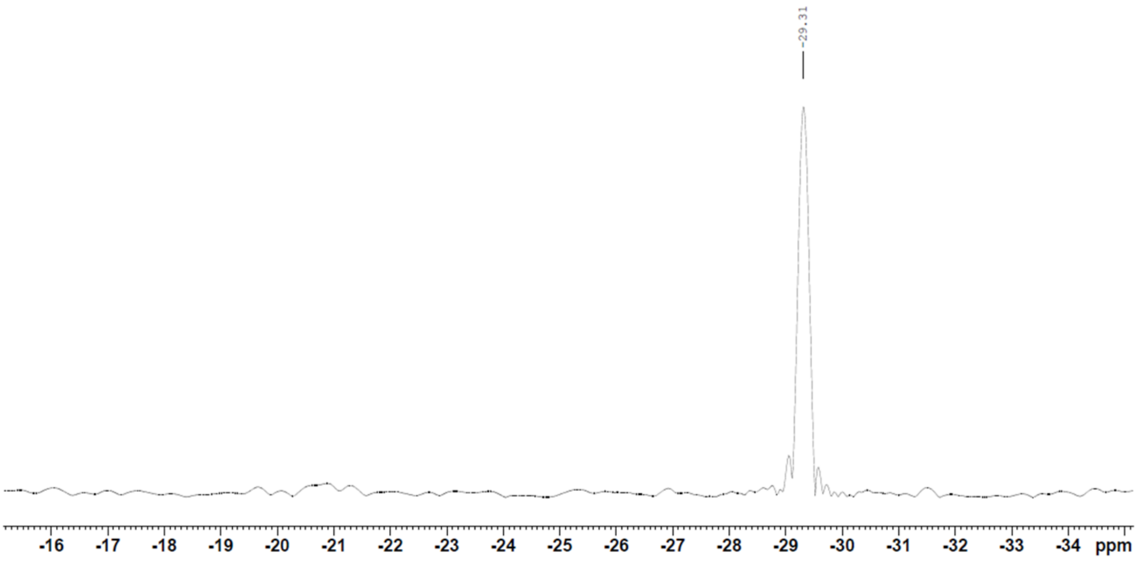

Figure S52: ^{29}Si NMR spectrum of [Tc(^{Pyr}PNP^{tBu})(CO)$_2$(–C≡C–TMS)] (**9**); (1D spectrum extracted from ^{29}Si-^{1}H HMBC).

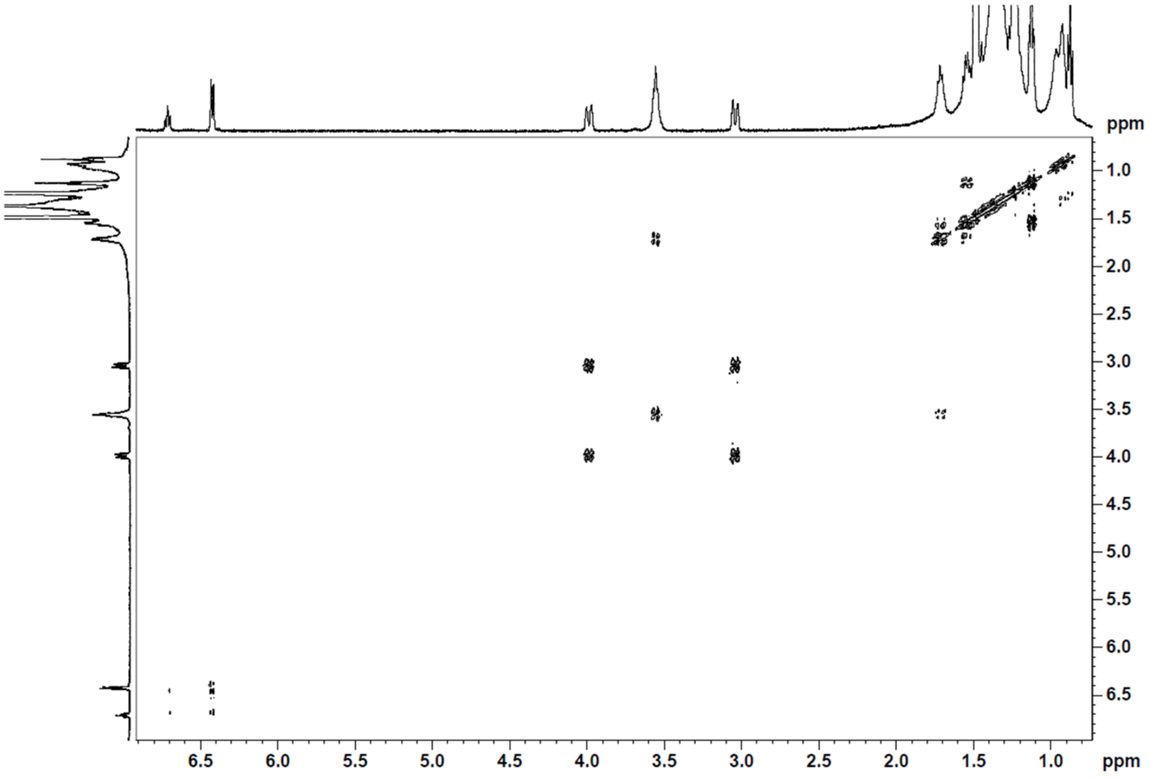

Figure S53: ^{1}H-^{1}H COSY NMR spectrum of [Tc($^{Pyr}PNP^{tBu}$)(CO)$_2$(–C≡C–TMS)] (**9**).

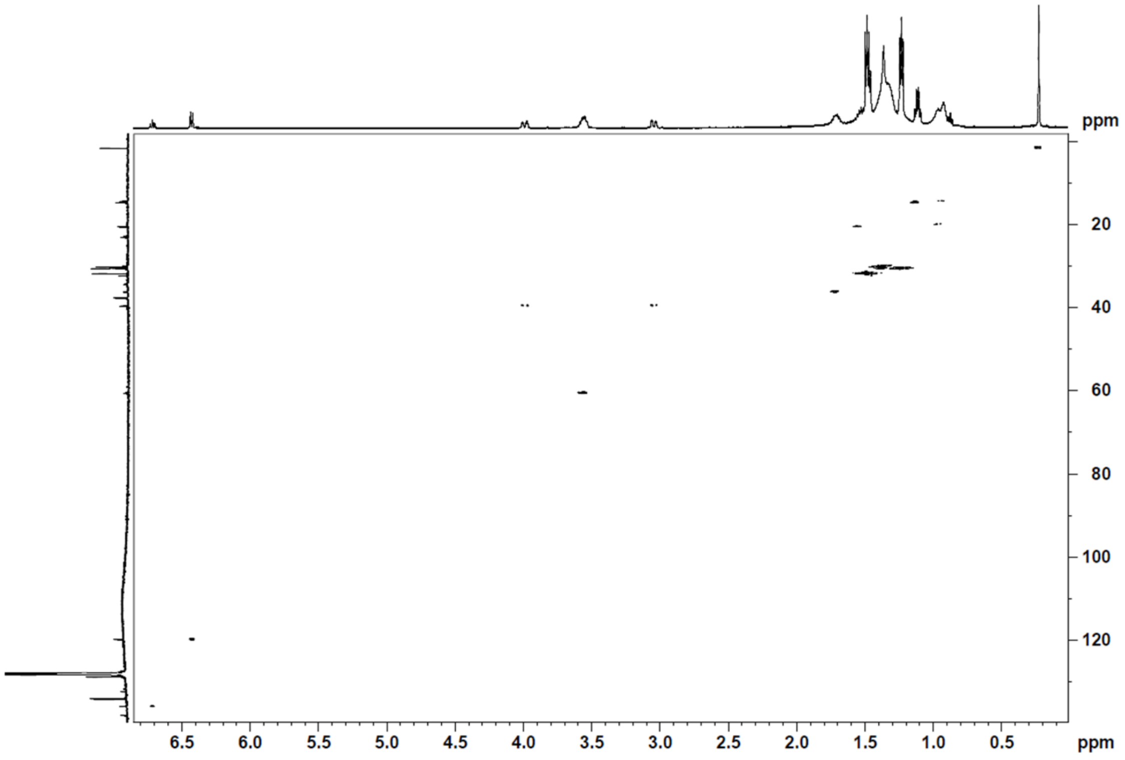

Figure S54: ^{1}H-^{13}C HSQC NMR spectrum of [Tc($^{Pyr}PNP^{tBu}$)(CO)$_2$(–C≡C–TMS)] (**9**).

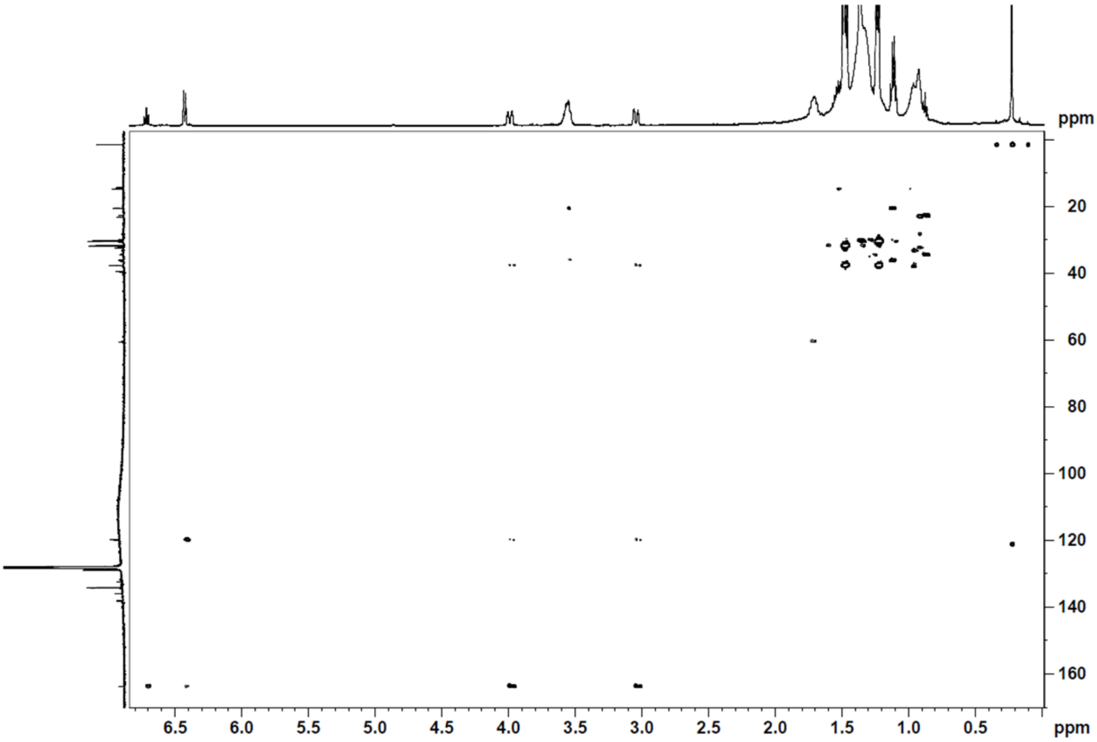

Figure S55: ^{1}H-^{13}C HMBC NMR spectrum of [Tc(^{Pyr}PNP^{tBu})(CO)$_2$(–C≡C–TMS)] (**9**).

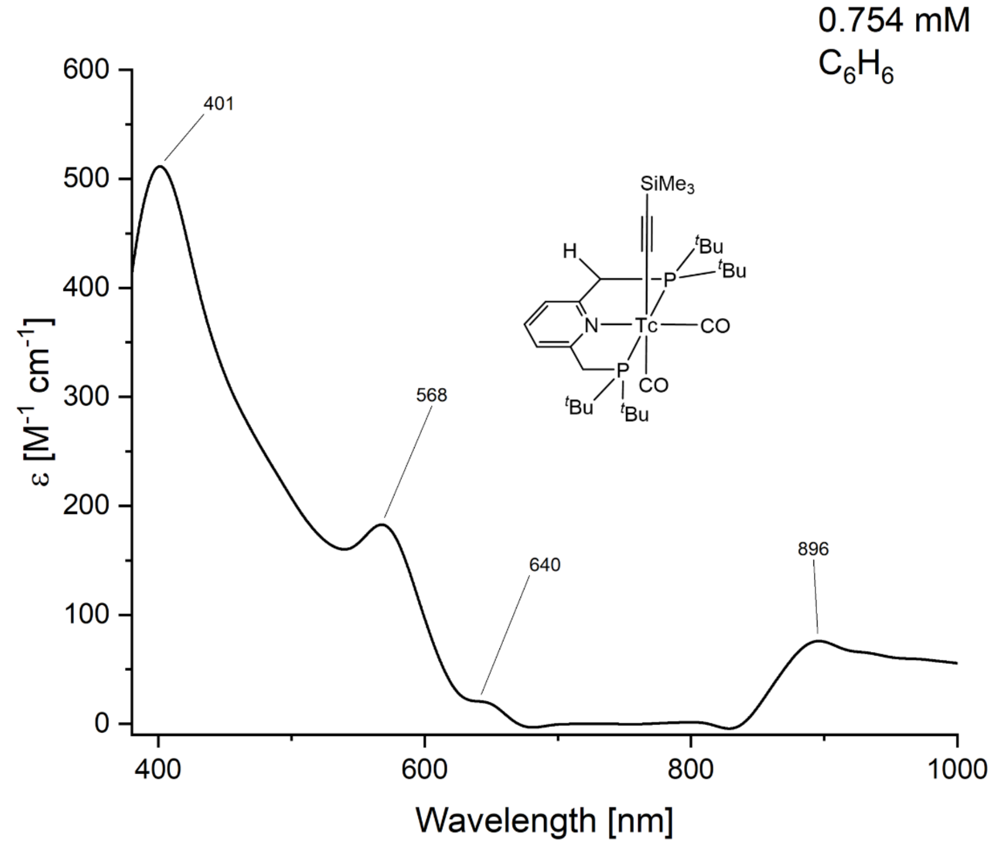

Figure S56: VIS spectrum (C_6H_6) of [Tc(^{Pyr}PNP^{tBu})(CO)$_2$(–C≡C–TMS)] (**9**).

4.8 [Tc(^{Pyr}PNP^{tBu})(CO)$_2$(DMIM)] (10)

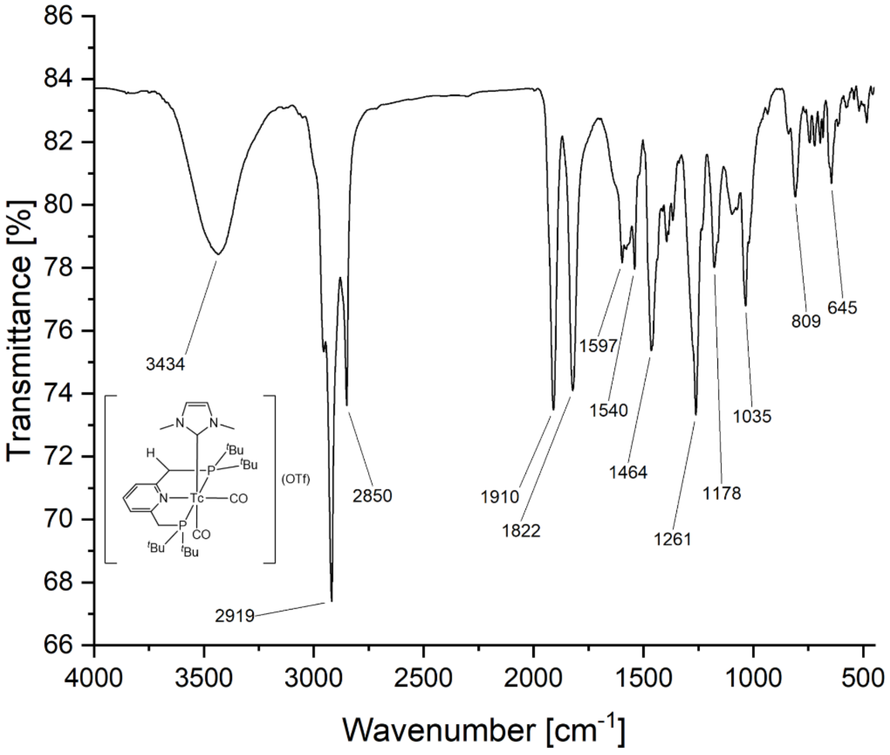

Figure S57: IR spectrum (KBr) of [Tc(^{Pyr}PNP^{tBu})(CO)$_2$(DMIM)] (**10**).

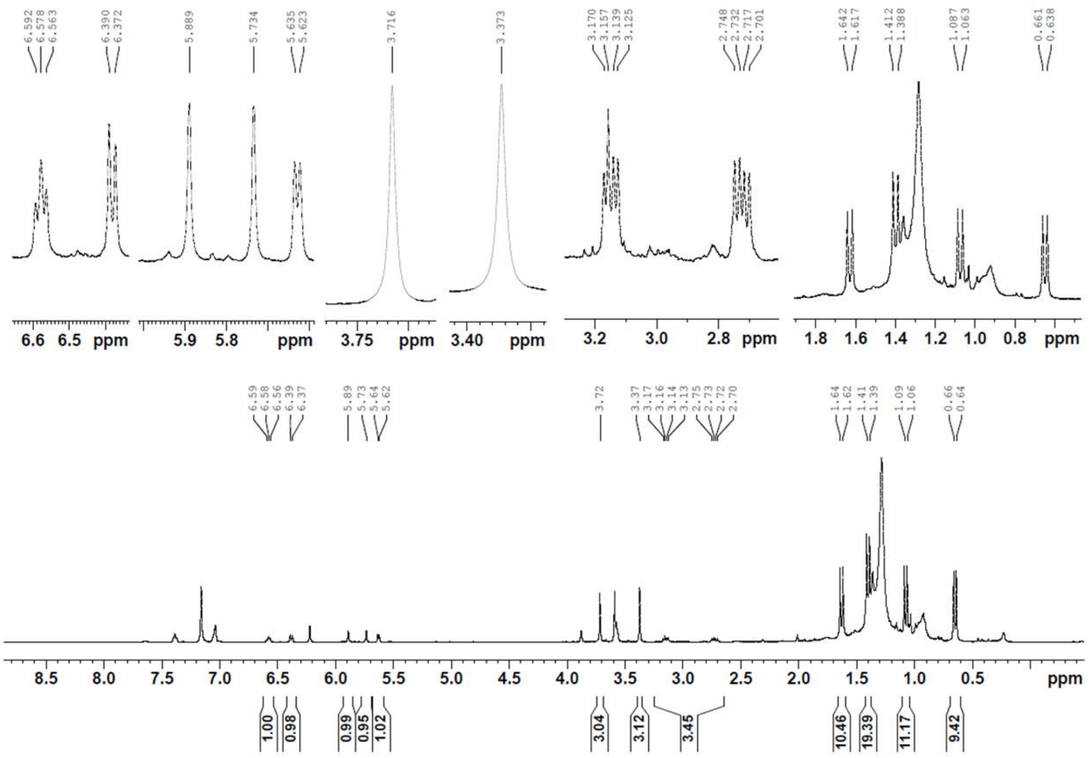

Figure S58: ^{1}H NMR spectrum of [Tc(^{Pyr}PNP^{tBu})(CO)$_2$(DMIM)] (**10**).

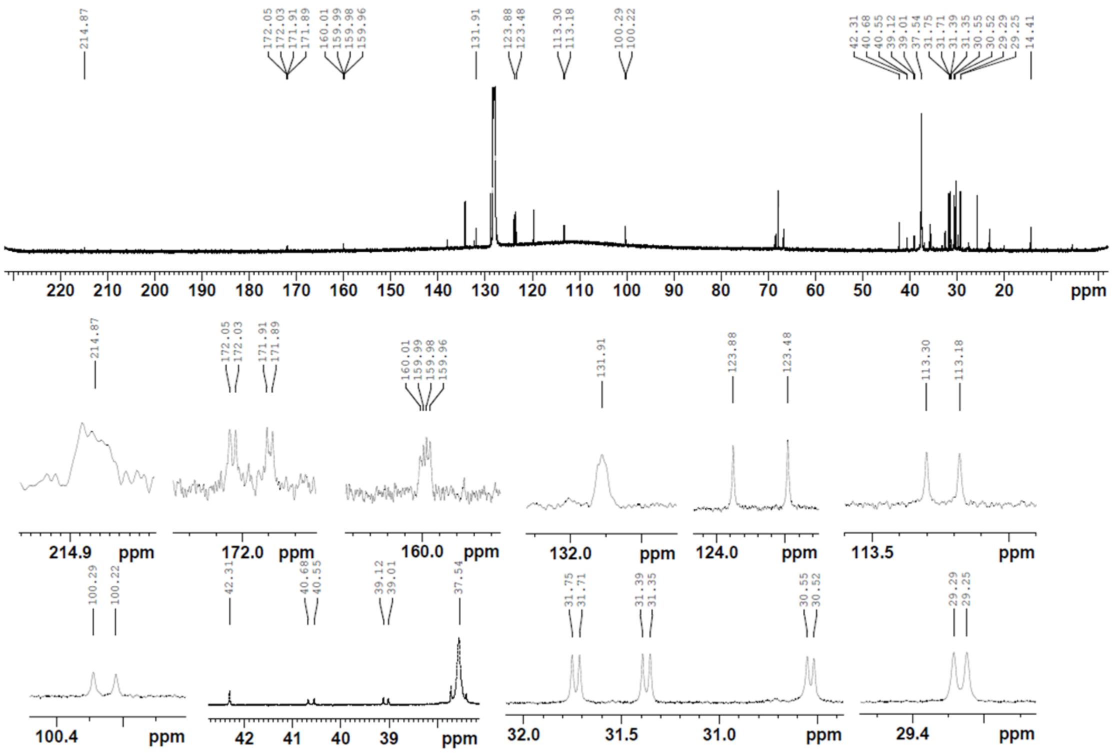

Figure S59: $^{13}C\{^{1}H\}$ NMR spectrum of [Tc(^{Pyr}PNP^{tBu})$(CO)_2$(DMIM)] (**10**).

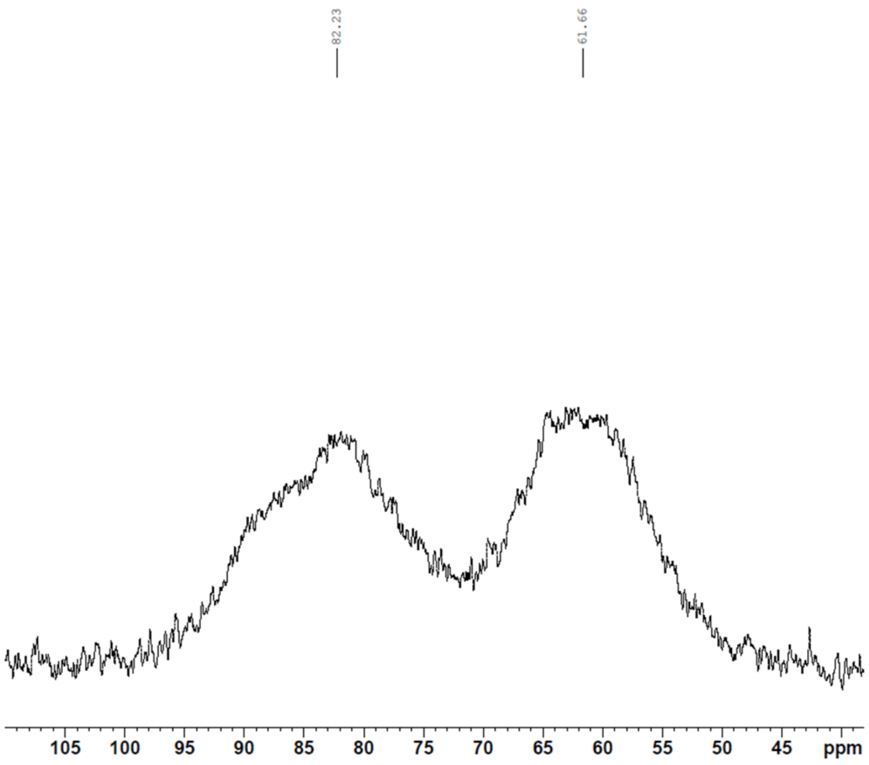

Figure S60: $^{31}P\{^{1}H\}$ NMR spectrum of [Tc(^{Pyr}PNP^{tBu})$(CO)_2$(DMIM)] (**10**).

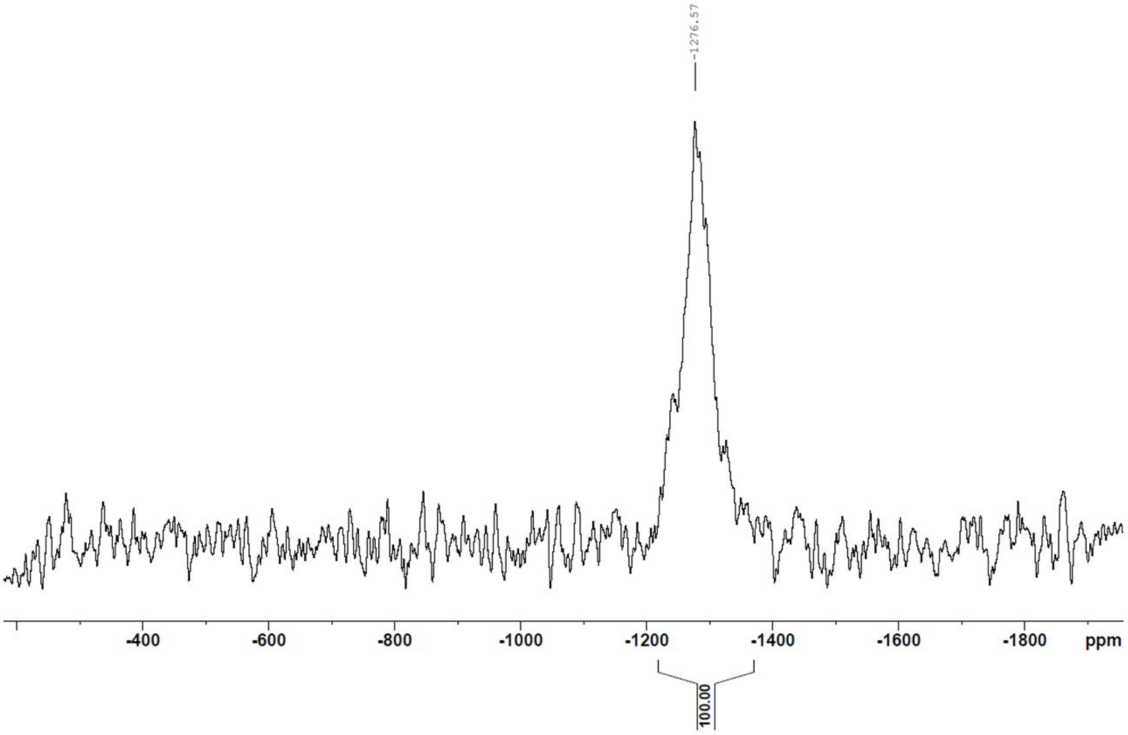

Figure S61: ^{99}Tc NMR spectrum of [Tc(^{Pyr}PNP^{tBu})(CO)$_2$(DMIM)] (**10**).

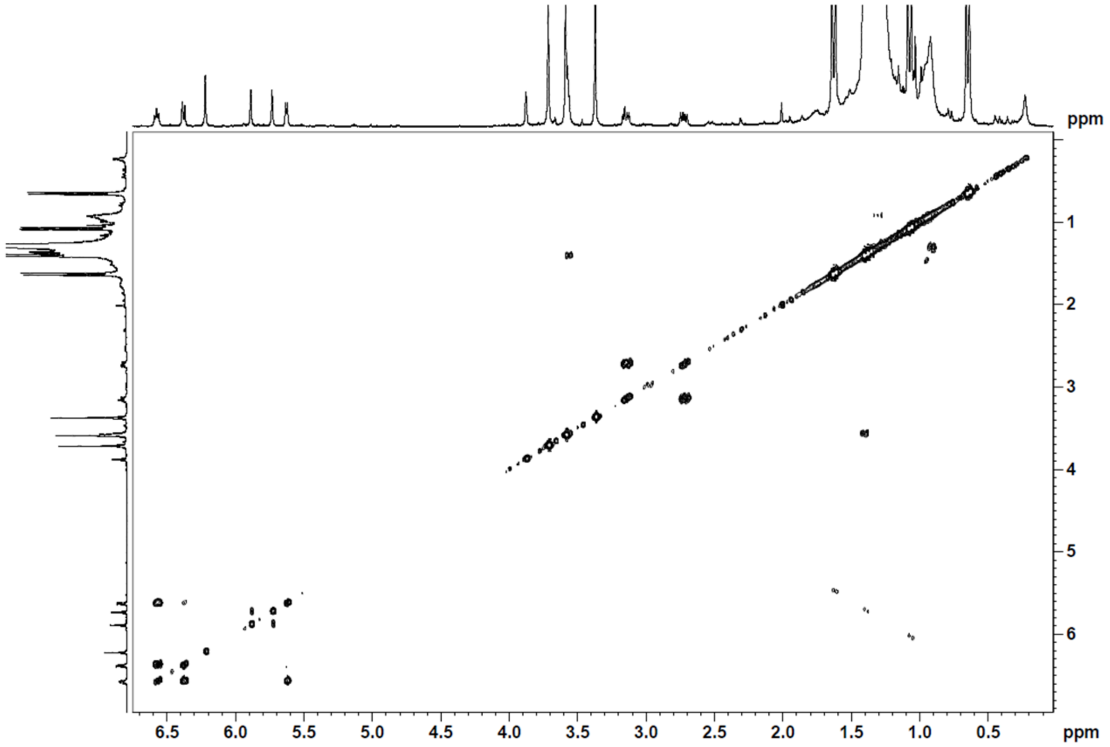

Figure S62: ^{1}H-^{1}H COSY NMR spectrum of [Tc(^{Pyr}PNP^{tBu})(CO)$_2$(DMIM)] (**10**).

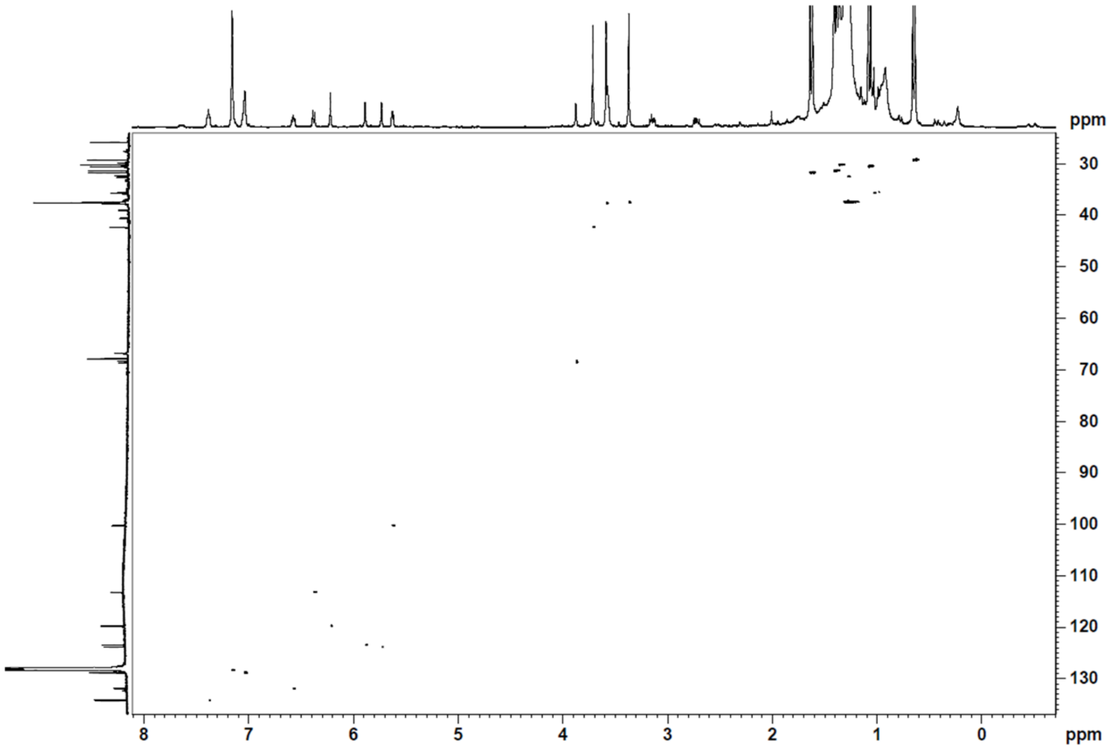

Figure S63: ^{1}H-^{13}C HSQC NMR spectrum of [Tc(^{Pyr}PNP^{tBu})(CO)$_2$(DMIM)] (**10**).

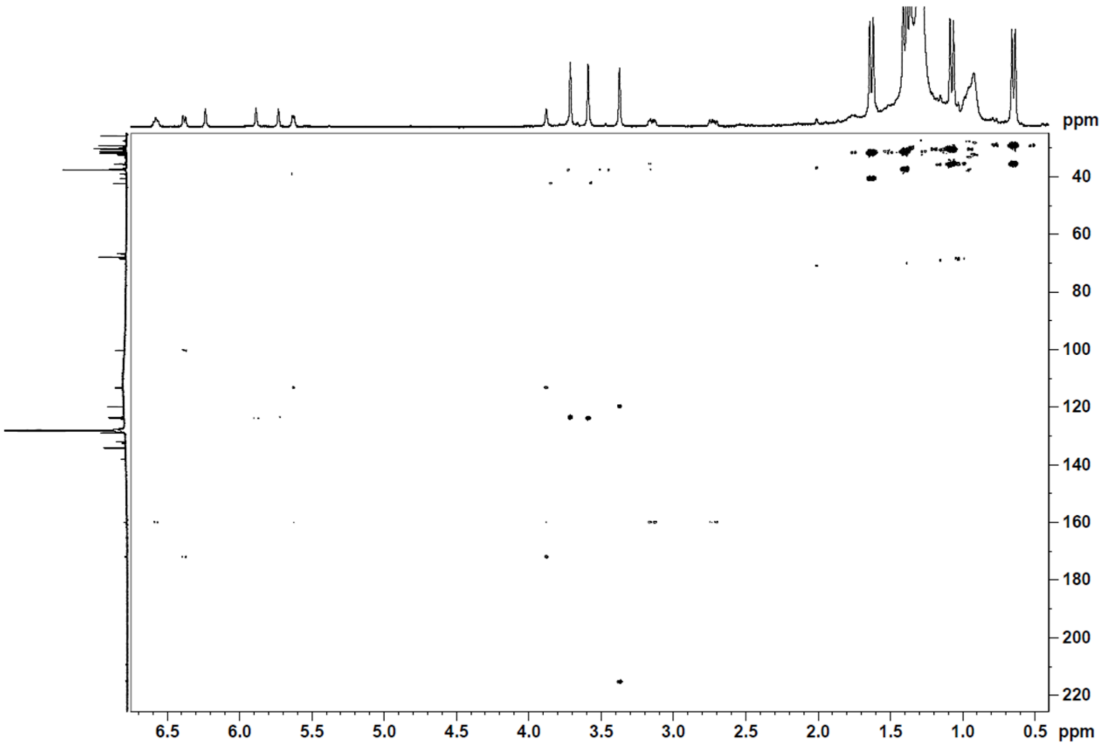

Figure S64: ^{1}H-^{13}C HMBC NMR spectrum of [Tc(^{Pyr}PNP^{tBu})(CO)$_2$(DMIM)] (**10**).

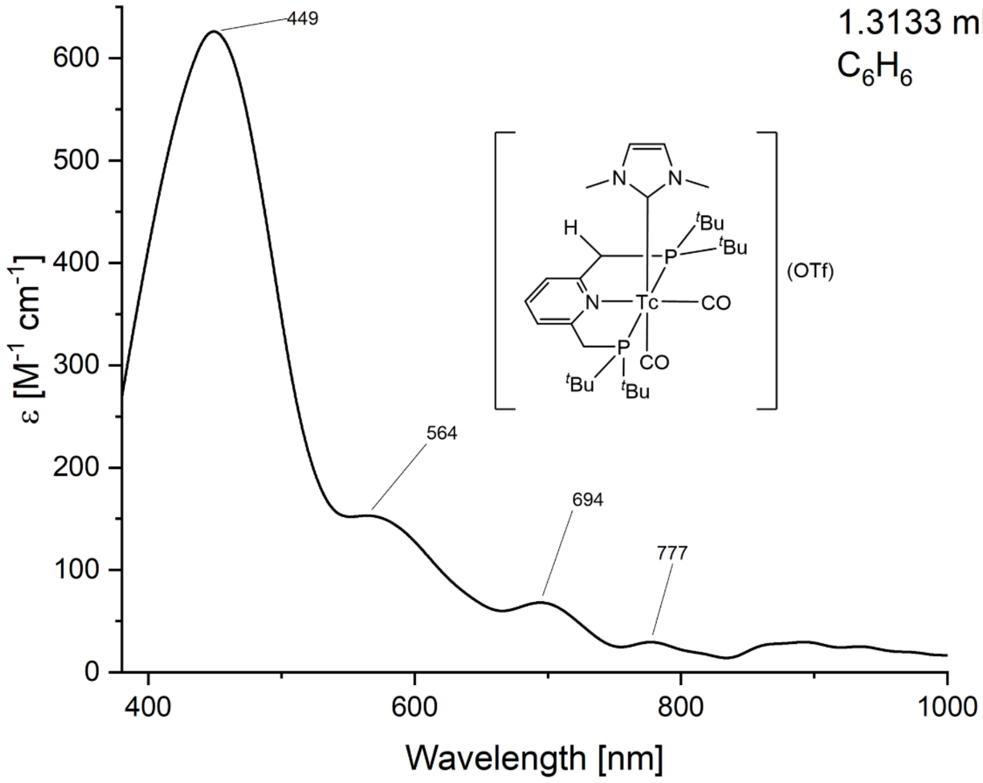

Figure S65: VIS spectrum (C_6H_6) of [Tc(^{Pyr}PNP^{tBu})(CO)$_2$(DMIM)] (**10**).

4.9 [Tc(^{Pyr}PNP^{tBu})(CO)$_2$(NC$_4$H$_4$)] (11)

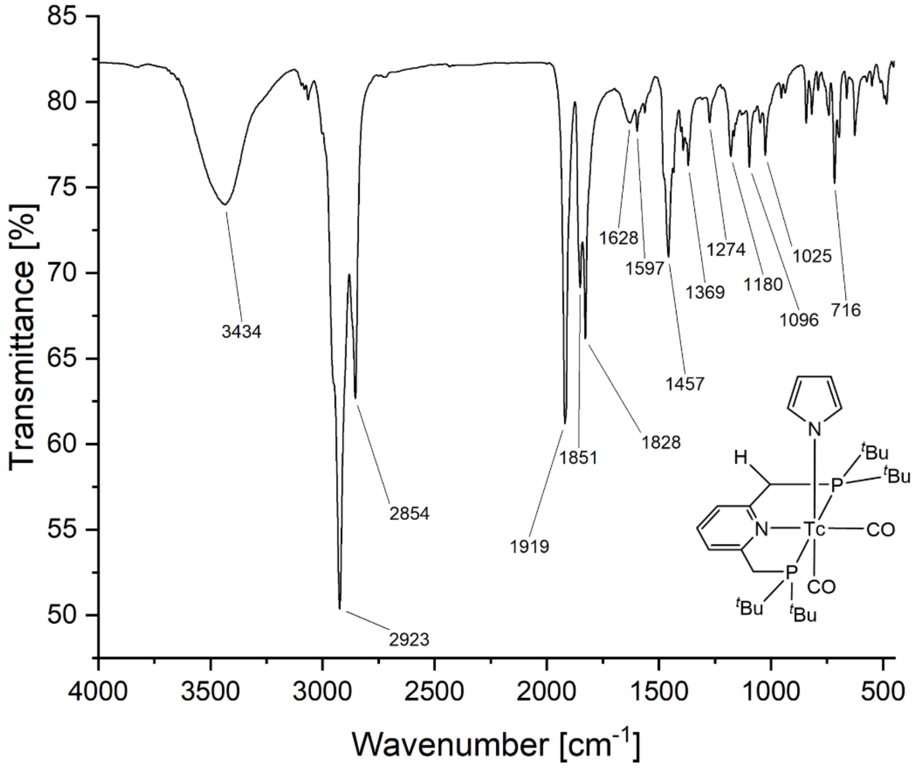

Figure S66: IR spectrum (KBr) of [Tc(^{Pyr}PNP^{tBu})(CO)$_2$(NC$_4$H$_4$)] (**11**).

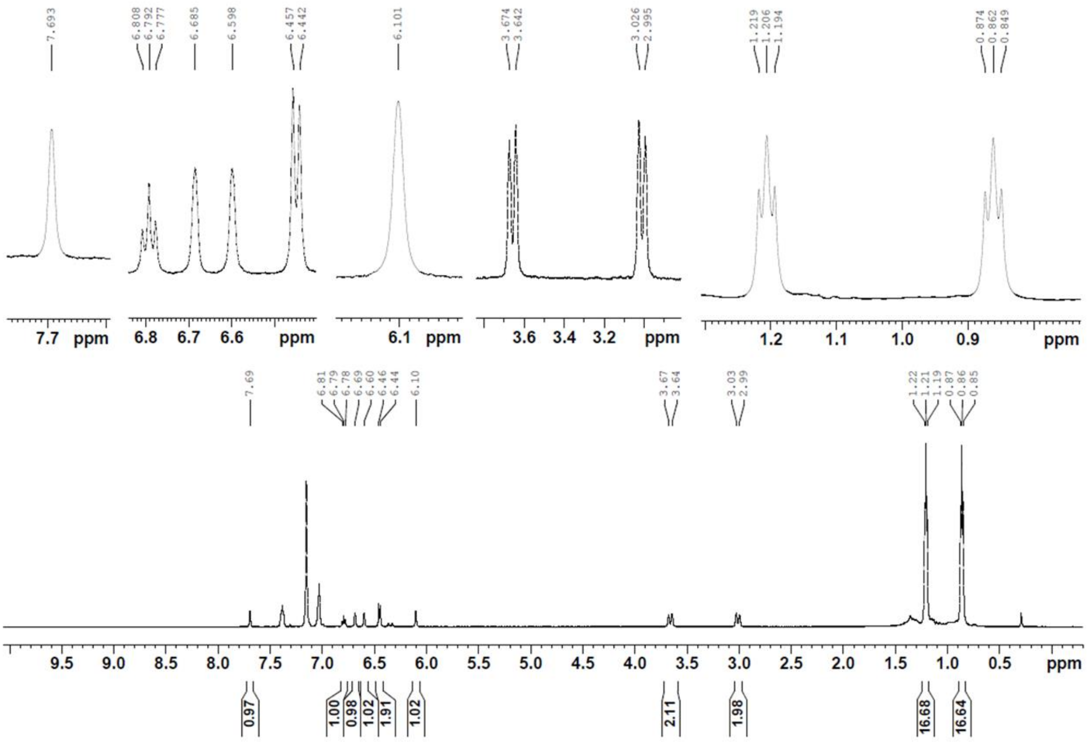

Figure S67: ^{1}H NMR spectrum of [Tc(^{Pyr}PNP^{tBu})(CO)$_2$(NC$_4$H$_4$)] (**11**).

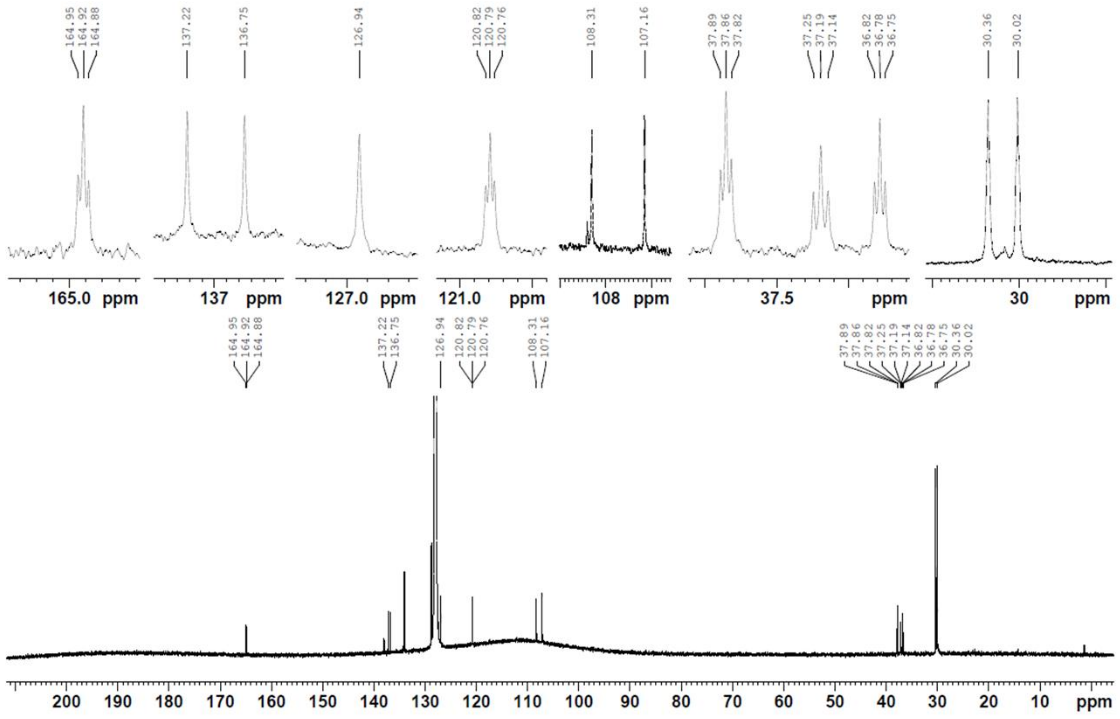

Figure S68: $^{13}C\{^{1}H\}$ NMR spectrum of [Tc(^{Pyr}PNP^{tBu})(CO)$_2$(NC$_4$H$_4$)] (**11**).

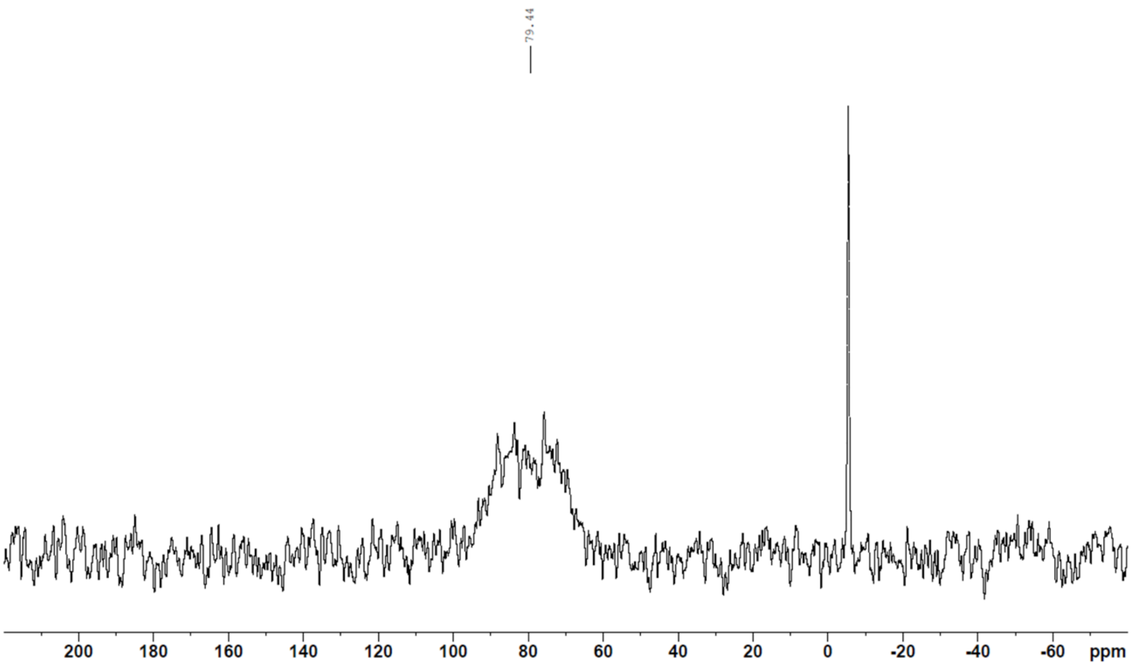

Figure S69: $^{31}P\{^{1}H\}$ NMR spectrum of [Tc(^{Pyr}PNP^{tBu})(CO)$_2$(NC$_4$H$_4$)] (**11**).

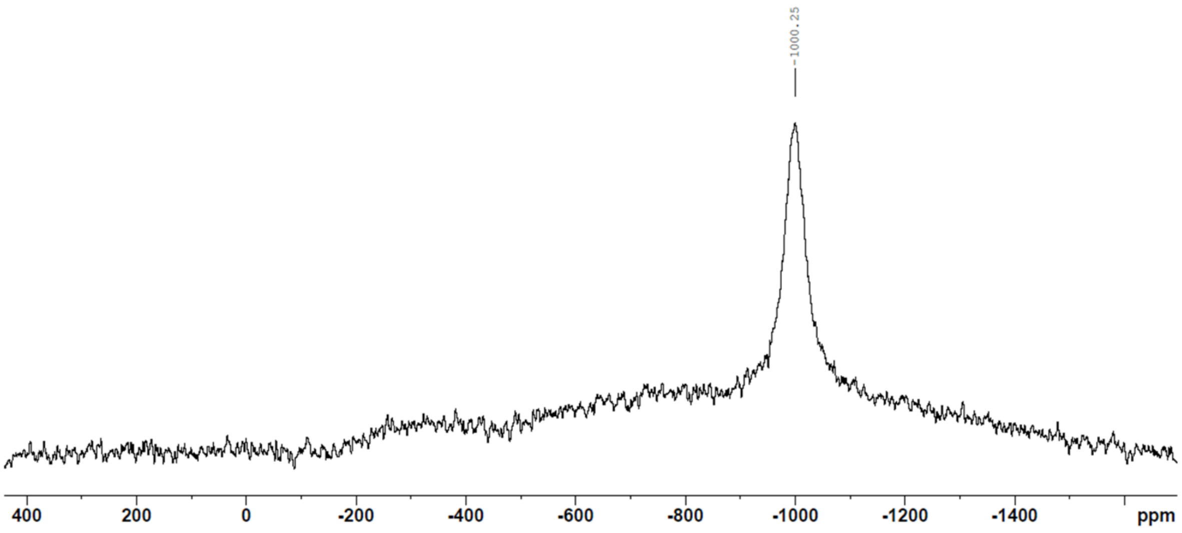

Figure S70: ^{99}Tc NMR spectrum of [Tc(^{Pyr}PNP^{tBu})(CO)$_2$(NC$_4$H$_4$)] (**11**).

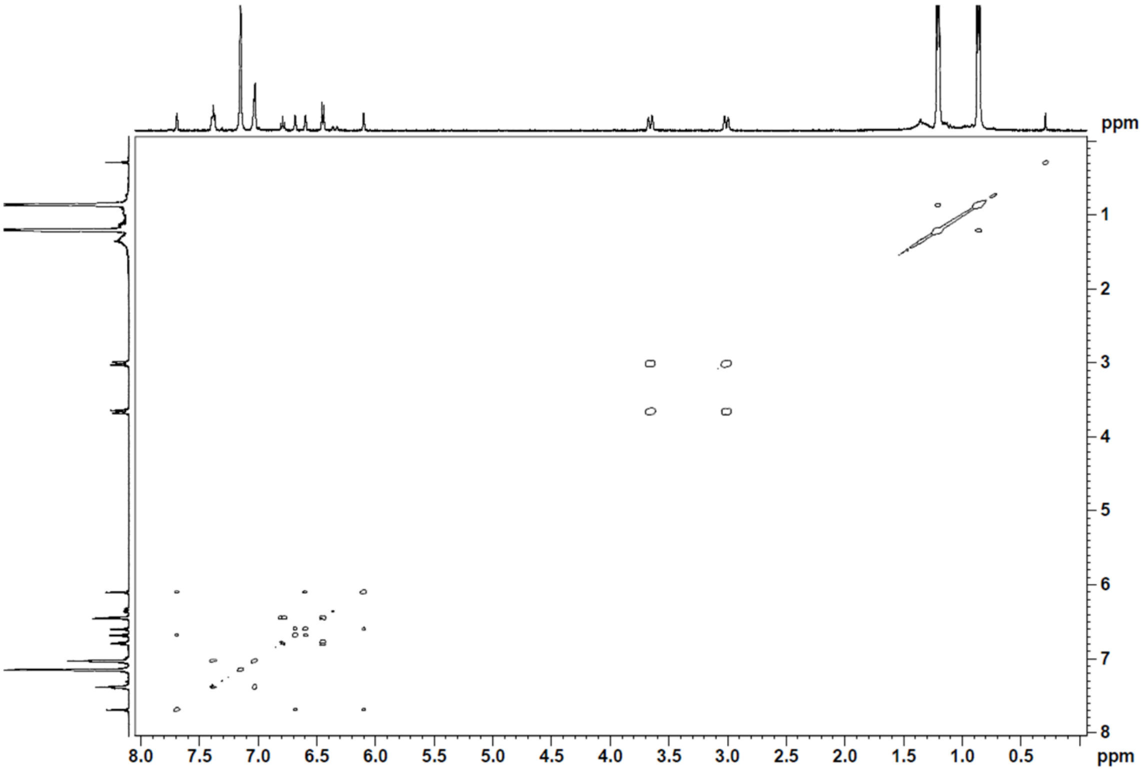

Figure S71: ^{1}H-^{1}H COSY NMR spectrum of [Tc(^{Pyr}PNP^{tBu})(CO)$_2$(NC$_4$H$_4$)] (**11**).

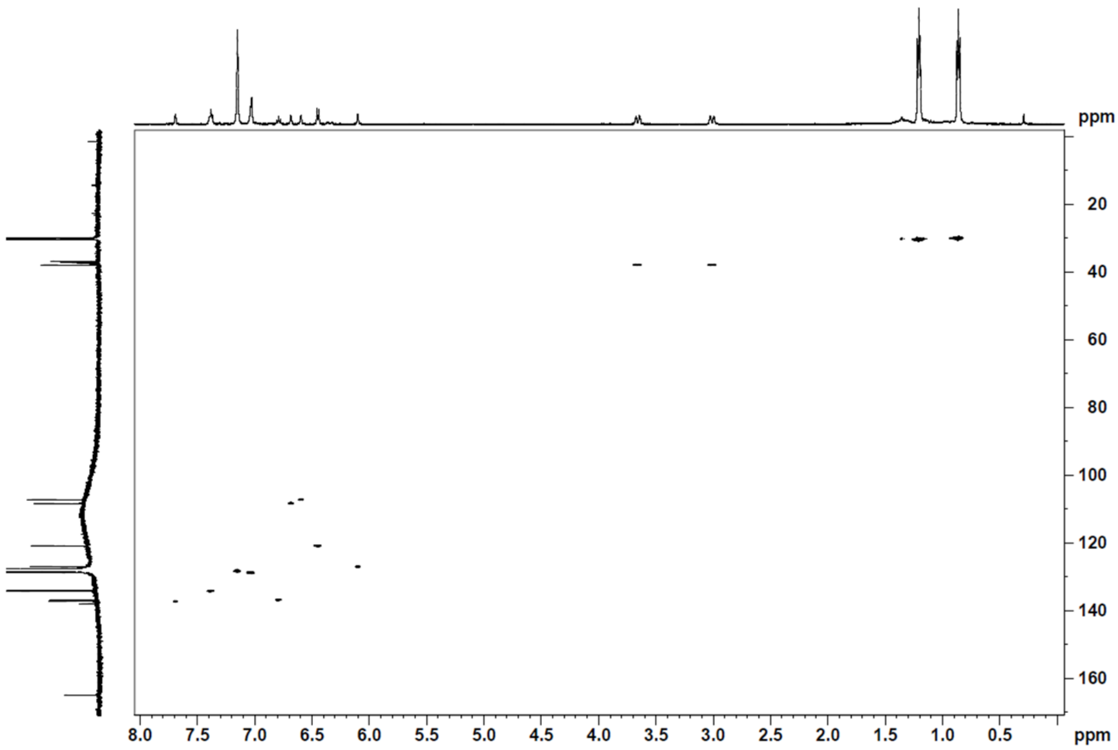

Figure S72: ^{1}H-^{13}C HSQC NMR spectrum of [Tc(^{Pyr}PNP^{tBu})(CO)$_2$(NC$_4$H$_4$)] (**11**).

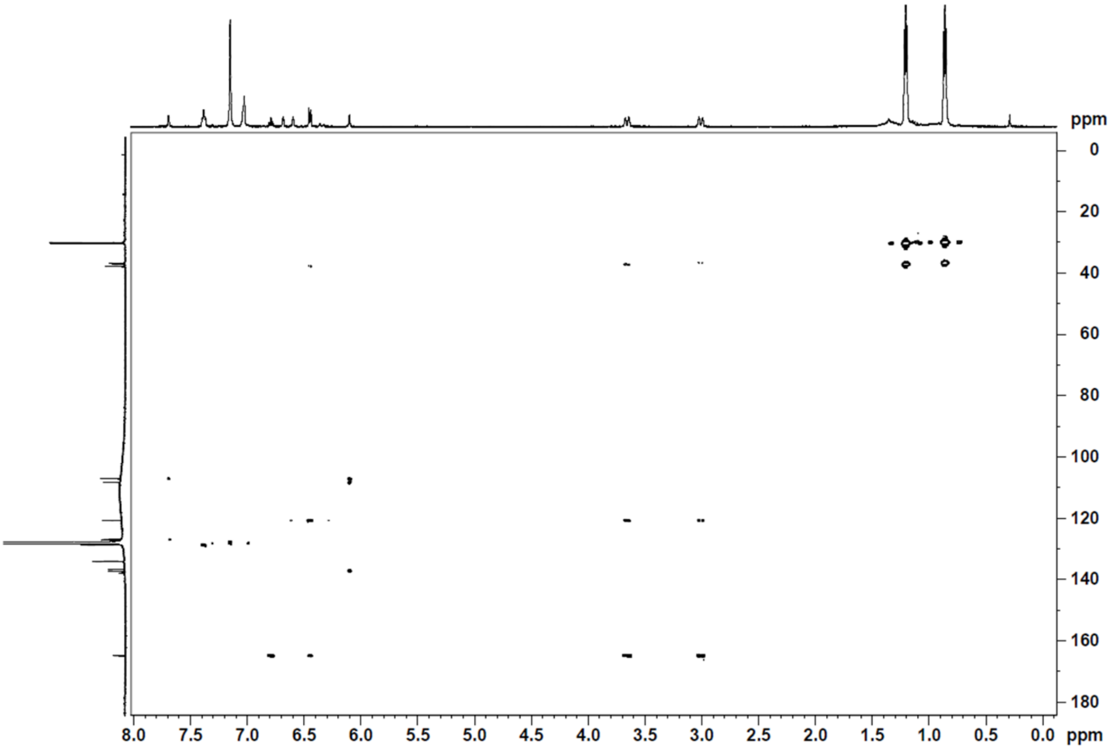

Figure S73: ^{1}H-^{13}C HMBC NMR spectrum of [Tc(^{Pyr}PNP^{tBu})(CO)$_2$(NC$_4$H$_4$)] (**11**).

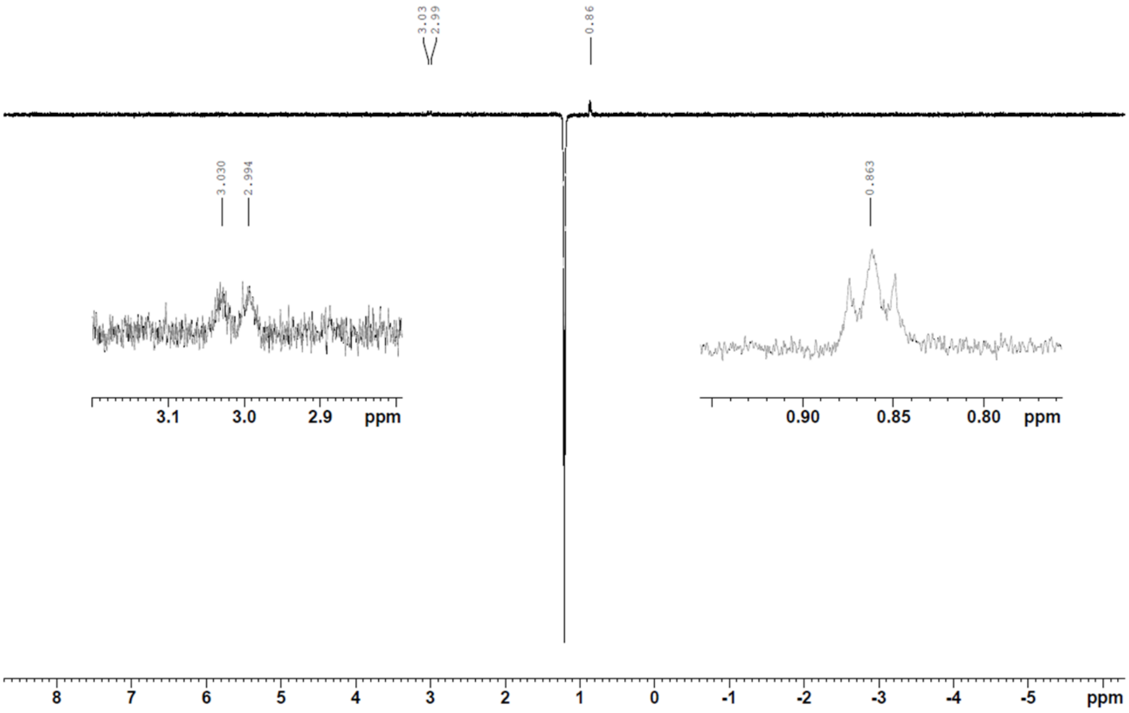

Figure S74: 1D-NOE ^{1}H NMR spectrum of [Tc(^{Pyr}PNP^{tBu})(CO)$_2$(NC$_4$H$_4$)] (**11**), irradiated on 1.21 ppm (^{t}Bu signal).

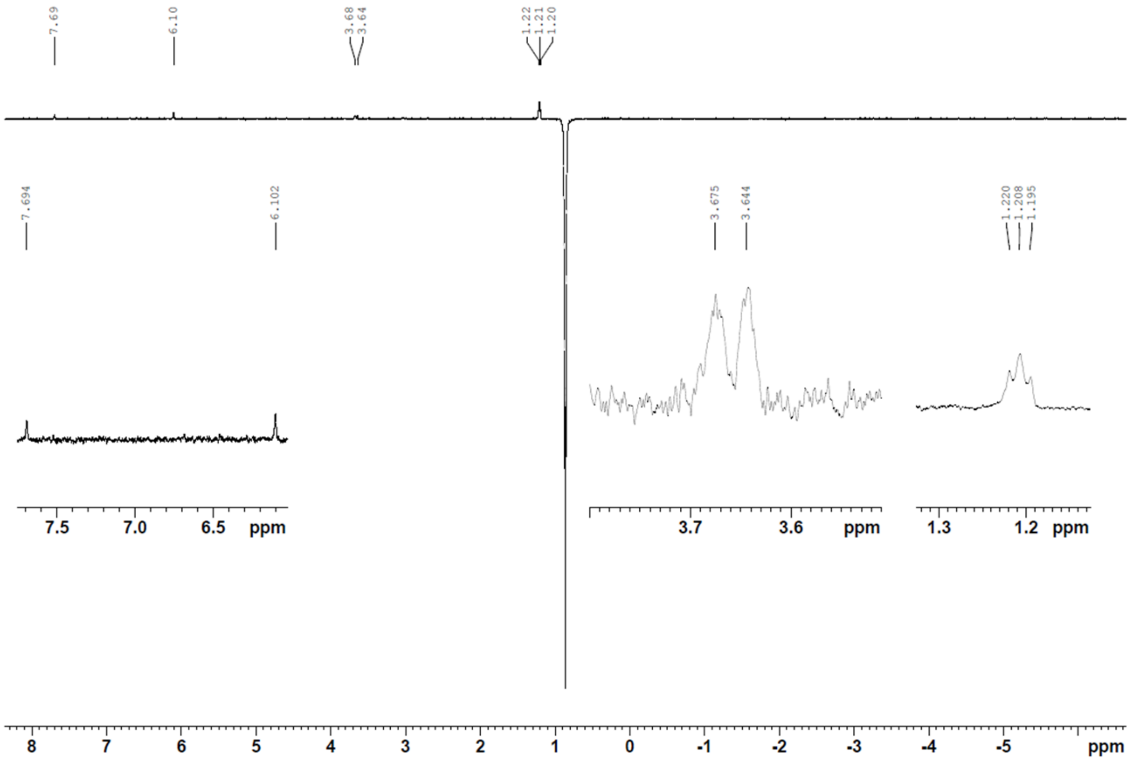

Figure S75: 1D-NOE ^{1}H NMR spectrum of [Tc(^{Pyr}PNP^{tBu})(CO)$_2$(NC$_4$H$_4$)] (**11**), irradiated on 0.86 ppm (^{t}Bu signal).

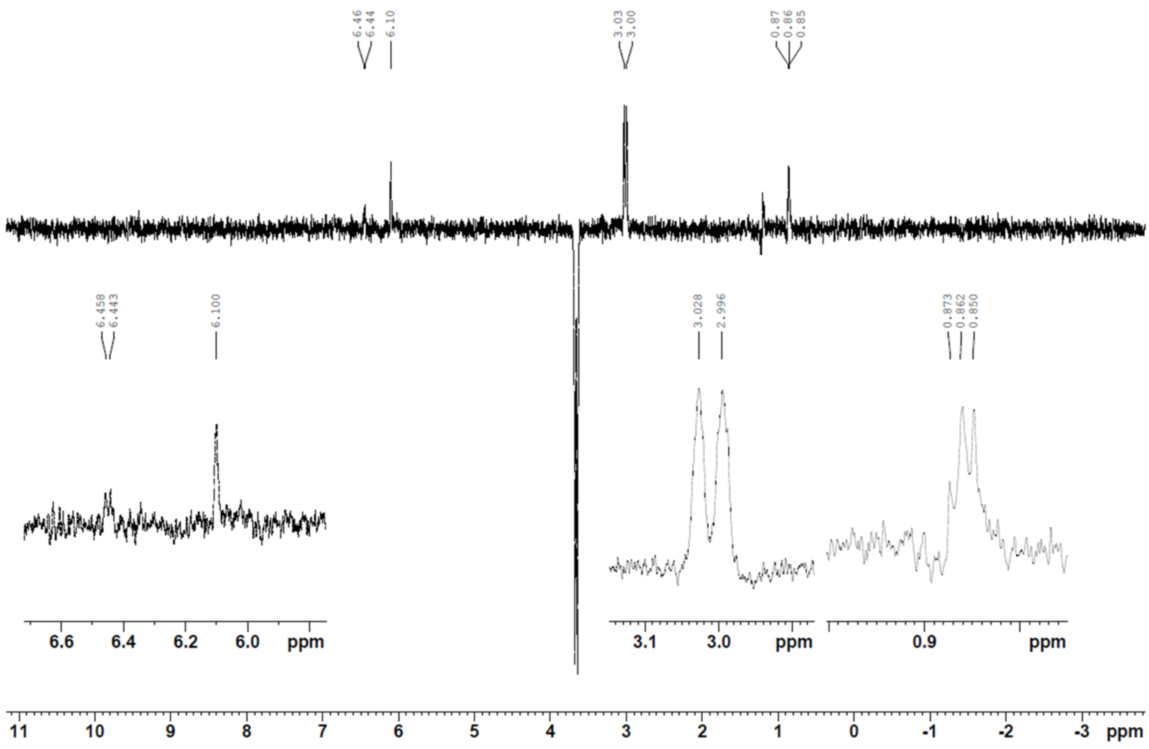

Figure S76: 1D-NOE ^{1}H NMR spectrum of [Tc(^{Pyr}PNP^{tBu})$(CO)_2(NC_4H_4)$] (**11**), irradiated on 3.66 ppm (PCH_2 signal).

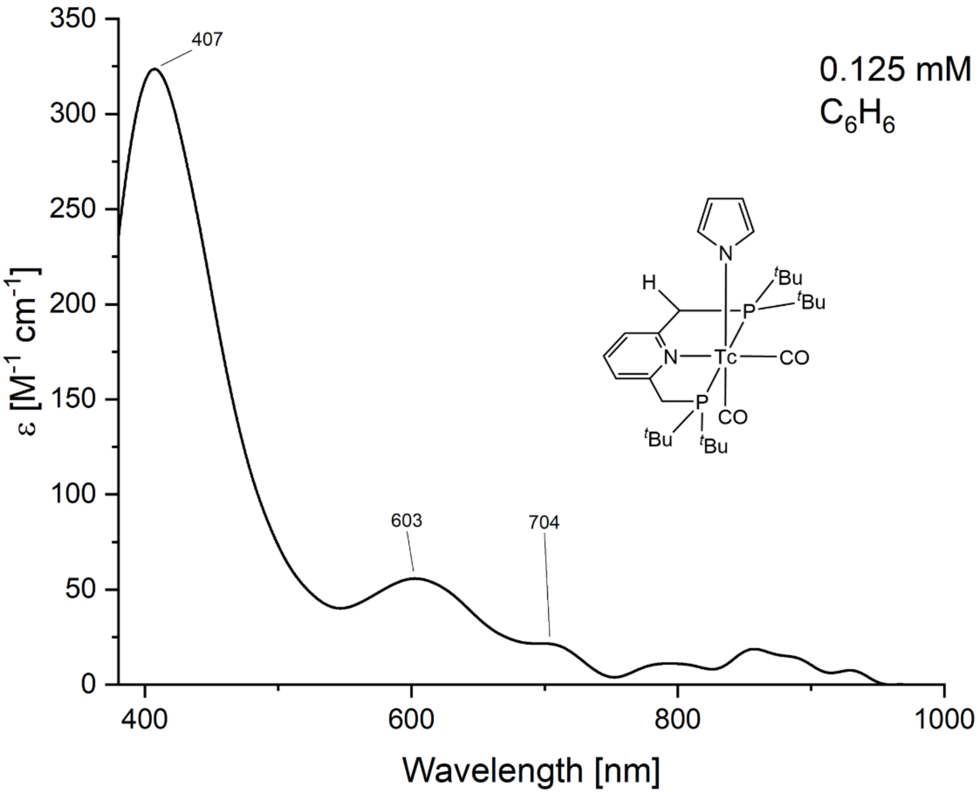

Figure S77: VIS spectrum (C_6H_6) of [Tc(^{Pyr}PNP^{tBu})$(CO)_2(NC_4H_4)$] (**11**).

4.10 [Tc(^{Pyr}PNP^{tBu})(CO)$_2$(N$_2$C$_3$H$_3$)] (12)

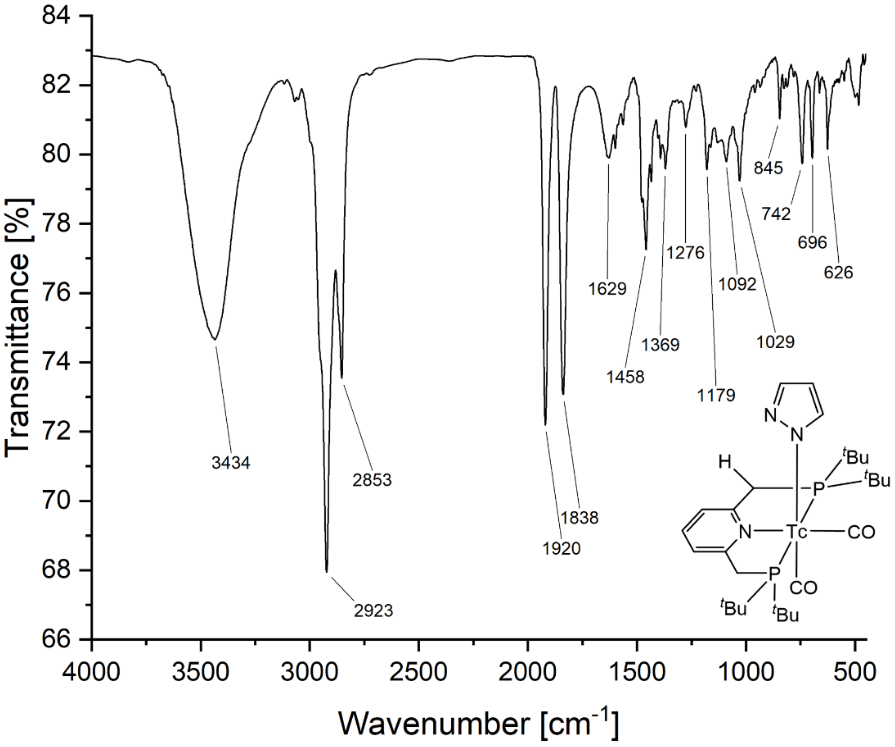

Figure S78: IR spectrum (KBr) of [Tc(^{Pyr}PNP^{tBu})(CO)$_2$(N$_2$C$_3$H$_3$)] (**12**).

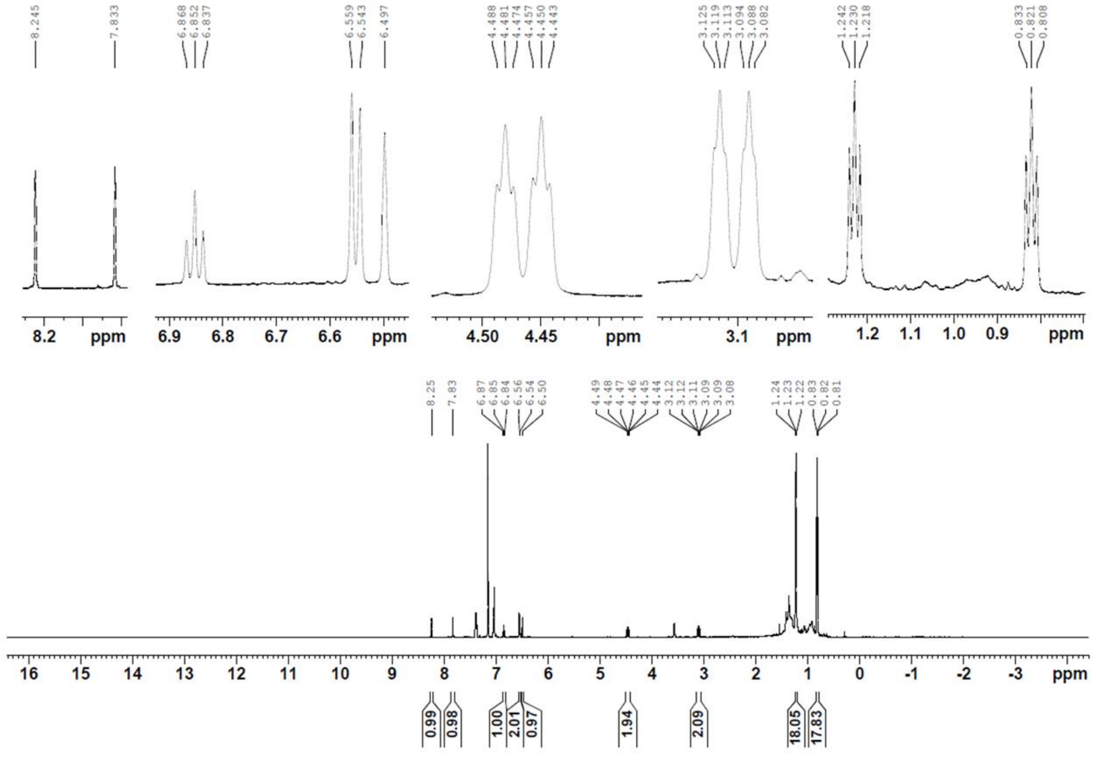

Figure S79: ^{1}H NMR spectrum of [Tc(^{Pyr}PNP^{tBu})(CO)$_2$(N$_2$C$_3$H$_3$)] (**12**).

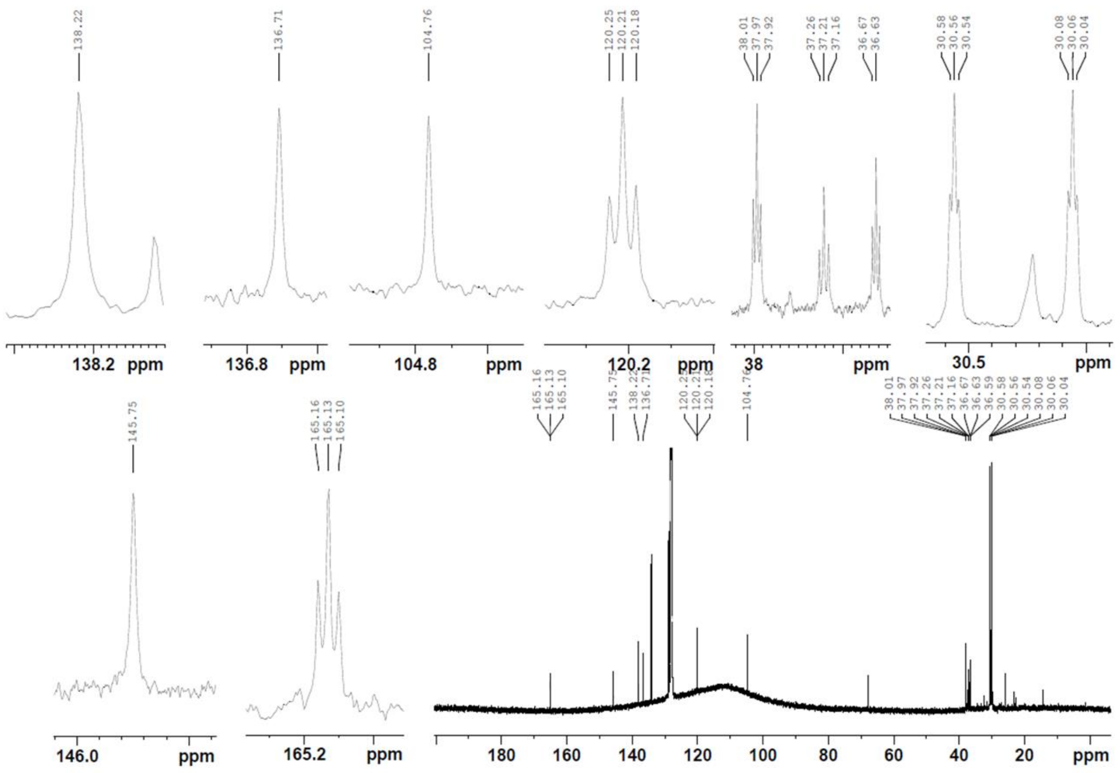

Figure S80: $^{13}C\{^1H\}$ NMR spectrum of $[Tc(^{Pyr}PNP^{tBu})(CO)_2(N_2C_3H_3)]$ (**12**).

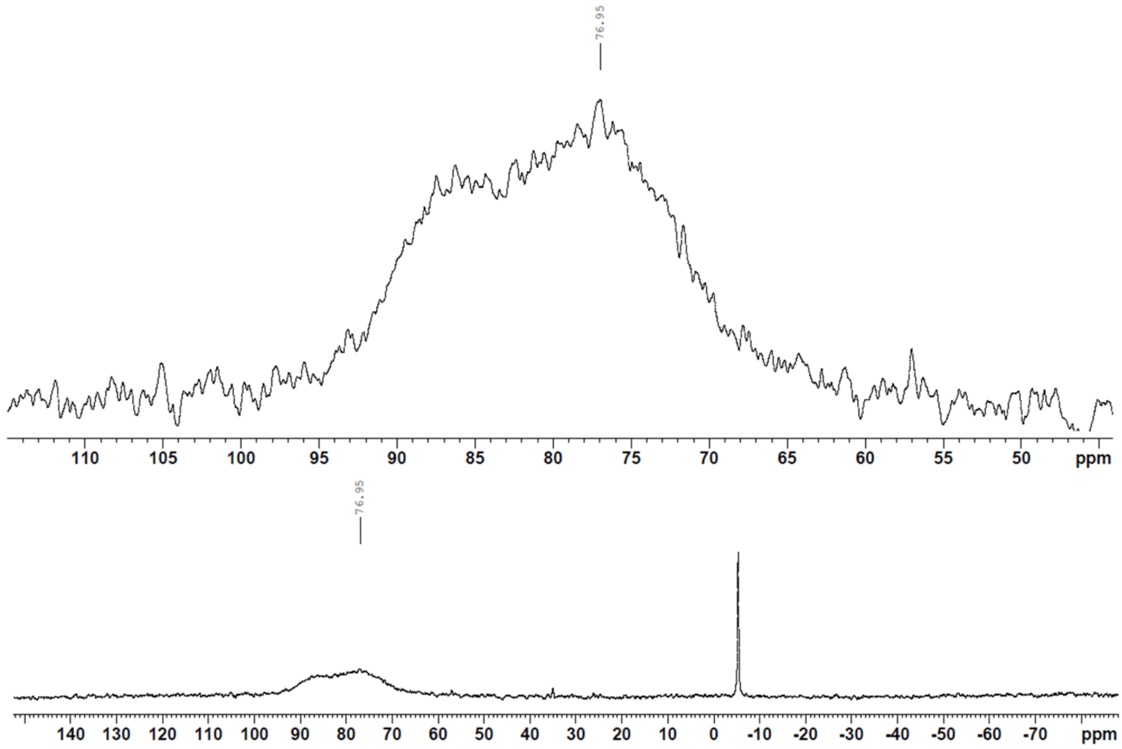

Figure S81: $^{31}P\{^1H\}$ NMR spectrum of $[Tc(^{Pyr}PNP^{tBu})(CO)_2(N_2C_3H_3)]$ (**12**).

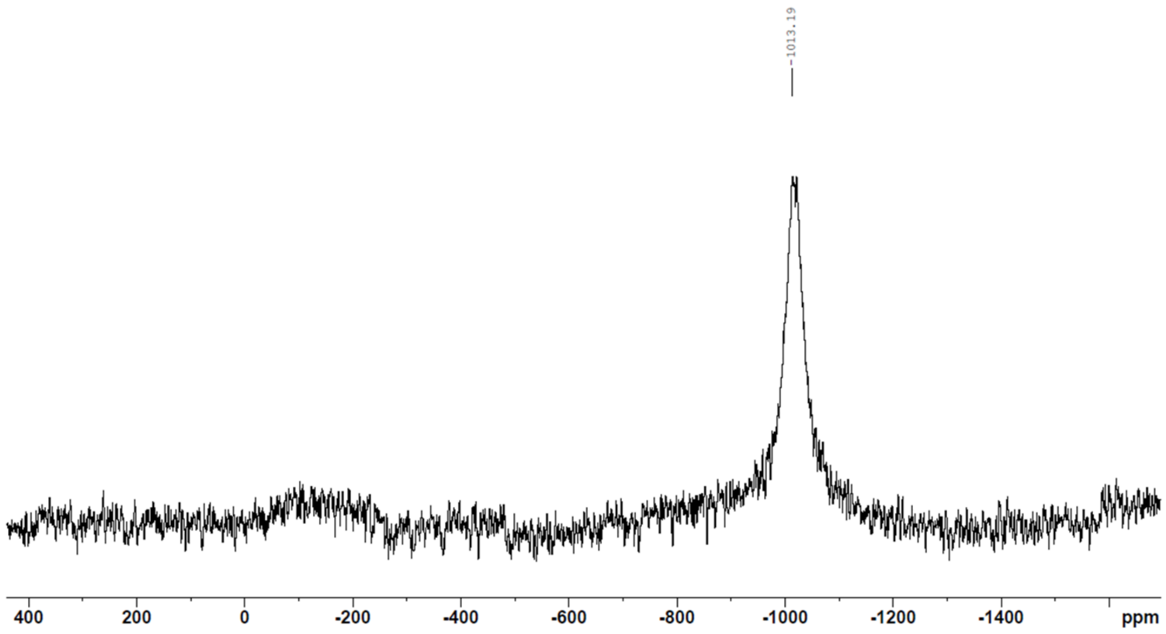

Figure S82: ^{99}Tc NMR spectrum of [Tc(^{Pyr}PNP^{tBu})(CO)$_2$(N$_2$C$_3$H$_3$)] (**12**).

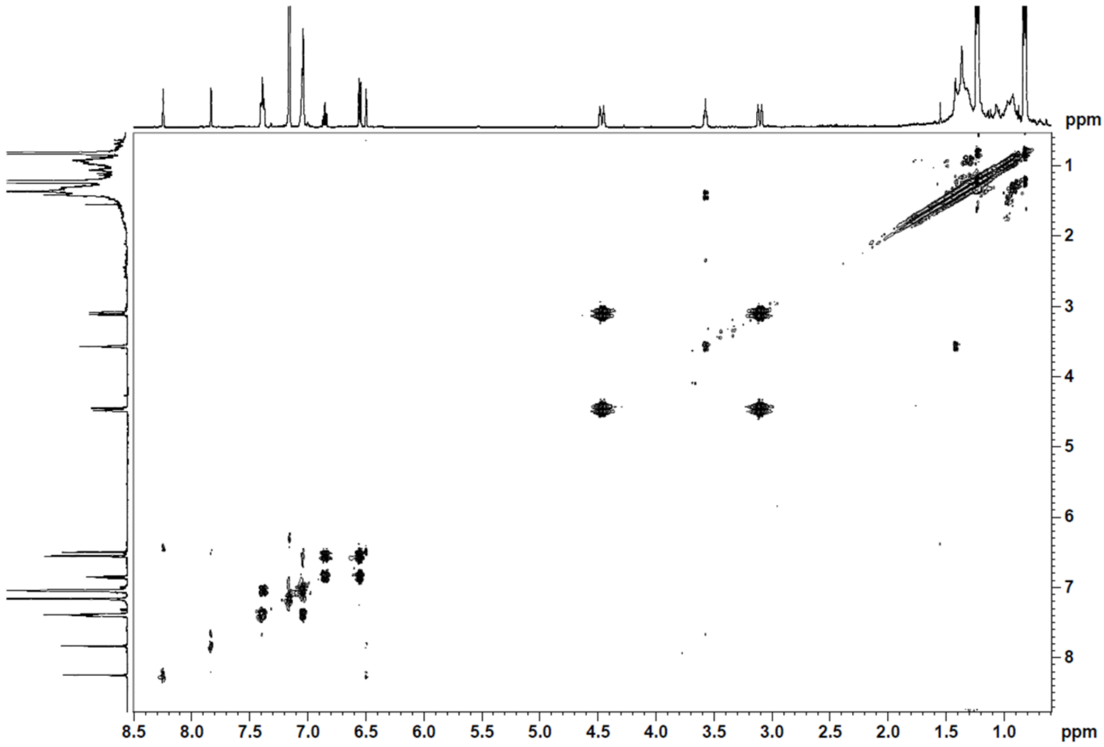

Figure S83: ^{1}H-^{1}H COSY NMR spectrum of [Tc(^{Pyr}PNP^{tBu})(CO)$_2$(N$_2$C$_3$H$_3$)] (**12**).

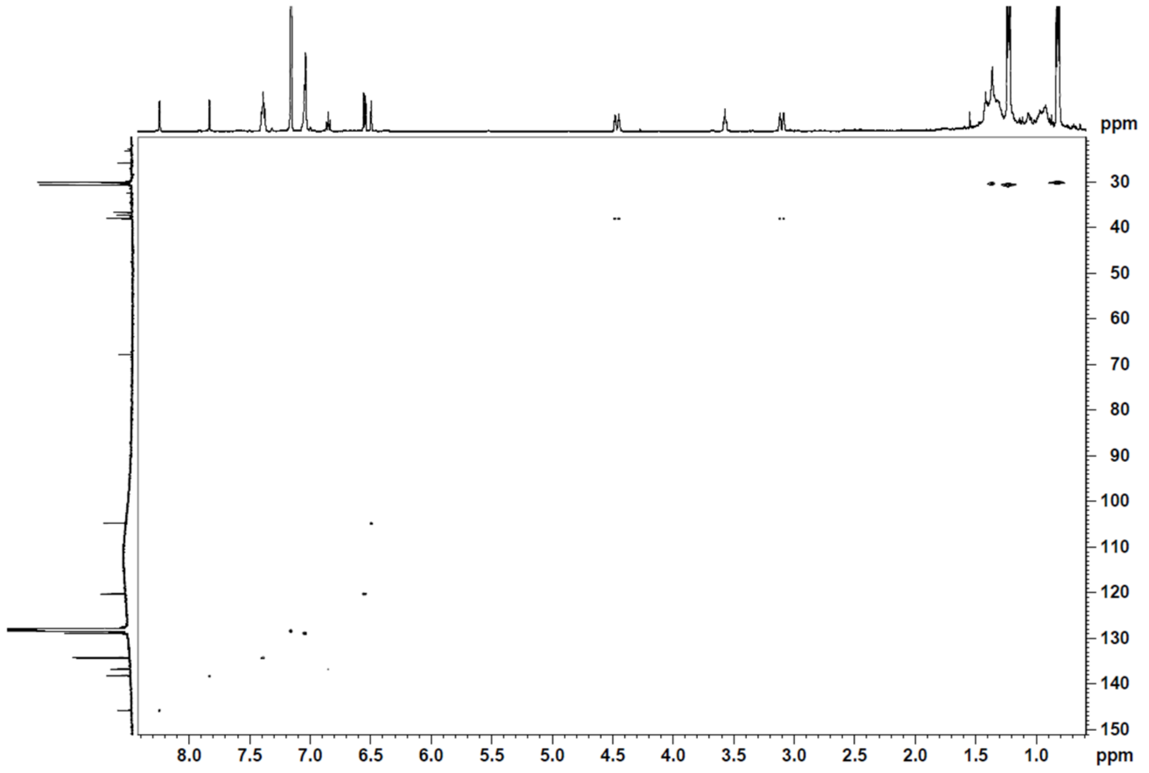

Figure S84: ^{1}H-^{13}C HSQC NMR spectrum of [Tc(^{Pyr}PNP^{tBu})(CO)$_2$(N$_2$C$_3$H$_3$)] (**12**).

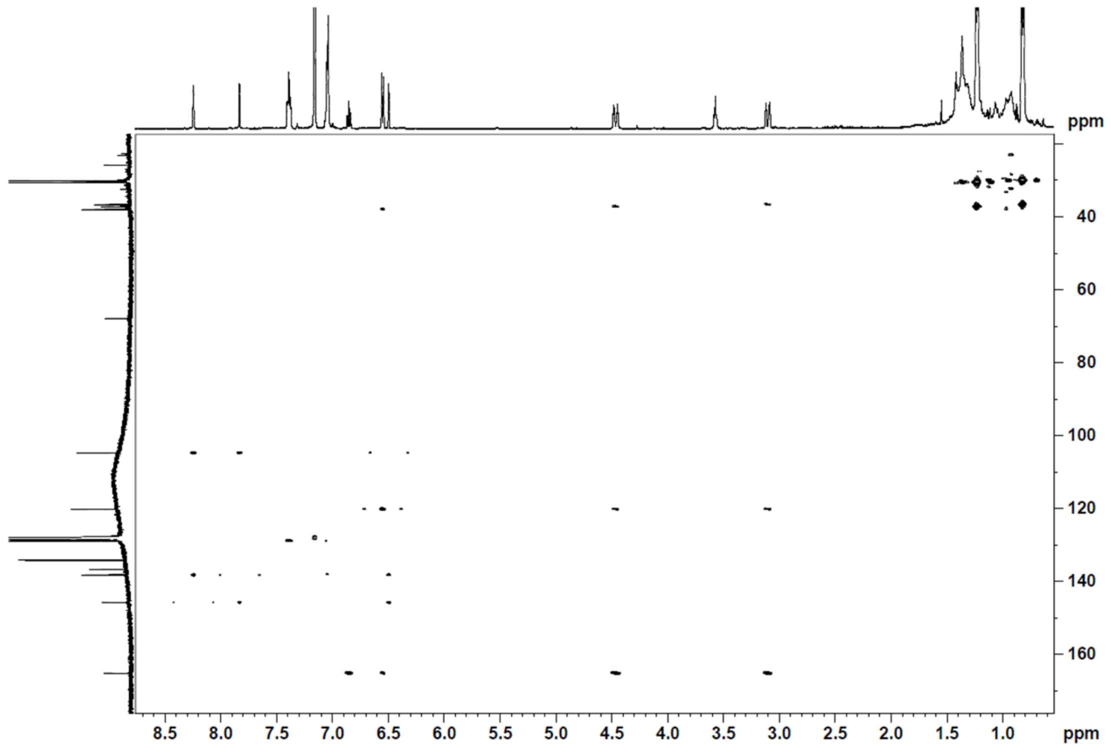

Figure S85: ^{1}H-^{13}C HMBC NMR spectrum of [Tc(^{Pyr}PNP^{tBu})(CO)$_2$(N$_2$C$_3$H$_3$)] (**12**).

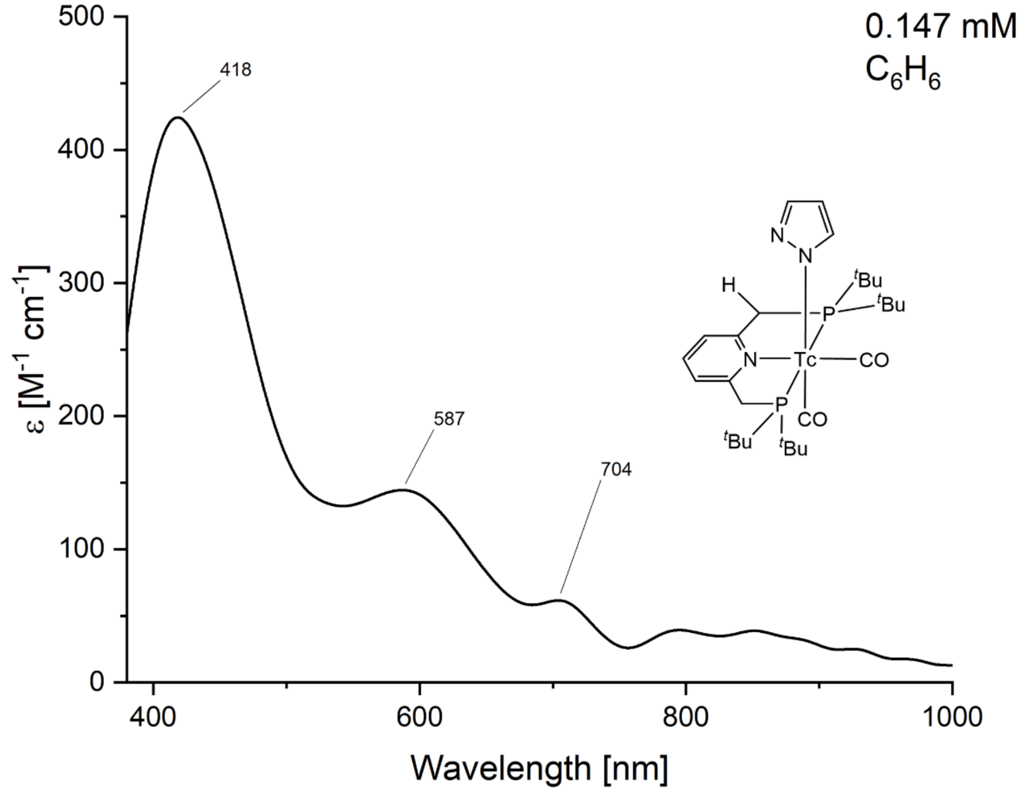

Figure S86: VIS spectrum (C_6H_6) of [Tc(^{Pyr}PNP^{tBu})(CO)$_2$($N_2C_3H_3$)] (**12**).

5 Crystallographic Data

CCDC entries 2254471-2254476 contain the supplementary crystallographic data for this paper. These data are provided free of charge by The Cambridge Crystallographic Data Centre *via* www.ccdc.cam.ac.uk/structures.

5.1 [Tc(^{Pyr}PNP^{tBu})Cl(CO)$_2$] (3)

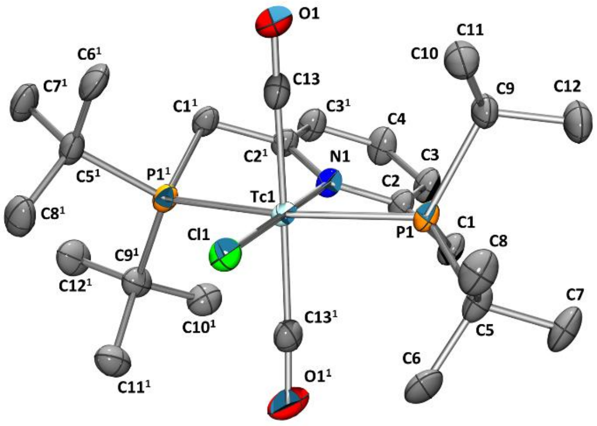

Figure S87: Ellipsoid displacement plot[11] of *trans*-[Tc(^{Pyr}PNP^{tBu})Cl(CO)$_2$] (**3**). Ellipsoids represent 35% probability. Hydrogen atoms are omitted for clarity.

Table S1: Tabulated values of selected bond lengths and angles in the crystal structure of [**3**].

	Selected bond lengths		Selected bond angles
Tc1–Cl1	2.5261(10) Å	P1–Tc1–P1^{1}	159.43(4)°
Tc1–P1	2.4092(7) Å	C13–Tc1–N1	91.62(9)°
Tc1–N1	2.136(3) Å	C13–Tc1–C13^{1}	176.76(19)°
C1–C2	1.498(4) Å	N1–Tc1–Cl1	180.0°
C13–O1	1.109(4)) Å	P1–Tc1–N1	79.713(18)°
C1–C2	1.498(4) Å	C13–Tc1–Cl1	88.38(10)°
P1–Tc1–Cl1	100.287(18)°	C13–Tc1–P1	91.25(9)°

Table S2: Crystallographic data of [Tc(^{Pyr}PNP^{tBu})Cl(CO)$_2$] (**3**)

Empirical formula	$C_{25}H_{43}ClNO_2P_2Tc$
Formula weight	584.99
Diffractometer	Rigaku OD XtaLAB Synergy, Dualflex, Pilatus 200K
Radiation	CuKα (λ = 1.54184 Å)
Temperature [K]	160
Crystal system	hexagonal
Space group	$P6_122$
a [Å]	16.07210 (11)
b [Å]	16.07210 (11)
c [Å]	20.66580 (13)
α [°]	90
β [°]	90
γ [°]	120
Volume [Å3]	4623.04 (7)
Z	6
ρ_{calc} [g/cm^3]	1.261
μ [mm^{-1}]	5.72
F(000)	1836
Crystal size [mm^3]	0.13 × 0.06 × 0.04
Crystal description	red plate
2Θ range for data collection [°]	3.2 to 75.5
Index ranges	$-15 \leq h \leq 17$, $-14 \leq k \leq 10$, $-21 \leq l \leq 25$
Reflections collected	11130
Independent reflections	3167 [R_{int} = 0.0232, R_{sigma} = 0.0229]
Reflections observed	3076
Criterion for observation	I>2σ (I)
Completeness to theta	99.9% to 75.635°
Absorption correction	gaussian
Min./max. transmission	0.678/0.882
Data/restraints/parameters	3167/0/153
Goodness-of-fit on F^2	1.07
Final R indexes [I>=2σ (I)]	R_1 = 0.0229, wR_2 = 0.0609
Final R indexes [all data]	R_1 = 0.0237, wR_2 = 0.0613
Largest diff. peak/hole / [e Å^{-3}]	0.41/−0.31

5.2 [Tc(^{Pyr}PNP^{tBu*})(CO)$_2$] (4)

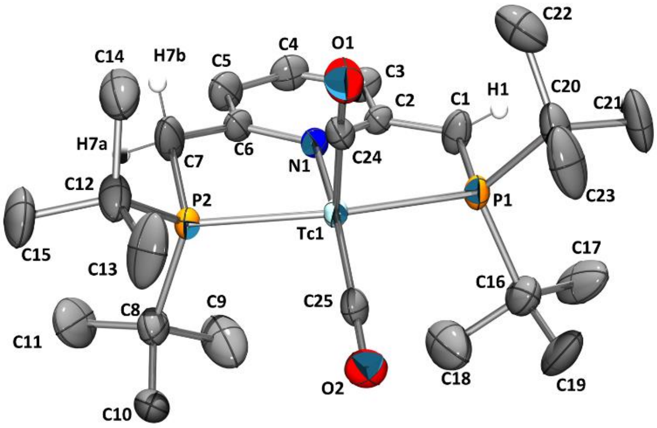

Figure S88: Ellipsoid displacement plot[11] of [Tc(^{Pyr}PNP^{tBu*})(CO)$_2$] (**4**). Ellipsoids represent 35% probability. Hydrogen atoms, except H1, H7a and H7b, and the minor component of the disordered Tc(CO) unit are omitted for clarity.

Table S3: Tabulated values of selected bond lengths and angles in the crystal structure of [**4**].

	Selected bond lengths		Selected bond angles
Tc1–P1	2.4269(12) Å	P1–Tc1–P2	160.36(5)°
Tc1–P2	2.3867(11) Å	P1–Tc1–C24	96.48(15)°
Tc1–N1	2.163(3) Å	P2–Tc1–C24	95.99(16)°
Tc1–C24	1.836(5) Å	P1–Tc1–C25	98.30(14)°
Tc1–C25	1.886(5) Å	P2–Tc1–C25	97.40(13)°
C1–C2	1.390(6) Å	C24–Tc1–C25	87.2(2)°
C6–C7	1.482(8) Å	C24–Tc1–N1	109.58(19)°
C24–O1	1.154(6) Å	C25–Tc1–N1	163.22(17)°
C25–O2	1.164(5) Å	P1–C1–C2–N1	9.0(6)°
P1–Tc1–N1	80.48(9)°	P2–C7–C6–N1	-29.0(7)°
P2–Tc1–N1	81.08(9)°		

Table S4: Crystallographic data of [Tc(^{Pyr}PNP^{tBu}*)(CO)$_2$] (**4**)

Empirical formula	$C_{25}H_{42}NO_2P_2Tc$
Formula weight	548.53
Diffractometer	Rigaku OD XtaLAB Synergy, Dualflex, Pilatus 200K
Radiation	CuKα (λ = 1.54184 Å)
Temperature [K]	160
Crystal system	orthorhombic
Space group	Pccn
a [Å]	30.8956 (12)
b [Å]	15.6078 (7)
c [Å]	11.2491 (5)
α [°]	90
β [°]	90
γ [°]	90
Volume [Å3]	5424.4 (4)
Z	8
ρ_{calc} [g/cm^3]	1.343
μ [mm^{-1}]	5.58
F(000)	2304
Crystal size [mm^3]	0.06 × 0.05 × 0.03
Crystal description	brown prism
2Θ range for data collection [°]	2.9 to 78.8
Index ranges	$-38 \leq h \leq 22$, $-19 \leq k \leq 19$, $-14 \leq l \leq 14$
Reflections collected	20999
Independent reflections	5434 [R_{int} = 0.057, R_{sigma} = 0.0494]
Reflections observed	4056
Criterion for observation	I>2σ (I)
Completeness to theta	98.9% to 74.503°
Absorption correction	analytical
Min./max. transmission	0.798/0.884
Data/restraints/parameters	5434/27/320
Goodness-of-fit on F^2	1.08
Final R indexes [I>=2σ (I)]	R_1 = 0.0494, wR_2 = 0.1305
Final R indexes [all data]	R_1 = 0.0691, wR_2 = 0.1394
Largest diff. peak/hole / [e Å^{-3}]	0.67/−0.56

5.3 [Tc(^{Pyr}PNP^{tBu}–CSS)(CO)$_2$] (6)

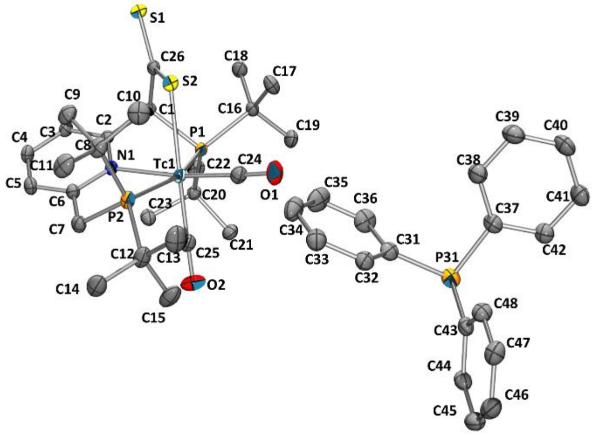

Figure S89: Ellipsoid displacement plot[11] of [Tc(^{Pyr}PNP^{tBu}–CSS)(CO)$_2$] (**6**). Ellipsoids represent 35% probability. Hydrogen atoms are omitted for clarity.

Table S5: Tabulated values of selected bond lengths and angles in the crystal structure of [**6**].

	Selected bond lengths		Selected bond angles
Tc1–P1	2.4296(4) Å	P1–Tc1–C24	101.80(5)°
Tc1–P2	2.4303(4) Å	P2–Tc1–C24	99.53(5)°
Tc1–N1	2.1743(12) Å	P1–Tc1–C25	91.35(5)°
Tc1–C24	1.9000(16) Å	P2–Tc1–C25	90.70(5)°
Tc1–C25	1.8918(17) Å	C24–Tc1–C25	91.39(7)°
C1–C2	1.5135(19) Å	C24–Tc1–N1	172.69(6)°
C6–C7	1.506(2) Å	C25–Tc1–N1	95.64(6)°
C24–O1	1.158(2) Å	P1–Tc1–S2	79.714(12)°
C25–O2	1.161(2) Å	P2–Tc1–S2	97.042(13)°
P1–Tc1–N1	80.09(3)°	S2–Tc1–C25	170.92(5)°
P2–Tc1–N1	78.42(3)°	S1–C26–S2	124.31(9)°
P1–Tc1–P2	158.513(13)°	S1–C26–C1	119.31(10)°

Table S6: Crystallographic data of [Tc(^{Pyr}PNP^{tBu}–CSS)(CO)$_2$] (**6**)

Empirical formula	$C_{26}H_{42}NO_2P_2S_2Tc\cdot C_{18}H_{15}P$
Formula weight	886.93
Diffractometer	Rigaku OD XtaLAB Synergy, Dualflex, Pilatus 200K
Radiation	CuKα (λ = 1.54184 Å)
Temperature [K]	160
Crystal system	triclinic
Space group	$P\bar{1}$
a [Å]	8.8180 (1)
b [Å]	15.5719 (1)
c [Å]	17.0481 (1)
α [°]	71.836 (1)
β [°]	83.648 (1)
γ [°]	82.143 (1)
Volume [Å3]	2197.63 (35)
Z	2
ρ_{calc} [g/cm^3]	1.340
μ [mm^{-1}]	4.86
F(000)	928
Crystal size [mm^3]	0.12 × 0.06 × 0.02
Crystal description	needle, orange
2Θ range for data collection [°]	2.7 to 73.8
Index ranges	$-10 \leq h \leq 10$, $-18 \leq k \leq 19$, $-18 \leq l \leq 21$
Reflections collected	60975
Independent reflections	8577 [R_{int} = 0.032, R_{sigma} = 0.021]
Reflections observed	8082
Criterion for observation	I>2σ (I)
Completeness to theta	99.9% to 73.857°
Absorption correction	gaussian
Min./max. transmission	0.706/1.000
Data/restraints/parameters	8577/0/490
Goodness-of-fit on F^2	1.07
Final R indexes [I>=2σ (I)]	R_1 = 0.0208, wR_2 = 0.0566
Final R indexes [all data]	R_1 = 0.0222, wR_2 = 0.0572
Largest diff. peak/hole / [e Å^{-3}]	0.33/−0.36

5.4 [Tc(^{Pyr}PNP^{tBu})(CO)$_2$(–C≡C–SiMe$_3$)] (9)

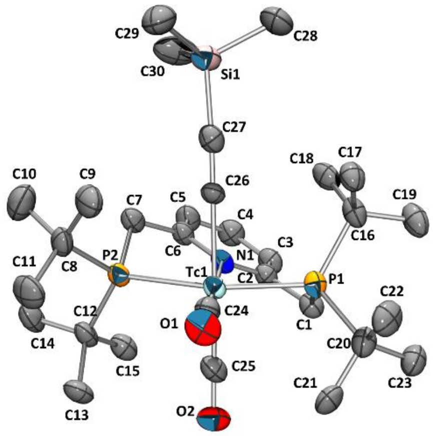

Figure S90: Ellipsoid displacement plot[11] of [Tc(^{Pyr}PNP^{tBu})(CO)$_2$(–C≡C–SiMe$_3$)] (**9**). Ellipsoids represent 35% probability. Hydrogen atoms are omitted for clarity. A second molecule is present in the structure that is not shown for clarity as well.

Table S7: Tabulated values of selected bond lengths and angles in the crystal structure of [**9**].

	Selected bond lengths		Selected bond angles
Tc1–P1	2.4299(15) Å	P1–Tc1–P2	157.64(5)°
Tc1–P2	2.4183(15) Å	P1–Tc1–C24	101.21(19)°
Tc1–N1	2.192(4) Å	P2–Tc1–C24	100.99(19)°
Tc1–C24	1.882(6) Å	P1–Tc1–C25	90.7(2)°
Tc1–C25	1.910(6) Å	P2–Tc1–C25	92.31(19)°
Tc1–C26	2.132(5) Å	C24–Tc1–C25	88.9(2)°
C1–C2	1.516(8) Å	C24–Tc1–N1	179.08(19)°
C6–C7	1.510(8) Å	C25–Tc1–N1	92.0(2)°
C24–O1	1.161(7) Å	P1–Tc1–C26	90.63(15)°
C25–O2	1.173(7) Å	P2–Tc1–C26	85.22(15)°
C26–C27	1.211(7) Å	C26–Tc1–C25	176.4(2)°
P1–Tc1–N1	78.63(12)°	C26–Tc1–N1	84.98(17)°
P2–Tc1–N1	79.12(12)°	Tc1–C26–C27	174.3(5)°

Table S8: Crystallographic data of [Tc(^{Pyr}PNP^{tBu})(CO)$_2$(–C≡C–SiMe$_3$)] (**9**)

Empirical formula	2 $C_{30}H_{52}NO_2P_2SiTc$
Formula weight	1293.51
Diffractometer	Rigaku XtaLAB Synergy, Dualflex, HyPix
Radiation	CuKα (λ = 1.54184 Å)
Temperature [K]	160
Crystal system	triclinic
Space group	$P\bar{1}$
a [Å]	13.5504 (4)
b [Å]	15.1095 (3)
c [Å]	17.5904 (4)
α [°]	89.198(2)
β [°]	83.274 (2)
γ [°]	72.473 (2)
Volume [Å3]	3409.76 (15)
Z	2
ρ_{calc} [g/cm^3]	1.260
μ [mm^{-1}]	4.84
F(000)	1368
Crystal size [mm^3]	0.06 × 0.04 × 0.01
Crystal description	clear yellow plate
2Θ range for data collection [°]	2.6 to 79.4
Index ranges	$-16 \leq h \leq 10$, $-19 \leq k \leq 19$, $-22 \leq l \leq 22$
Reflections collected	52448
Independent reflections	14172 [R_{int} = 0.095, R_{sigma} = 0.0685]
Reflections observed	10030
Criterion for observation	I>2σ (I)
Completeness to theta	98.0% to 82.000°
Absorption correction	gaussian
Min./max. transmission	0.828/0.975
Data/restraints/parameters	14172/0/697
Goodness-of-fit on F^2	1.15
Final R indexes [I>=2σ (I)]	R_1 = 0.0685, wR_2 = 0.1892
Final R indexes [all data]	R_1 = 0.0945, wR_2 = 0.2112
Largest diff. peak/hole / [e Å^{-3}]	0.85/−1.80

5.5 [Tc(^{Pyr}PNP^{tBu})(CO)$_2$(NC$_4$H$_4$)] (11)

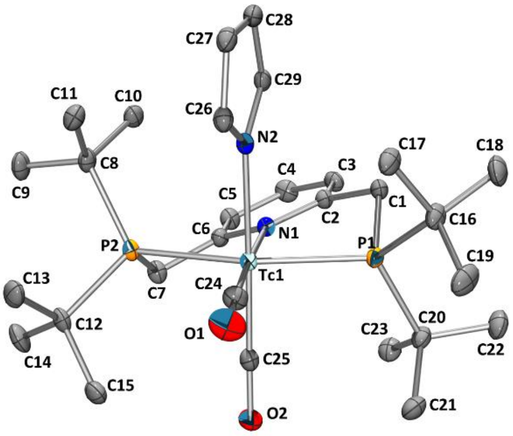

Figure S91: Ellipsoid displacement plot[11] of [Tc(^{Pyr}PNP^{tBu})(CO)$_2$(NC$_4$H$_4$)] (**11**). Ellipsoids represent 35% probability. Hydrogen atoms are omitted for clarity. A second molecule is present in the structure that is not shown for clarity as well.

Table S9: Tabulated values of selected bond lengths and angles in the crystal structure of [**11**].

	Selected bond lengths		Selected bond angles
Tc1–P1	2.4434(8) Å	P1–Tc1–C24	98.82(13)°
Tc1–P2	2.4790(9) Å	P2–Tc1–C24	103.10(12)°
Tc1–N1	2.201(3) Å	P1–Tc1–C25	90.97(11)°
Tc1–C24	1.896(4) Å	P2–Tc1–C25	89.38(11)°
Tc1–C25	1.885(4) Å	C24–Tc1–C25	86.67(17)°
Tc1–N2	2.222(3) Å	C24–Tc1–N1	177.31(15)°
N2–C26	1.375(5) Å	C25–Tc1–N1	91.24(13)°
N2–C29	1.373(5) Å	P1–Tc1–N2	90.46(7)°
C24–O1	1.155(5) Å	P2–Tc1–N2	90.28(7)°
C25–O2	1.159(5) Å	N2–Tc1–C25	177.00(13)°
P1–Tc1–N1	79.50(8)°	N2–Tc1–N1	91.61(11)°
P2–Tc1–N1	78.56(8)°	N2–Tc1–C24	90.50(15)°
P1–Tc1–P2	158.06(4)°		

Table S10: Crystallographic data of [Tc(^{Pyr}PNP^{tBu})(CO)$_2$(NC$_4$H$_4$)] (**11**)

Empirical formula	$C_{29}H_{47}N_2O_2P_2Tc$
Formula weight	615.62
Diffractometer	Rigaku OD XtaLAB Synergy, Dualflex, Pilatus 200K
Radiation	MoKα (λ = 0.71073 Å)
Temperature [K]	160
Crystal system	monoclinic
Space group	$P2_1$
a [Å]	11.4221(3)
b [Å]	10.8214(2)
c [Å]	13.3416(3)
α [°]	90
β [°]	112.269(3)
γ [°]	90
Volume [Å3]	1526.07(7)
Z	2
ρ_{calc} [g/cm^3]	1.340
μ [mm^{-1}]	0.604
F(000)	648
Crystal size [mm^3]	0.184 × 0.077 × 0.055
Crystal description	clear yellowish colorless plate
2Θ range for data collection [°]	3.854 to 57.928
Index ranges	$-14 \leq h \leq 14$, $-13 \leq k \leq 14$, $-17 \leq l \leq 17$
Reflections collected	21042
Independent reflections	6902 [R_{int} = 0.0274, R_{sigma} = 0.0302]
Reflections observed	6654
Criterion for observation	I>2σ (I)
Completeness to theta	99.94% to 28.9010°
Absorption correction	gaussian
Min./max. transmission	0.854/1.000
Data/restraints/parameters	6902/1/338
Goodness-of-fit on F^2	1.070
Final R indexes [I>=2σ (I)]	R_1 = 0.0296, wR_2 = 0.0744
Final R indexes [all data]	R_1 = 0.0311, wR_2 = 0.0751
Largest diff. peak/hole / [e Å^{-3}]	0.46/−0.27

5.6 [Tc(^{Pyr}PNP^{tBu})(CO)$_2$(N$_2$C$_3$H$_3$)] (12)

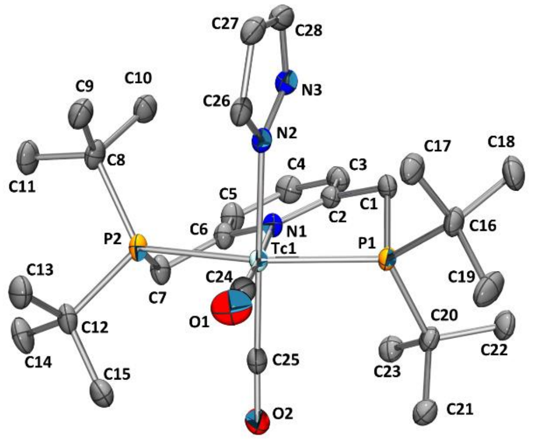

Figure S92: Ellipsoid displacement plot[11] of [Tc(^{Pyr}PNP^{tBu})(CO)$_2$(N$_2$C$_3$H$_3$)] (**12**). Ellipsoids represent 35% probability. Hydrogen atoms are omitted for clarity. A second molecule is present in the structure that is not shown for clarity as well.

Table S11: Tabulated values of selected bond lengths and angles in the crystal structure of [**12**].

	Selected bond lengths		Selected bond angles
Tc1–P1	2.4551(5) Å	P1–Tc1–P2	157.359(17)°
Tc1–P2	2.4515(5) Å	P1–Tc1–C24	102.71(6)°
Tc1–N1	2.2005(16) Å	P2–Tc1–C24	99.93(6)°
Tc1–C24	1.907(2) Å	P1–Tc1–C25	90.29(6)°
Tc1–C25	1.883(2) Å	P2–Tc1–C25	89.47(6)°
Tc1–N2	2.2204(15) Å	C24–Tc1–C25	89.61(9)°
N2–C26	1.350(3) Å	C24–Tc1–N1	178.06(7)°
C27–C28	1.388(3) Å	C25–Tc1–N1	91.88(7)°
C24–O1	1.148(3) Å	P1–Tc1–N2	89.37(4)°
C25–O2	1.161(3) Å	P2–Tc1–N2	92.28(4)°
N2–N3	1.365(2) Å	N2–Tc1–C25	176.26(8)°
P1–Tc1–N1	78.53(4)°	N2–Tc1–N1	91.70(6)°
P2–Tc1–N1	78.85(4)°	N2–Tc1–C24	86.84(7)°

Table S12: Crystallographic data of [Tc(^{Pyr}PNP^{tBu})(CO)$_2$($N_2C_3H_3$)] (**12**)

Empirical formula	$C_{28}H_{46}N_3O_2P_2Tc$
Formula weight	616.62
Diffractometer	Rigaku XtaLAB Synergy, Dualflex, HyPix
Radiation	CuKα (λ = 1.54184 Å)
Temperature [K]	160
Crystal system	monoclinic
Space group	$P2_1/n$
a [Å]	13.15990(10)
b [Å]	11.15210(10)
c [Å]	21.0469(2)
α [°]	90
β [°]	103.3540(10)
γ [°]	90
Volume [Å3]	3005.34(5)
Z	4
ρ_{calc} [g/cm^3]	1.363
μ [mm^{-1}]	5.115
F(000)	1296
Crystal size [mm^3]	0.22 × 0.08 × 0.055
Crystal description	clear yellowish orange block
2Θ range for data collection [°]	7.248 to 160.104
Index ranges	$-15 \leq h \leq 16$, $-14 \leq k \leq 14$, $-25 \leq l \leq 26$
Reflections collected	52710
Independent reflections	6520 [R_{int} = 0.0271, R_{sigma} = 0.0162]
Reflections observed	6351
Criterion for observation	I>2σ (I)
Completeness to theta	99.4% to 80.052°
Absorption correction	gaussian
Min./max. transmission	0.550/1.000
Data/restraints/parameters	6520/0/337
Goodness-of-fit on F^2	1.09
Final R indexes [I>=2σ (I)]	R_1 = 0.0266, wR_2 = 0.0692
Final R indexes [all data]	R_1 = 0.0271, wR_2 = 0.0696
Largest diff. peak/hole / [e Å^{-3}]	0.62/−0.69

6. References

(1) Boyd, G. E. Technetium and Promethium. *J. Chem. Educ.* **1959**, *36* (1), 3–14.

(2) Besmer, M. L.; Braband, H.; Schneider, S.; Spingler, B.; Alberto, R. Exploring the Coordination Chemistry of N_2 with Technetium PNP Pincer-Type Complexes. *Inorg. Chem.* **2021**, *60* (9), 6696–6701.

(3) Ignat'ev, N. V.; Barthen, P.; Kucheryna, A.; Willner, H.; Sartori, P. A Convenient Synthesis of Triflate Anion Ionic Liquids and Their Properties. *Molecules* **2012**, *17* (5), 5319–5338.

(4) Clark, R. C.; Reid, J. S. The Analytical Calculation of Absorption in Multifaceted Crystals. *Acta Crystallogr. Sect. A* **1995**, *51* (6), 887–897.

(5) CrysAlisPro (Version 1.171.40.68a). Rigaku Oxford Diffraction. Abingdon, Oxfordshire, England **2019**.

(6) Dolomanov, O. V.; Bourhis, L. J.; Gildea, R. J.; Howard, J. A. K.; Puschmann, H. OLEX2: A Complete Structure Solution, Refinement and Analysis Program. *J. Appl. Crystallogr.* **2009**, *42*, 339–341.

(7) Sheldrick, G. M. SHELXT - Integrated Space-Group and Crystal-Structure Determination. *Acta Crystallogr. Sect. A* **2015**, *71*, 3–8.

(8) Sheldrick, G. M. Crystal Structure Refinement with SHELXL. *Acta Crystallogr. Sect. C* **2015**, *71*, 3–8.

(9) Spek, A. L. PLATON SQUEEZE: A Tool for the Calculation of the Disordered Solvent Contribution to the Calculated Structure Factors. *Acta Crystallogr. Sect. C* **2015**, *71*, 9–18.

(10) Kalinowski, H.-O.; Berger, S.; Braun, S. *^{13}C-NMR-Spektroskopie*; Thieme: New York, **1984**.

(11) Farrugia, L. J. ORTEP-3 for Windows - a Version of ORTEP-III with a Graphical User Interface (GUI). *J. Appl. Crystallogr.* **1997**, *30*, 565–565.
